# Flavonoid compounds as a way to identify sources of carrot resistance to Alternaria leaf blight

**DOI:** 10.1007/s11032-025-01573-1

**Published:** 2025-06-14

**Authors:** Marie Louisa Ramaroson, Claude Emmanuel Koutouan, Angelina El Ghaziri, Raymonde Baltenweck, Patricia Claudel, Philippe Hugueney, Sébastien Huet, Anita Suel, Linda Voisine, Mathilde Briard, Jean Jacques Helesbeux, Latifa Hamama, Valérie Le Clerc, Emmanuel Geoffriau

**Affiliations:** 1 Institut Agro, Université d’Angers, INRAE, IRHS, SFR 4207 QUASAV, Angers, France; 2 Université de Strasbourg, INRAE, SVQV UMR-A 1131, Colmar, F-68000 France; 3 Faculté de santé – Département Pharmacie, Université d’Angers, Angers, France

**Keywords:** Marker-Assisted-Selection, Apigenin, Luteolin, Chrysoeriol, Specialized metabolite kinetics, UHPLC

## Abstract

Breeding varieties that are highly resistant to Alternaria leaf blight is crucial to enable carrot growers to drastically reduce their use of synthetic fungicides. Some sources of resistance have been identified in recent years, but in limited number and the genetic control as well as the screening for resistance remain complex and tedious. Flavonoid compounds have been reported to be involved in plant resistance to biotic or abiotic stresses. Their level of variation could therefore be a way of assisting screening activities for resistance. The aim of the study is to validate this link throughout the carrot growth cycle, in various environments and across a wide genetic diversity. A kinetic study showed that three flavonoid compounds are differentially accumulated between resistant and susceptible accessions as early as the 2-leaf stage and all along the plant development. Moreover, this differential is maintained throughout the potential infectious process in different environments. The analysis of a large range of accessions representing a very wide diversity of geographical origins, genetic structures, breeders and varietal types validates the link between resistance and the content in flavonoid compounds. These results open up extremely interesting prospects for the development of a marker-assisted early selection tool that would facilitate the screening and introgression of resistances into elite material, a complex task due to the polygenic control of resistance and biennial nature of the crop.

**Supplementary Information:**

The online version contains supplementary material available at 10.1007/s11032-025-01573-1.

The production of carrots, categorized as health-promoting vegetables recommended for consumption by the FAO, is increasingly influenced by social and regulatory changes (FAO 2020). While growers continue to rely on synthetic pesticides to secure their harvests, an increasing number of these pesticides are being prohibited, necessitating the proposal for alternative plant protection strategies. One of the most prevalent and detrimental pre-harvest diseases in carrot production is leaf blight, caused by *Alternaria dauci* fungus (*A. dauci*). This disease poses a significant threat to root development if established early in plant growth (Davis 2004) and can result in total losses of up to 100% during severe outbreaks, as harvesting is based on foliage grasping (Ben-Noon et al. 2001; Farrar et al. 2004; Boedo et al. 2008). Currently, commercial carrot varieties lack sufficient resistance levels to eliminate the need for fungicide treatments. The introgression of higher resistance traits from genetic resources into modern cultivars (Boedo et al. 2012) is a critical strategy. However, resistance to *A. dauci* is exclusively quantitative, characterized by several partial effect QTL (Le Clerc et al. 2014, 2015; Le Clerc and Briard 2020; Koutouan et al. 2023) and is presumably based on various resistance mechanisms (Lecomte et al. 2012; Koutouan et al. 2018, 2023; Le Clerc and Briard 2020), leading to a complex introgression process. To facilitate the introgression process and variety breeding, resistance markers with the following key characteristics would be advantageous: applicability to large plant populations (at the plantlet stage), reliability throughout the entire infection period and usability across different genetic backgrounds.

As summarised in recent reviews (Ramaroson et al. 2022; Das et al. 2024), flavonoids are specialised metabolites frequently reported to play a role in plant defence against biotic and abiotic stresses. Their biosynthesis and accumulation can be either constitutive or inducible in response to external stimuli. To be effective, maintaining an adequate level of these metabolites throughout the infection process is crucial (Ramaroson et al. 2022). However, flavonoid content in leaves can vary both quantitatively (Ancuceanu et al. 2017; Ryu et al. 2017; Petropoulos et al. 2018) and qualitatively (Omezzine et al. 2014) during different plant growth stages. A significant differential accumulation of three flavonoids was previously observed in the leaves of a susceptible (H1) and a partially resistant (I2) carrot accession to *A. dauci* at the (6–8)-leaf stage (Koutouan et al. 2018). The authors suggested these compounds as potential biomarkers of resistance. These three compounds are heterosides identified as apigenin-*7*-O-rutinoside (Api7R), luteolin-*7*-O-rutinoside (Lut7R) and chrysoeriol-*7*-O-rutinoside (Chry7R). The present study aims to characterize the accumulation patterns of these three flavonoids in carrot leaves at different phenological stages and assess their consistency across diverse genetic backgrounds to determine their potential usefulness in pre-breeding processes.

More specifically, this study aims to address the following questions: (i) At what earliest stage of carrot development can a metabolic contrast between susceptible and partially resistant accessions be observed? (ii) Is an early high metabolite accumulation sustained throughout vegetative growth? (iii) Does the relationship between metabolite content and disease resistance level depend on the genetic background of carrot accessions? (iv) Are Api7R, Lut7R and Chry7R good indicators of resistance usable in carrot pre-breeding process?

## Materials and methods

### Plant materials

Four successive trials were conducted with a total of 38 accessions of very diverse types and origins, as detailed in Table 1. Accessions were obtained from the QuaRVeg breeding program at IRHS in Angers, France, the UK Vegetable Genebank (Warwick University), or private breeding companies such as Vilmorin-Mikado, Nunhems, and HM-Clause. The commercial hybrids Presto, Valor and Bolero are reference varieties for susceptibility, intermediate resistance, and high partial resistance, respectively. Within breeding material the level of resistance to Alternaria Leaf Blight (ALB) varied from highly susceptible (e.g., H1) to high partially resistant (e.g., I2) accessions. These two accessions are S2 inbred lines from QuaRVeg breeding program, previously described by Le Clerc and her colleagues (2014, 2015, 2025) and Koutouan and his colleagues (2018, 2023).

Trial 1 aimed to address the first two research questions outlined above and therefore focused on developmental stages. For this purpose, the two reference S2 breeding lines, H1 and I2, were used. Trials 2 and 3 focused on the third question, which concern the influence of genetic background. The objective of Trial 2 was to investigate flavonoid content in resistant accessions. Accordingly, all six resistant accessions in our possession were used. Additionally, two susceptible controls were included to provide a point of comparison. Trial 3 aimed to examine flavonoid content in susceptible accessions. All accessions—except I2 and Lob 69— were therefore chosen for their high susceptibility to ALB and ensuring the inclusion of the most diverse genetic structures, varietal types and geographic origins. Finally, Trial 4 aimed to assess the reliability of the proposed test, serving as a proof of concept. Six accessions were chosen based on their high content of the three identified flavonoid compounds, while their resistance level was initially unknown. They were compared with two commercial references, Bolero and Presto, and two resistant breeding lines Lob 69 and Reg 21.

**Table 1:**
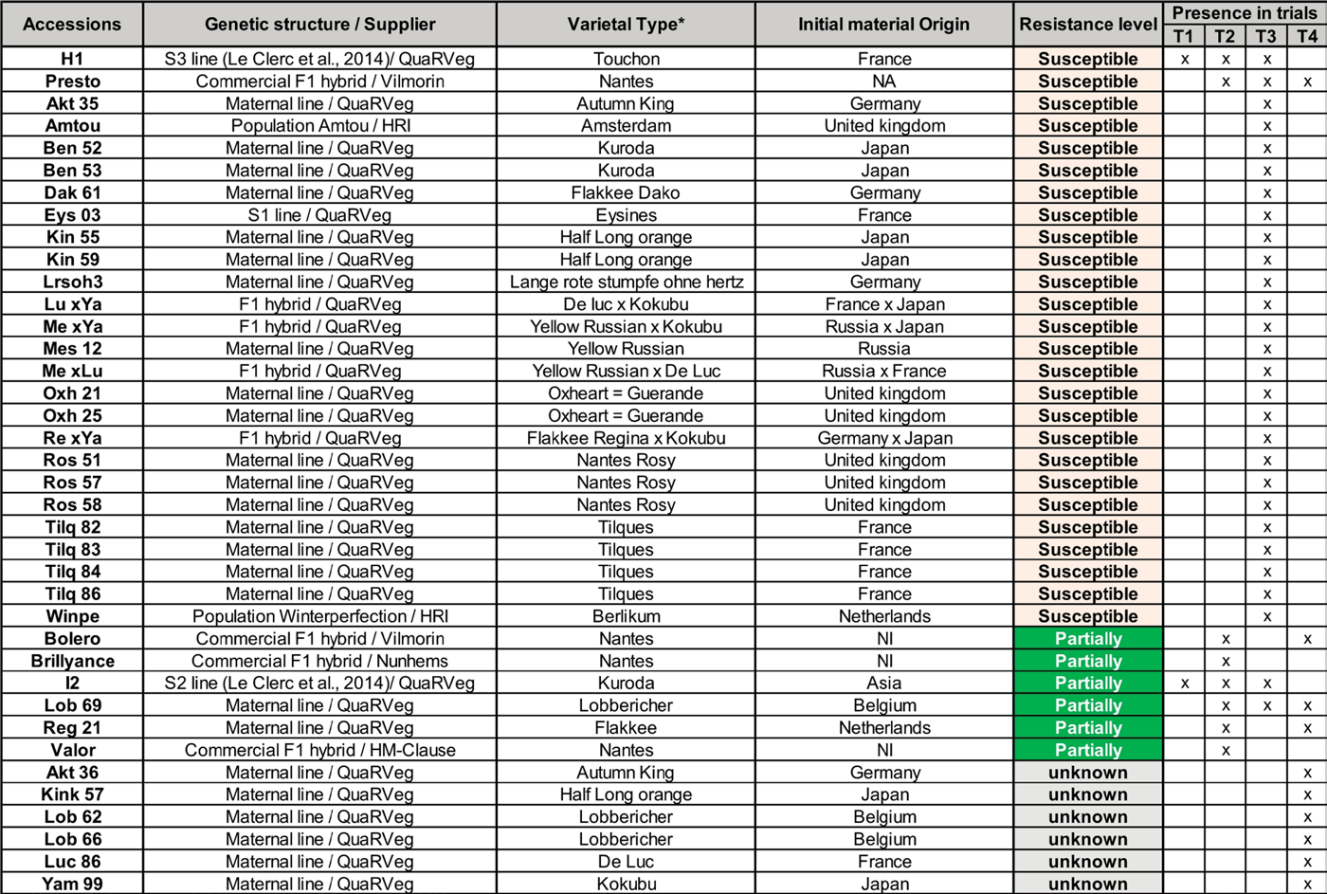
Origin and resistance level to ALB of Carrot accessions. Accessions Bolero and Presto are commercial references frequently grown by farmers in French Carrot production basins and used as references for ALB ‘resistance/susceptibility’ evaluation by the GEVES (Variety and seed study and control Group). * varietal types are described in literature (Geoffriau 2020; Simon and Grzebelus 2020). HRI = UK vegetable genebank (Warwick University). NI = No information

### Experimental designs and samplings

#### Trial 1 (1 susceptible H1 and 1 resistant I2)

Accessions H1 and I2 were grown from June to November from seeds sown at IRHS field in Angers, France (Latitude 47.4711; Longitude − 0.5518). The experimental design consisted of four replicates per accession, with each replicate comprising a 20-meter linear row, yielding approximately 1,600 plants per replicate and accession. Sampling was conducted at seven developmental stages: 2-, 3-, 4-, 5-, 6-, 9- and 12-leaf stages, based on the number of fully developed leaves. Four samples (one per replicate) were collected at each stage. Each sample was composed of leaves from eight different plants, randomly distributed along the row. From the 2- to 4-leaf stages, all leaves were harvested, whereas at later stages, only the three intermediate leaves were collected. Samples were immediately frozen in liquid nitrogen and transported from the field to the laboratory.

#### Trial 2 (6 resistant accessions and 2 susceptible controls)

Eight accessions (Table 1) were grown from June to November on the same location, but one year after Trial 1, using a randomized block design with four replicates. Within each block, seeds of each accession were sown in two rows of one linear meter (90 seed per row), aiming to obtain approximately 80 plants per row. Samples were collected at three developmental stages—2, 6 and 10 true leaves—following the same protocol described in Trial 1.

#### Trial 3 (26 susceptible diverse accessions and 3 resistant controls)

A total of 29 accessions (Table 1), including on one hand H1 and Presto and on the other hand I2, Reg 21 and Lob 69 (serving as references for susceptibility and resistance, respectively), were grown from June to November in Ychoux, a major commercial carrot production area with high *A. dauci* pressure (Latitude 44.3333; longitude − 0.9667, Les Landes, France) under standard commercial crop management conditions. Due to producer constraints, an only two-block experimental design was implemented. To ensure uniform disease pressure across the field, Presto was sown along the central row of a 1.20-meter-wide seedbed, with the two experimental blocks positioned on either side. Replicates of the evaluated accessions were randomly distributed within these two blocks, with each replicate consisting of 360 plants. As described by Koutouan and his colleagues (2018), this trial was conducted on a sandy soil field during the optimal carrot growth period. Pathogen attacks were predicted using Plant-Plus system^®^ (PPsystem) developed by Dacom (http://www.dacom.nl/), which integrates weather conditions, plant development, and pathogen concentration. Each replicate was harvested eight days after detection of natural infestation by PPsystem, as described by Koutouan and his colleagues (2018). Briefly, eight whole plants at the 6–8 leaf stage, distributed at various points along the row, were harvested and transported under cold conditions to the laboratory, where two intermediate leaves per plant were pooled for analysis.

#### Trial 4 (6 flavonoid rich accessions and 4 controls)

In preparation for Trial 4, the Api7R, Lut7R, and Chry7R content of 126 accessions grown in a breeding experimental field was determined. Based on this metabolic analysis, the breeding lines with the highest content were selected to assess whether their resistance level aligns with the hypothesis that high flavonoid content is an indicator of high resistance. The complete selection process is shown in Online Resource 6. Briefly, the leaf Api7R, Lut7R and Chry7R contents of each accession were evaluated from samples collected at the 6–8 leaf stage. To select the most enriched accessions, the 85 th percentile threshold was applied: For each metabolite, the 126 accessions were ranked based on their content of that metabolite. To retain only the accessions with the highest content, we set a threshold, considering only those with a content of at least 85% of the highest level detected (in I2) as acceptable. This selection resulted in a list of approximately 20 accessions for each metabolite. The final list was then defined as the intersection of these three lists, representing the highest-accumulating accessions for all three metabolites.

This approach led to the selection of six accessions whose resistance level to ALB was evaluated in Trial 4, alongside the two commercial references, Bolero and Presto, serving as commercial references for susceptibility and resistance, respectively, as well as Reg 21 and Lob 69, the two resistant breeding lines identified in Trial 2.

The total of 10 accessions (Table 1) were grown from March to June under greenhouse conditions in Angers, France (latitude 47.4711; longitude − 0.5518) as described by Le Clerc and her colleagues (2025). Briefly, five-liter pots were filled with Traysubstrat from Klasmann-147 Deilmann^®^ (Geeste, Germany). For each accession, 10 seeds were sown per pot in greenhouse. Then after 30 days, only seven seedlings were retained in each pot. Each pot containing seven plants was considered as one replicate per accession, with four replicates per accession. The photoperiod was set to 16 h of light and 8 h of darkness, with temperature maintained at 20 °C +/−2 during the day and 18 °C +/−2 at night. For inoculation, the inoculum of Ad P2 strain (moderate aggressiveness) was prepared following the method described by Pawelec and her colleagues (2006). Briefly, a fungal suspension in water with Tween 20 (0.05%) was filtered through two layers of cheesecloth, and the conidial concentration was adjusted to 4-5 × 10^3^ conidia/mL. Inoculation was performed using a hand-held sprayer. Humidity and heat were maintained by a plastic cover for two days to promote fungal infection. After pathogen inoculation, the greenhouse temperature was maintained at 23 °C during both night and day, with humidity controlled using a fogging system.

### Leaf extraction and flavonoid quantification

Leaf samples (without petioles) were ground into a fine powder in liquid nitrogen using a mortar and pestle, then freeze-dried using − 80 °C freezer and a SRK GT2/8 lyophilizer (VWR).

For flavonoid analysis, 10 mg of freeze-dried powder (in 2 mL screw-capped tubes) were extracted with 1.5 mL of methanol containing an internal standard, chloramphenicol (5 µg/mL), using ultrasound-assisted extraction (15 min bath). After centrifugation at 12,000 rpm for 10 min, 100 µL of the supernatant were collected for further analysis.

The same analytical conditions as described by Koutouan and his colleagues (2018) were applied. Briefly, supernatants were analysed using ultrahigh-performance liquid chromatography (UHPLC, Dionex Ultimate 3000; Thermo Fisher Scientific) equipped with a diode array detector (DAD) and an Exactive Orbitrap mass spectrometer (Thermo Fisher Scientific) with an electrospray ionization (ESI) source operating in positive mode. Spectra were acquired within a mass-to-charge ratio (*m/z*) range of 95–1200 atomic mass units (a.), using a resolution of 50,000 at *m/z* 200 a.m.u. The system was internally calibrated using dibutyl phthalate as a lock mass (*m/z* 279.1591), ensuring a mass accuracy of < 1 ppm.

A 1 µL aliquot of methanolic leaf extract was injected into a Nucleodur HTec column (150 × 2 mm, 1.8 μm particle size from Macherey-Nagel) maintained at 30 °C. The mobile phase consisted of acetonitrile/formic acid (0.1%, v/v) as eluent A and water/formic acid (0.1%, v/v) as eluent B with a flow rate of 0.25 ml/min. The gradient elution program was as follows: 0–4 min, 80–70% B; 4–5 min, 70–50% B; 5–6.5 min, 50% B; 6.5–8.5 min 50–0% B; 8.5–10 min 0% B. The exact mass and retention time of each metabolite were used for relative quantification using Xcalibur software (Thermo Fisher Scientific). The commercial Apigenin-7-*O*-glucoside standard (Sigma Aldrich, Saint-Quentin-Fallavier, France) was used to estimate metabolite content per gram of dry weight (DW).

Mass spectrometry data were retrieved for the three candidate compounds. A correction factor was applied to the peak area of each flavonoid, based on the amplitude of the response shift exhibited by the internal standard in each sample. Specifically, the corrected value of the metabolite peak area (M’) was obtained from the initial value (M) according to Eq. 1 where the ratio “IS/mean IS” was the ratio between the internal standard peak area in the sample (IS) and the mean of all internal standard peak areas in the complete data set (Mean IS).

1 $$\:{M}^{{\prime\:}}=M\times\:\left(2-\frac{\:IS}{Mean\:IS}\right)$$

### Api7R, Lut7R, and Chry7R description

Based on previous work (Koutouan et al. 2018), this work is focusing on three metabolites that belong to the large family of flavonoids, which share the generic carbon backbone structure C6-C3-C6. The flavonoid pathway derives from the phenylpropanoid pathway, and starts by the isomerization of naringenin chalcone to give the flavanone naringenin. Naringenin is in turn the common precursor of diverse subfamilies of flavonoids including flavones such as apigenin. Apigenin can be hydroxylated at the carbon in 3’ by a flavone 3’,5’-hydroxylase to produce luteolin. Luteolin can undergo a further transfer of a methyl group at the newly added 3’-OH by a 3’-*O*-methyltransferase to produce chrysoeriol. Then, from these three precursors, apigenin, luteolin, and chrysoeriol, heterosides respectively identified as apigenin-7-*O*-rutinoside (Api7R), luteolin-7-*O*-rutinoside (Lut7R) and chrysoeriol-7-*O*-rutinoside (Chry7R), can be obtained by the successive action 7-*O*- glycosyltransferases and rhamnosyltransferases. These pathways are described in the KEGG database (Kyoto Encyclopedia of Genes and Genomes, Kanehisa et al. 2017), and in several articles (Koutouan et al. 2018; Ramaroson et al. 2022). The three flavonoids of interest Api7R (C_27_H_30_O_14_), Lut7R (C_27_H_30_O_15_), and Chry7R (C_28_H_32_O_15_) can be detected and quantified using their characteristic ions with the following mass-to-charge ratio (*m/z*) and retention times (RT) in the present experimental conditions: *m/z* 579.1708 (4.44 min), *m/z* 595.1657 (3.46 min), and *m/z* 609.1812 (4.62 min) respectively.

### Disease severity scoring

Trials 1, 2 and 3 were conducted under natural *A. dauci* infestation pressure. For trial 1, symptoms observed at the end of the season, confirmed that I2 was resistant and H1 was highly blighted, as expected. For Trials 2 and 3, disease symptoms were assessed in October using a 0 (no symptom) to 9 (totally blighted plants) disease severity scale, as developed by Pawelec and her colleagues (2006). A minimum of 50 plants per accession per block was considered as one biological replicate. For Trial 4, disease severity was evaluated 14 days after inoculation across 4 replicates using the same 0 to 9 scale.

### Statistical analysis

All statistical analyses were performed using R statistical software (R version 4.4.1; R Core Team 2024). When no specific package is mentioned, functions from the base *stats* package were used (e.g., for ANOVA, Shapiro-Wilk, or Kolmogorov-Smirnov tests). For pairwise comparisons, the *emmeans* and *cld* functions from the *emmeans* (Lenth 2024) and *multcomp* (Hothorn et al. 2008) packages were used. Box-Cox transformations were performed using the *powerTransform* function from the *car* package (Fox and Weisberg 2019).

For Trial 1, the influence of the two factors (accession and developmental stage) on the response variables (content of the three flavonoids) was evaluated using a two-way ANOVA with an interaction model. The assumptions underlying the use of ANOVA were checked as follows: (1) independence of the observations (ensured during data collection), (2) normality of residual distribution (Kolmogorov-Smirnov test), and (3) homogeneity of residual variances (assessed using a residuals plot against fitted values, where a homogeneous scatter plot with no visible pattern indicates homoscedasticity). If ANOVA assumptions were not met, a Box-Cox transformation was applied, and the assumptions were rechecked on the transformed data. In cases where the interaction affect was not significant (as for Api7R and Lut7R), an additive two-way ANOVA was used. The equivalence between the two models (with and without interaction) was assessed using ANOVA.

When the effect of a factor was significant (*p*-value < 0.05), multiple pairwise comparisons between groups were performed using Tukey’s *post hoc* test. Variations in metabolite content during development were analysed separately for each accession. Differences between accessions were highlighted in the ANOVA table, as only two accessions were considered in this analysis. A step-by-step analysis of the Trial 1 dataset is provided in Online Resource 1. Metabolite quantities, estimated from calibration curves established with the Apigenin-7-*O*-Glucoside standard, are detailed in Online Resource 2.

For Trial 2, metabolic values underwent the same data correction as in Trial 1 (Eq. 1). Correspondence analyses were performed, considering metabolite contents and disease scores as qualitative variables. Metabolite content was categorized into four metabolite enrichment levels (classes) based on quartile distribution: content < 25%, content]25–50%], content]50–75%] and content > 75%. Disease severity scores were divided into three classes: low score, intermediate score, and high score relating to [3–5.2],]5.2–6.6] and]6.6–8] disease score intervals respectively. A Chi-squared test was used to assess the dependence of these two variables (significance threshold: *p*-value < 0.05), followed by Correspondence analysis to examine associations between their levels (Online Resource 3). Correspondence Analysis, including the Chi-squared test, was performed using the *CA* function from the *FactoMineR* package (Lê et al. 2008).

The disease score data were analysed using a linear mixed-effects model, with accession as fixed factor and repetition as a random factor to account for variability across repeated measurements. The model was fitted using the *lmer* function from the *lmerTest* package in R (Kuznetsova et al. 2017) (Online Resource 4).

For metabolite data, a two-way ANOVA with a two-factor interaction model was performed after confirming that the Block factor was not significant in this case (Online Resource 5). The assumptions underlying the use of ANOVA were verified before analysis.

For Trial 3, due to grower constraints, only two replicates per accession were conducted, and for some accessions, only one. Consequently, a descriptive analysis was chosen, focusing on visualizing flavonoid content as a function of accession and disease score.

For Trial 4, an one-way ANOVA of the disease severity score as a function of the accessions was performed after confirming that the repetition factor was not significant in this case (Online Resource 6). The assumptions underlying the use of ANOVA were verified before analysis. Pairwise comparisons between accessions were conducted using Tukey’s test.

Lastly, for each of Trial 1, 2, 3, and 4 pairwise correlations between the three-flavonoid contents were assessed using Pearson’s correlation coefficient (Online Resource 7).

## Results

### Quantitative differences between H1 and I2 during plant development (Trial 1)

**Fig. 1:**
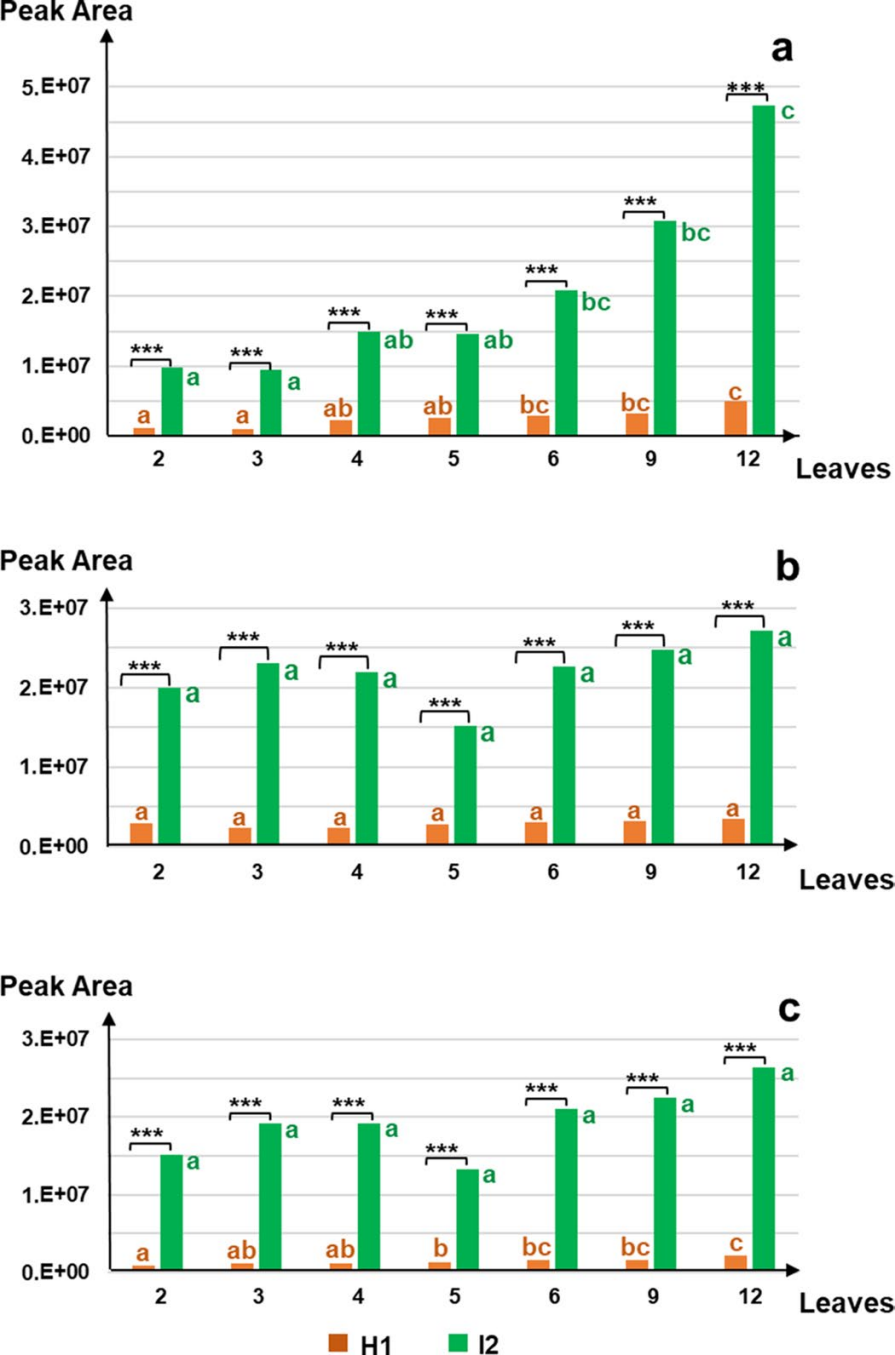
Accumulation kinetics of apigenin-7-*O*-rutinoside (**a**), luteolin-7-*O*-rutinoside (**b**), and chrysoeriol-7-*O*-rutinoside (**c**) in the leaves of the susceptible carrot accession H1 coloured in brown and the partially resistant accession I2 in green. The X-axes represent the plant development as a number of expanded true leaves. The Y-axes represent peak area values from mass-spectrometry-based quantification (arbitrary unit), following a Box-Cox transformation (Online Resource 1). Data were obtained from four replicates per accession, with each replicate composed of leaves from eight different plants. Letters near the boxes indicate group differentiation based on Tukey’s test, comparing metabolite content across different developmental stages (number of leaves) within the same accession. Significance symbols denote statistical significance levels for metabolite content between the two accessions at each developmental stage: *p* < 0.001 ‘***’

The metabolic difference between H1 and I2 was observable and significant from the earliest developmental stage (2-leaf stage) and persisted until the latest stage (12-leaf stage), regardless of the metabolite (Fig. 1, Online Resource 1). Although Api7R (Fig. 1a) and Chry7R (Fig. 1c) contents gradually increased in H1, the levels of all three metabolites in H1 remained significantly lower than those of I2 at all stages.

The average flavonoid content in each accession, relative to the dry weight of carrot leaves, was estimated (Online Resource 2). Regardless of the developmental stage and compound, the average content ranged from approximately 30–200 µg/g DW in H1 and 300–2000 µg/g DW in I2. This indicates that the resistant accession (I2) contains about ten times more of the three flavonoids than the susceptible accession (H1).

### Correspondence between disease severity and metabolite enrichment in eight accessions (Trial 2)

**Fig. 2:**
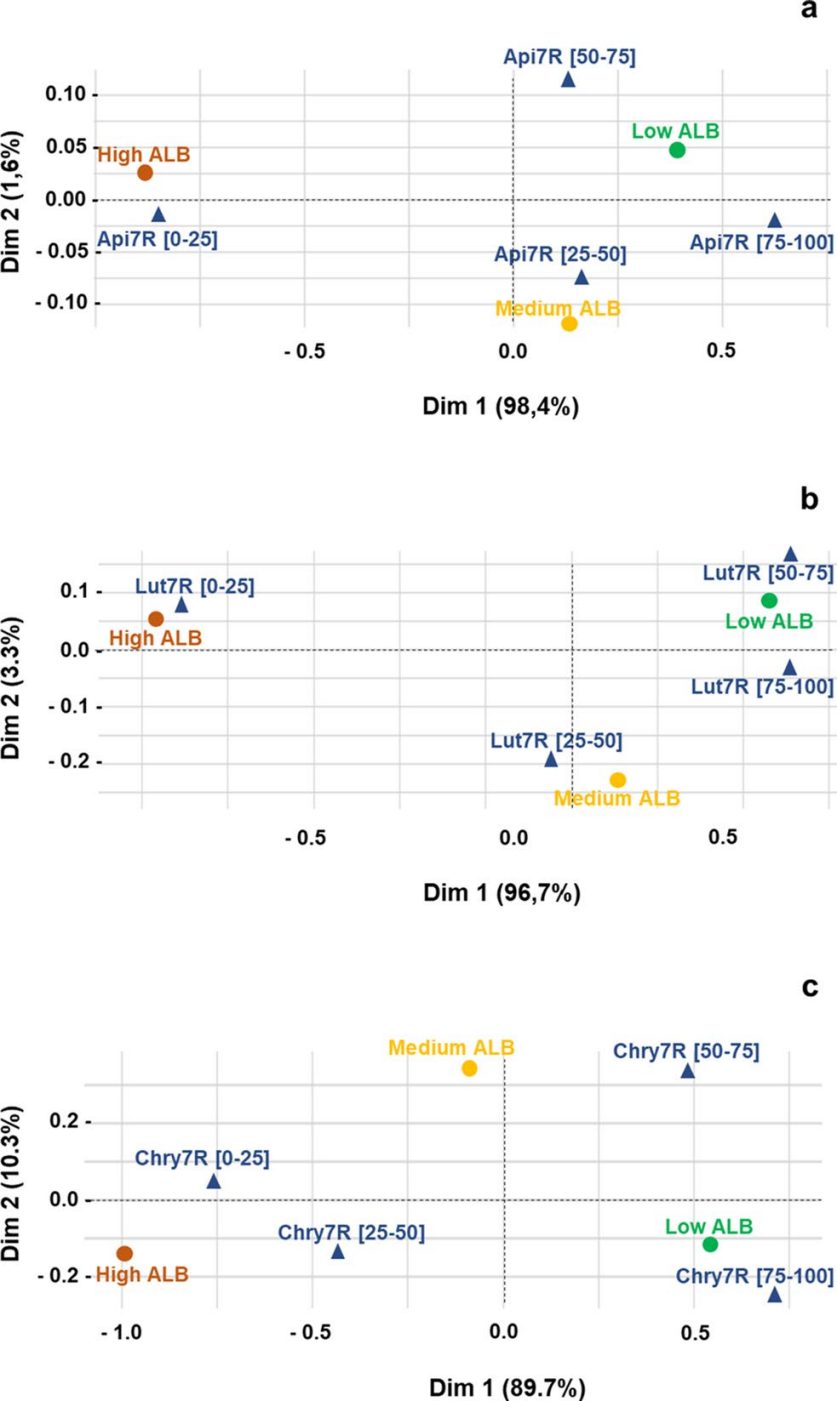
Correspondence analysis maps illustrating the relationships between three levels of Alternaria leaf blight (ALB) severity and four classes of apigenin-7-*O*-rutinoside (Api7R), luteolin-7-*O*-rutinoside (Lut7R), and chrysoeriol-7-*O*-rutinoside (Chry7R) contents. Api7R, Lut7R, and Chry7R are presented in panels a, b, and c respectively. Data were obtained from four replicates of eight varieties. The positions of the four quartiles of metabolite content are indicated by dark blue triangles, while the positions of the three ALB severity levels are represented by solid circles coloured brown, yellow, and green, corresponding to high, intermediate, and low severity, respectively

The relation between disease severity and metabolite enrichment was assessed through a Correspondence Analysis (CA, Fig. 2). The first dimension of the CA map (Online Resource 3 A) was primarily explained by low and high ALB severity scores, as these two classes had high contributions and quality of representation. In contrast, axis 2 was associated with the intermediate ALB severity score. Api7R enrichment (Fig. 2A and Online Resource 3 A) displayed an inverse distribution relative to disease severity. Very low Api7R content (< 25%, i.e., first quartile) was positively associated with high ALB disease score (low resistance), whereas high Api7R content (> 75%, i.e., fourth quartile) was linked to low ALB severity (high resistance). Accessions with intermediate Api7R content (second quartile ([25–50]) co-localized with an intermediate ALB severity score. A similar association pattern was observed between Lut7R content and disease severity (Fig. 2B and Online Resource 3B). For Chry7R, a content below the median (i.e., < 50%) was associated with high ALB severity, with a stronger effect observed for values within the first quantile (< 25%) (Fig. 2C and Online Resource 3 C). Consistent with previous observations for Api7R and Lut7R, a Chry7R content above the median (> 50%) was associated with a low ALB disease score, with the strongest effect at the highest content (> 75%). However, content within the [50–75] interval mostly corresponded to an intermediate ALB severity level, as reflected in the label distribution along the second dimension of the factor map (Online Resource 3 C).

Overall, this Correspondence Analysis highlighted that accessions with the lowest metabolite content tended to develop the most severe ALB disease, whereas those with relatively higher levels of each candidate flavonoids exhibited greater resistance. A more detailed analytical approach is required to confirm these patterns for each accession in the set.

### Comparison of accessions: ALB symptoms and candidate metabolite content (Trial 2)

As expected, H1 and Presto were the two most susceptible accessions, while all other accessions, including I2 and Bolero, exhibited partial resistance (Online Resource 4). These results were consistent with previous findings for H1 and I2, as well as for commercial hybrids such as Presto and Bolero (Boedo et al. 2010; Le Clerc et al. 2014; Koutouan et al. 2018).

**Fig. 3:**
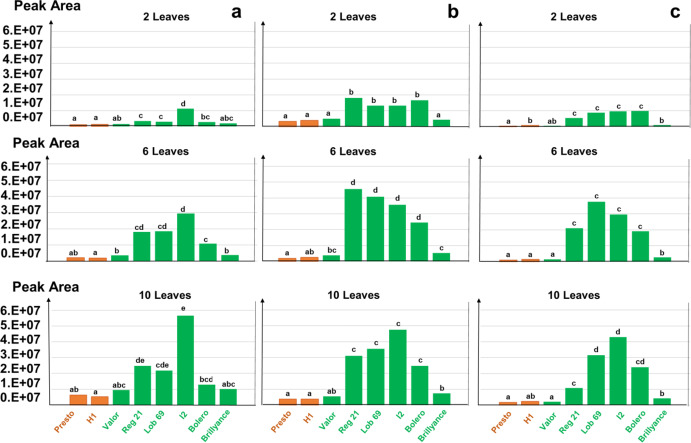
Accumulation of apigenin-7-*O*-rutinoside (**a**), luteolin-7-*O*-rutinoside (**b**), and chrysoeriol-7-*O*-rutinoside (**c**) in the leaves of eight carrot accessions at 3 developmental stages (from top to bottom, 2 leaves, 6 leaves, and 10 leaves). On X-axes bar colours, indicate ALB resistance (Online Resource 4) with susceptible accessions (Presto and H1) represented in brown and partially resistant accessions (Valor, Reg 21, Lob 69, I2, Bolero, and Brillyance) represented in green. The Y-axes represent peak area values from mass-spectrometry-based quantification (arbitrary unit). Data were obtained from four replicates of the eight varieties. Letters denote group differences based on Tukey’s test applied to Box-Cox transformed data (Online Resource 5b) with error threshold = 0.05

The differences in metabolite level between accessions were analysed using ANOVA. All necessary conditions for ANOVA were validated after applying a Box-Cox transformation to the data. The results indicated that the two factors (accession and developmental stage) and their interaction were highly significant (Online Resource 5). Although metabolite contents increased over time across all accessions, all significant differences observed between accessions at the 2-leaf stage remained significant at the 6- and 10-leaf stages. (Fig. 3 and Online Resource 5).

### Candidate metabolite content in a large set of susceptible accessions (Trial 3)

Overall, two significantly groups were identified. On one hand, Reg 21, Lob 69, I2 and Bolero formed one group, contrasting with Presto and H1 on the other. Regardless of their developmental stage, H1 and Presto did not differ significantly in their Api7R, Lut7R and Chry7R contents (Fig. 3 and Online Resource 5). Regardless of the metabolite, these two susceptible accessions accumulated very low levels of these compounds. As expected, they differed significantly from the resistant accession I2 as early as the 2-leaf stage, and this difference persisted throughout plant development. This pattern followed the trend distinguishing more diseased from less diseased accessions in the correspondence analysis (Fig. 2) and aligned with the accumulation kinetics shown in Fig. 1. Flavonoid contents for Valor and Brillyance were generally similar to those in Presto and H1. In contrast, the other resistant accessions (Reg 21, Lob 69 and Bolero) were typically similar to I2 exhibiting high metabolite content, or were intermediate with a lower content.

**Fig. 4:**
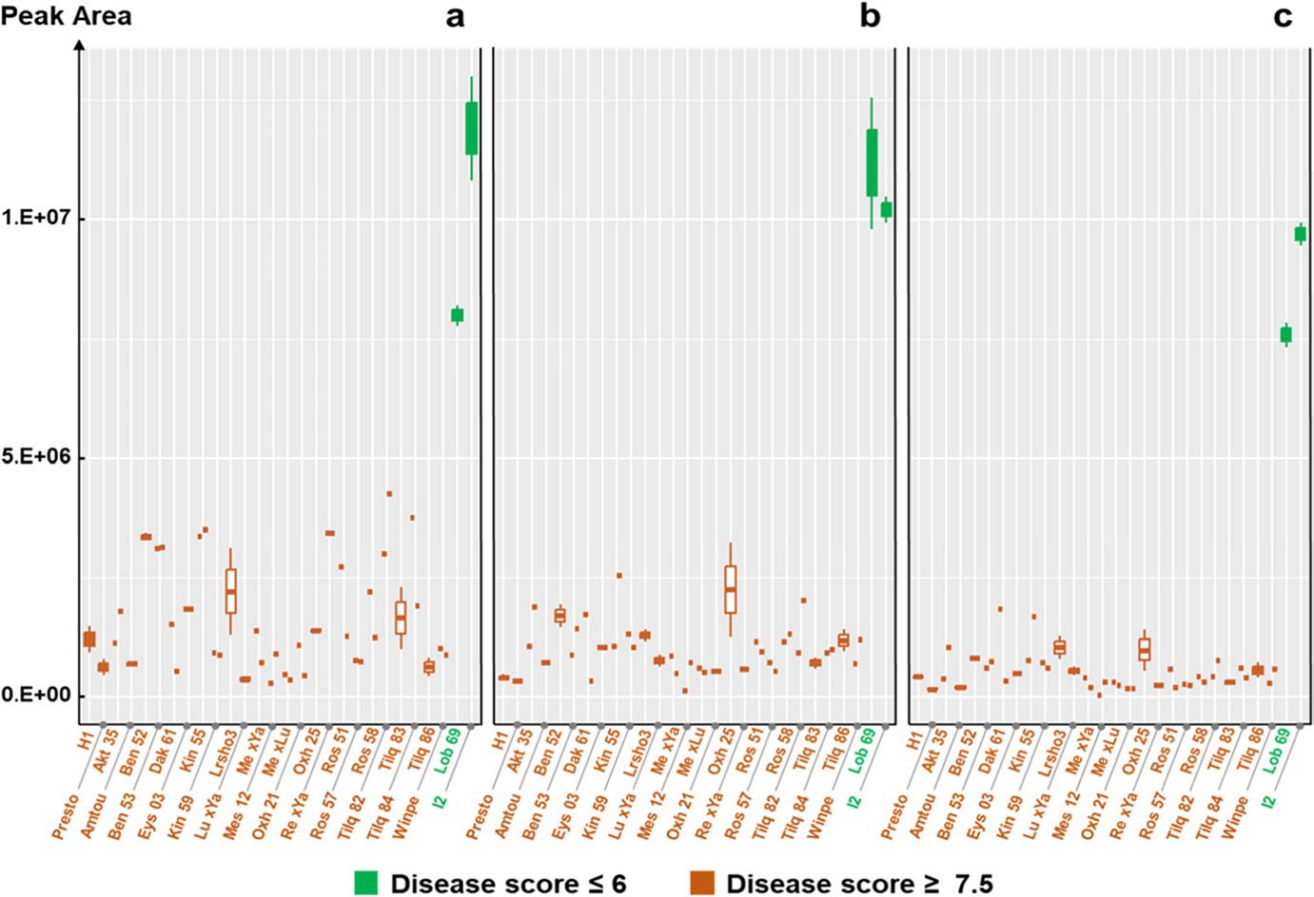
Accumulation of apigenin-7-*O*-rutinoside (**a**), luteolin-7-*O*-rutinoside (**b**), and chrysoeriol-7-*O*-rutinoside (**c**) in the leaves of 29 carrot accessions at the 6–8-leaf developmental stage. The X-axes represent the different accessions. The Y-axes represent peak area values from mass-spectrometry-based quantification (arbitrary unit). The solid-coloured boxes represent the four controls: H1 and Presto for the susceptible accessions, and Lob 69 and I2 for the resistant ones. Data were obtained from two field replicates of each accession. Disease severity was scored using a 0 (no symptom) to 9 (totally blighted plants) disease severity scale, as developed by Pawelec and her colleagues (2006). Green indicates partial resistance (≤ 6) while brown indicates a high susceptibility to ALB (up to 9). Except for the two resistant controls, I2 and Lob 69 (on the right), all other accessions were chosen for their high susceptibility to ALB

All reference accessions exhibited the expected disease development scores: I2 and Lob 69 were partially resistant, showing low damage (disease score ≤ 6), whereas H1and Presto were highly susceptible and severely blighted (disease scores 8 and 9). As anticipated, all other accessions were also highly susceptible, with scores overpassing 7.5. The 26 susceptible accessions had consistently low levels of the three flavonoids (Fig. 4). In Trial 3, as previously observed in Trial 1, I2 exhibited metabolite contents more than ten-fold higher than those of any susceptible accession.

### Application of the concept to breeding material (Trial 4)

Among the 126 experimental accessions for which the content in Api7R, Lut7R, and Chry7R was determined, eight accessions with the highest contents were selected. Two of them (Reg 21 and Lob 69) had already been identified in Trial 2. Therefore, they served, in Trial 4, as additional controls alongside Bolero and Presto.

**Fig. 5:**
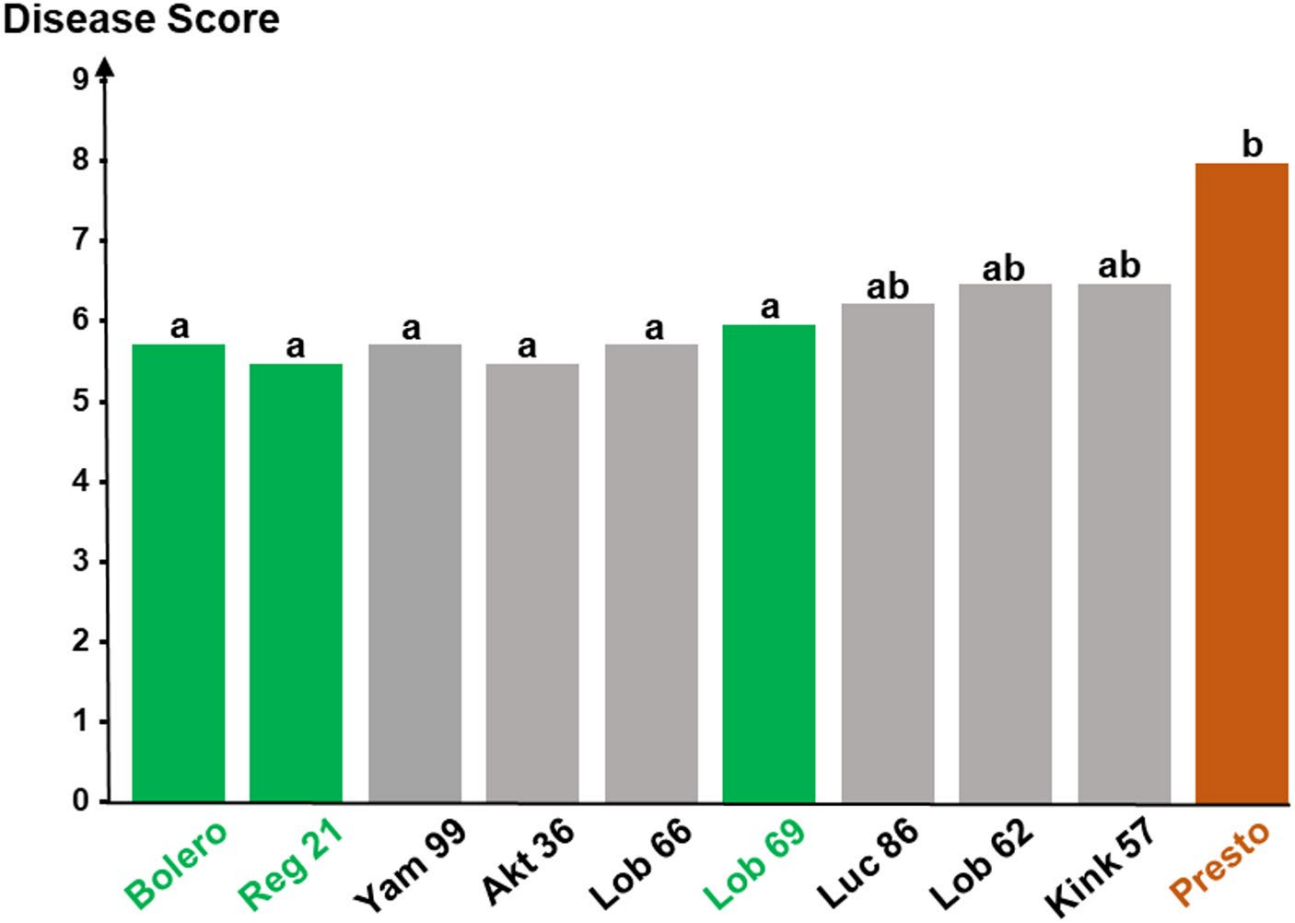
Disease scores of 10 carrot accessions following inoculation with *A. dauci*. The X-axis represents the different accessions. The Y-axis represents disease severity scored using a 0 (no symptom) to 9 (totally blighted plants) disease severity scale, as developed by Pawelec and her colleagues (2006). Data were obtained from four replicates of each accession. Green indicates resistant controls (Bolero, Reg 21 and Lob 69), brown represents the susceptible control (Presto) and grey denotes accessions selected for their high Api7R, Lut7R, and Chry7R contents in the leaves, with initially unknown resistance level (Online Resource 6). Letters indicate group differences based on Tukey’s test (Online Resource 6) with an error threshold of 0.05

Disease scores of all grey-selected accessions (Fig. 5) were not statistically different from those of the resistant controls (letter a). However, three of these accessions were also not significantly different from the susceptible control Presto (letter b). Therefore, the first three accessions (e.g. Yam 99, AKT 36, and Lob 66) can be classified as resistant lines, while the remaining ones (Luc 86, Lob 62, and Kink 57) are classified as intermediate-resistant lines. These results from a wide breeding panel supports the concept that high flavonoid content is an indicator of higher resistance level to *A. dauci*.

### Correlations between Api7R, Lut7R and Chry7R contents

Regardless of the dataset, all calculated correlations (Online Resource 7) were high for Api7R/Lut7R and Api7R/Chry7R varying from 0.69 to 0.88, and very high for Lut7R/Chry7R (from 0.92 to 0.99). All correlations were highly significant (*p* values varying from 2.70^e−13^ to 2.64^e−86^).

## Discussion

Very early accumulation of flavonoids in plant tissues has been reported to enhance the defence mechanisms of more resistant plant accessions against pathogens (Ardila et al. 2013). For example, a study on kaempferol glycosidic derivatives in barley (*Hordeum vulgare*) spikelets, comparing resistant and susceptible accessions to Fusarium head blight (FHB), demonstrated fold changes similar to those observed in the present study (Bollina et al. 2010). These findings aligns with observations in sorghum (*Sorghum bicolor* (L.) Moench), which similarly accumulates flavonoids—specifically from the flavanol group—to resist *Alternaria* spp. and *Fusarium* spp. (Melake-Berhan et al. 1996). In carrot, a significant differential accumulation of three flavonoids was also observed in the leaves of a susceptible (H1) and a partially resistant (I2) accessions to *A. dauci* (Koutouan et al. 2018). However, in none of these cases has the role of these metabolites in resistance been conclusively proven.

Given the very limited number of known sources of ALB resistance and the need to identify new ones for the improvement of elite lines, the availability of reliable measurable factors associated with resistance could be a major asset. The present study aimed to evaluate the feasibility of developing an early screening test based on flavonoid quantification. Even if these molecules have not yet been proven to confer resistance directly, their strong association with resistance could facilitate the exploration of genetic resources more effectively.

In previous studies, differences in ALB resistance and metabolite content between two contrasting carrot accessions were observed at the 6–8 leaf stage (Koutouan et al. 2018). In the present study, the accumulation of the three flavonoid-rutinosides in carrot leaves was monitored from the 2-leaf to the 12-leaf stages to assess earlier and sustained differences between the susceptible H1 and partially resistant I2. The collected data were essential to determine whether this difference could be detected at an earlier stage and/or beyond the previous assessment time point.

Results showed that I2 accumulated approximately 10 times more of the three flavonoid-rutinosides than H1 as early as the 2-leaf stage. This early differential suggests that flavonoid quantification could be applied to young plants (2-leaf stage) in large-scale breeding programs. This approach enables the comparison of numerous breeding lines by growing them in a same tray or plot simultaneously, providing an efficient, non-invasive screening method that does not require pathogen inoculation.

The relatively high levels of Api7R, Lut7R and Chry7R in I2 remained stable until 12-leaf stage or even gradually increased (in the case of Api7R). Regardless of the metabolite, the difference between H1 and I2 accessions remained statistically significant throughout the entire studied period of plant development (approximately three months).

This stability, observed in Trials 1 (with two accessions) and 2 (with eight accessions), represents an important criterion for the validation of a screening tool. This finding ensures flexibility in its application. Depending on climate and soil conditions, plant development can vary drastically. If sampling had to be conducted at a very specific stage, transferring the method from our laboratory to a breeding company could have been more challenging. However, since the differences between accessions were maintained across different developmental stages, the primary requirement is to ensure that all plants being compared are harvested at the same stage.

If ever Api7R, Lut7R and Chry7R metabolites are not only associated with resistance but also contribute to it, earliness and stability of high accumulation in the leaves of I2, Reg 21, Lob 69, and Bolero could be key factors in these accessions with strong resistance to *A. dauci*. This sustained accumulation could provide protection throughout the infection process, from 4-5 leaf stage to the 12-leaf stage.

Soil type, fertilization, sunlight exposure, high temperatures, and other factors are known to influence flavonoid accumulation in plant photosynthetic tissues, including in carrot (Tattini et al. 2000; Nithia et al. 2005; Ibrahim et al. 2006; Ceoldo et al. 2009; Agati et al. 2011; Seljåsen et al. 2012; Ulrich et al. 2015; Del Valle et al. 2018). Despite variations in environmental conditions, the magnitude of the quantitative metabolic difference between H1 and I2 accessions observed in the present study was fully consistent with Koutouan’s findings at 6–8 leaf stage in 2018. This consistency confirms the absence of genotype x environment interactions for flavonoid content, as suggested by Koutouan and his colleagues (2018). According to these authors, while specialized metabolite levels in a given genotype may vary between environments and years, the relative ranking of genotypes remains stable across different locations and time periods. Therefore, it can be concluded that the flavonoid-based test proposed in the present study can be applied across different locations, provided that all accessions being compared are grown under the same conditions.

For routine use, potential markers must be robust across diverse genetic backgrounds. Therefore, in this study, the co-occurrence of a higher metabolite accumulation and improved performance against *A. dauci* was investigated in carrot accessions selected for partial resistance and representing diverse genetic backgrounds, all distinct from I2.

Although, as mentioned above, the known sources of resistance remain extremely limited—explaining the small number of accessions tested in Trial 2—a correspondence analysis including the six resistant accessions available confirmed that low candidate metabolite content was associated with a high disease score, whereas higher metabolite content was associated with a lower disease score. A detailed analysis of these accessions further revealed that, beyond I2, three other partially resistant accessions could also be significantly differentiated from H1 at early, intermediate, and late stages of the vegetative growth, based on flavonoid content. This allowed us to conclude that the observed link between flavonoids and resistance in I2 was not specific to this resistance source, thus making the test suitable for identifying other resistance sources.

Valor and Brillyance did not cluster with the other resistant accessions but instead grouped with the susceptible varieties Presto and H1. These results suggest that alternative resistance mechanisms are involved in these varieties. This was not surprising, as several resistance mechanisms beyond flavonoid accumulation have been previously identified. For example, physical barriers or resistance mechanisms involving terpenes and polyacetylenes have been described as mediators of ALB resistance in carrots and may contribute to plant defence (Lecomte et al. 2012; Koutouan et al. 2018, 2023; Le Clerc and Briard 2020).

In the absence of a larger number of resistant accessions available for testing in Trial 2, we aimed to determine whether high levels of the three metabolites were likely to ensure a good level of resistance. In other words, we verified that no susceptible accession exhibited a high metabolite content.

All 26 susceptible accessions displayed low levels of Api7R, Lut7R, and Chry7R, compared with the resistant controls. This suggests that screening for high metabolite contents could indeed serve as effective tool for excluding susceptible material, which is particularly valuable in breeding programs.

To validate the proposed test, we applied it to 126 breeding lines to identify potential resistant ones. Indeed, the six selected ones based on the metabolic content proved to be resistant: three of them exhibited resistance level comparable to resistant controls, while the remaining three were classified as intermediate resistant.

The metabolic test took just a few weeks, whereas a proper assessment of the resistance level of such a panel of accessions in a greenhouse tests (with four replicates) or in field (with uncertain natural infection) would have been time-consuming and costly.

Finally, the contents of threes molecules were found to be very highly correlated. This result is fully consistent with their biosynthesis pathway, as described in the KEGG « Flavone and flavonol biosynthesis » pathway KEGGmap00944 (Kanehisa et al. 2017) and proposed by Koutouan and his colleagues (2018). Apigenin, as an initial substrate, is likely responsible for the accumulation patterns of the three branches leading to Api7R, Lut7R, and Chry7R metabolites. Chrysoeriol (Chry) is thought to derive directly from Luteolin (Lut).

Since a major concern in carrot breeding is the search for a “shortcut” method to reduce the duration and scale of field and greenhouse experiments for identifying accessions with high resistance level, we can conclude that assessing Api7R, Lut7R or Chry7R contents could provide an early, reliable, efficient and high-throughput tool for selecting strong resistance candidates.

In practice, high levels of these three flavonoids could serve as a selectable trait for predicting resistance level of accessions. Our results support this approach proposed in other systems, such as the *Dianthus caryophyllus* L.-*Fusarium oxysporum* f. sp. *dianthi* L. pathosystem (Ardila et al. 2013), and are consistent with the findings of Galeotti and his colleagues (2008), who successfully distinguished cultivars through the quantification of flavonoid-glycosides.

As shown in the present study, the resistance observed in certain varieties such as Valor or Brillyance is not associated with high flavonoid content and therefore must rely on a different mechanism. Further research will focus on exploring alternative resistance mechanisms and developing a similar approach to screen for resistant material. This approach could be based, for instance on terpenoid content, as suggested by Koutouan and his colleagues (2018, 2023). If successful, developing a dual-selection method based on the flavonoid and terpene contents would strengthen selection criteria in carrot breeding programs by combining resistance mechanisms.

Developing markers based on the genes responsible for Api7R, Lut7R and Chry7R accumulation, which requires further research, will enable the transformation of this tool into a more conventional genetic tool for breeders. Finally, elucidating the underlying mechanisms, particularly the ability of these compounds to induce defence genes or their potential biocidal effect on *A. dauci* will provide insights into the role played by these compounds.

## Supplementary Information

Below is the link to the electronic supplementary material.

Supplementary Material 1

Supplementary Material 2

Supplementary Material 3

Supplementary Material 4

Supplementary Material 5

Supplementary Material 6

Supplementary Material 7

## Data Availability

All data generated or analyzed during this study are whether included in this published article and its seven supplementary information files or available from the corresponding author on reasonable request.
