# Supplementary material for: Flavonoid compounds as a way to identify sources of carrot resistance to Alternaria leaf blight
Source: Mol Breed. 2025 Jun 14;45(6):55. doi: 10.1007/s11032-025-01573-1 (PMC12167411; doi:10.1007/s11032-025-01573-1)
Supplement: Supplementary file 1 — Supplementary Material 1 [file 11032_2025_1573_MOESM1_ESM.docx]

**Three flavonoids biomarkers of Carrot resistance to Alternaria leaf blight: ACCUMULATION PATTERN AT DIFFERENT PHENOLOGICAL STAGES AND CONSISTENCY ACROSS DIVERSE GENETIC BACKGROUNDS**

**Molecular breeding**

Marie Louisa Ramaroson*^1^, Claude Emmanuel Koutouan*^1^, Angelina El Ghaziri^1^, Raymonde Baltenweck^2^, Patricia Claudel^2^, Philippe Hugueney^2^, Sébastien Huet^1^, Anita Suel^1^, Linda Voisine^1^, Mathilde Briard^1^, Jean Jacques Helesbeux^3^, Latifa Hamama^1^, Valérie le Clerc^1^, Emmanuel Geoffriau^1,§^

1 Institut Agro, Université d’Angers, INRAE, IRHS, SFR 4207 QUASAV, Angers, France

2 Université de Strasbourg, INRAE, SVQV UMR-A 1131, F-68000 Colmar, France

3 Université de Strasbourg, INRAE, SVQV UMR-A 1131, F-68000 Colmar, France

§ Correspondence: [emmanuel.geoffriau@institut-agro.fr](mailto:emmanuel.geoffriau@institut-agro.fr); Tel : +33-(0)2 41 22 54 31

* The first two authors contributed equally to the paper

Online Resource 1: Metabolic data analysis of Trial 1 (H1 susceptible and I2 resistant accessions)

Table of contents

[Api7R 2](#_Toc181007960)

[Two-way ANOVA with interaction 2](#_Toc181007961)

[Postulates verification 2](#_Toc181007962)

[Box-Cox transformation 3](#_Toc181007963)

[Two-way ANOVA without interaction 5](#_Toc181007964)

[Box-Cox transformation 6](#_Toc181007965)

[Models comparison 8](#_Toc181007966)

[Pairwise comparison 8](#_Toc181007967)

[Lut7R 9](#_Toc181007968)

[Two-way ANOVA with interaction 9](#_Toc181007969)

[Postulates verification 9](#_Toc181007970)

[Box-Cox transformation 10](#_Toc181007971)

[Two-way ANOVA without interaction 12](#_Toc181007972)

[Postulates verification 12](#_Toc181007973)

[Box-Cox transformation 13](#_Toc181007974)

[Models comparison 15](#_Toc181007975)

[Pairwise comparison 15](#_Toc181007976)

[Chry7R 16](#_Toc181007977)

[Two-way ANOVA with interaction 16](#_Toc181007978)

[Postulates verification 16](#_Toc181007979)

[Box-Cox transformation 17](#_Toc181007980)

[Pairwise comparison 19](#_Toc181007981)

**Description**: Trial 1 data consists of two factors: Accessions (H1 and I2) and Developmental stage (12 levels). The dependent variables are three flavones: Api7R, Lut7R, and Chry7R. These variables are quantitative and continuous. Four repetitions are observed for each combination of the two factors, allowing us to study the interaction between the factors. In this appendix, we detail all the steps of data analysis. The results are presented in Figure 2 of the manuscript.

table(donnees$Accessions, donnees$Developmental_Stage)

2 leaves 3 leaves 4 leaves 5 leaves 6 leaves 9 leaves 12 leaves
 H1 4 4 4 4 4 4 4
 I2 4 4 4 4 4 4 4

# Api7R

## Two-way ANOVA with interaction

Mod1=lm(Api7R ~Accessions*Developmental_Stage, data=donnees)

### Postulates verification

res1=residuals(Mod1);
ks.test(res1, "pnorm", 0, sd(res1))

Exact one-sample Kolmogorov-Smirnov test

data: res1
D = 0.2227, p-value = 0.006397
alternative hypothesis: two-sided

plot(Mod1,1)


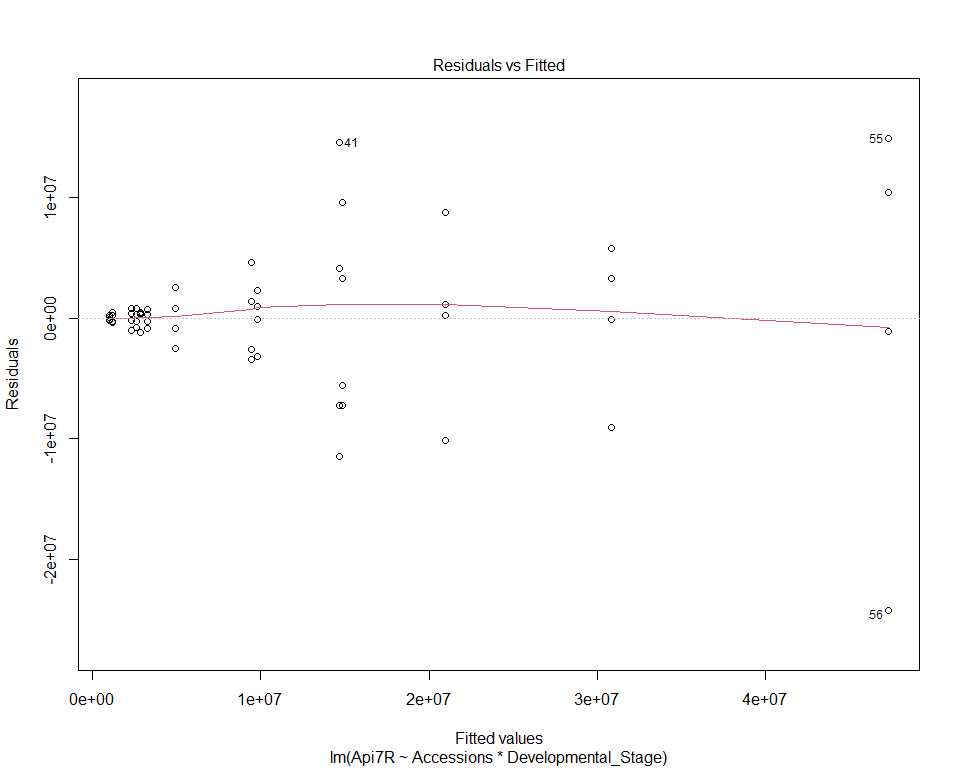


plot(Mod1,2)


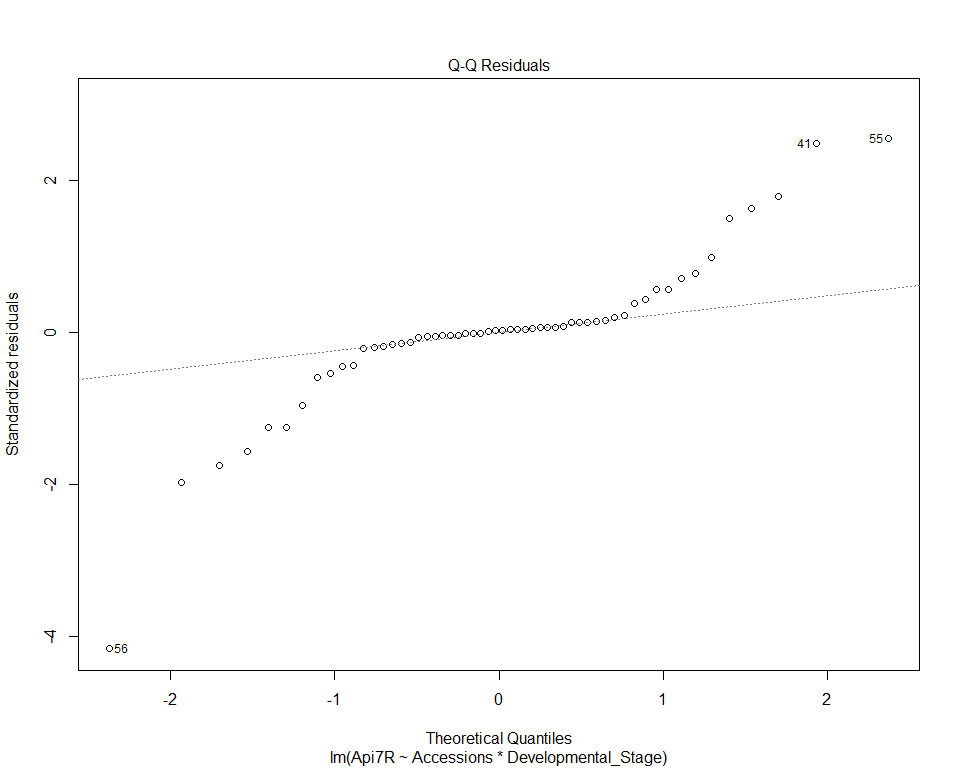


### Box-Cox transformation

summary(p1 <- powerTransform(Mod1))
mydata_bc <- transform(donnees, Api7R_bc=bcPower(Api7R,coef(p1)))

Mod1_bc <- lm(Api7R_bc~ Accessions*Developmental_Stage,
 data=mydata_bc)
ks.test(residuals(Mod1_bc),"pnorm",0, sd(residuals(Mod1_bc)))

Exact one-sample Kolmogorov-Smirnov test

data: residuals(Mod1_bc)
D = 0.12024, p-value = 0.3639
alternative hypothesis: two-sided

plot(Mod1_bc,1)


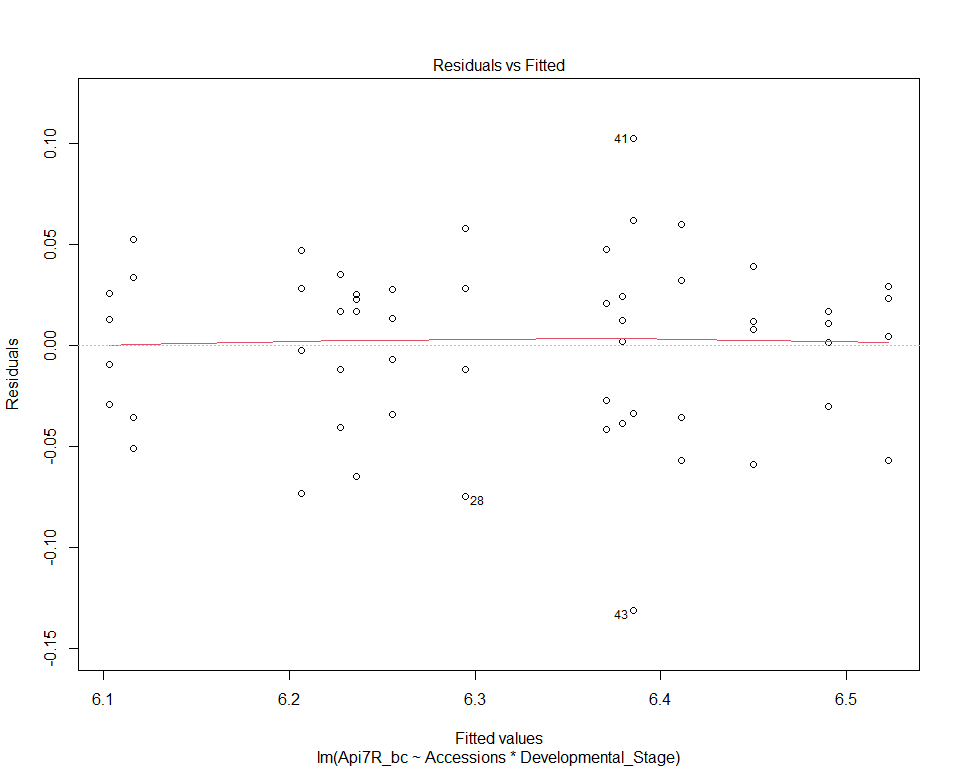


plot(Mod1_bc,2)


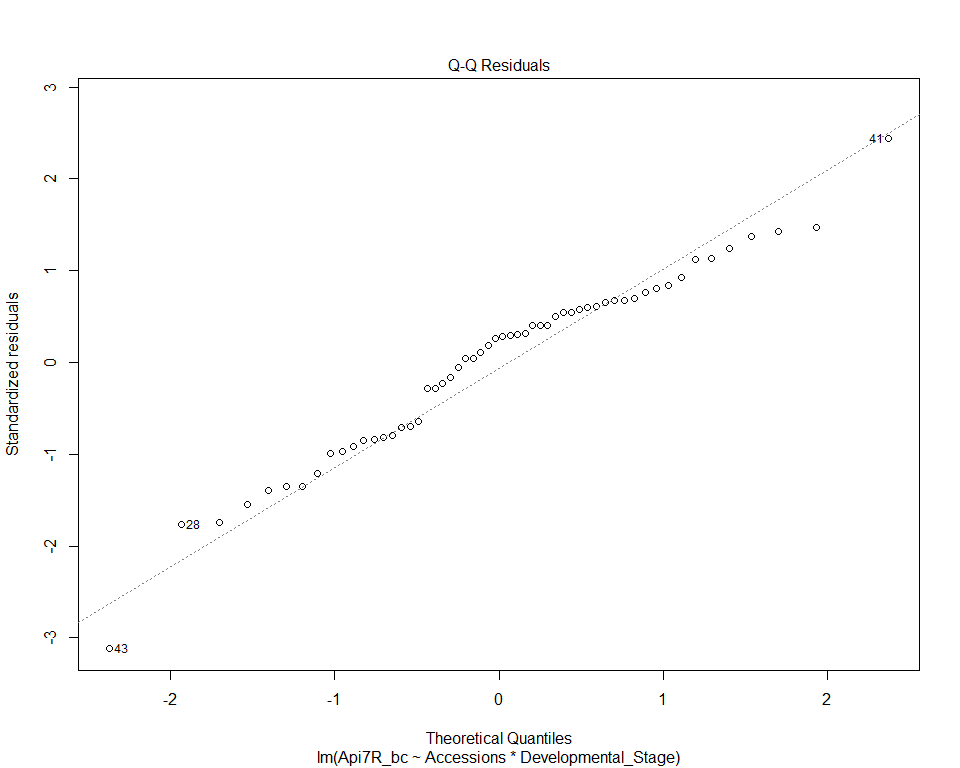


anova(Mod1_bc)

Analysis of Variance Table

Response: Api7R_bc
 Df Sum Sq Mean Sq F value Pr(>F)
Accessions 1 0.70469 0.70469 298.6007 < 2.2e-16 ***
Developmental_Stage 6 0.18923 0.03154 13.3640 2.084e-08 ***
Accessions:Developmental_Stage 6 0.01675 0.00279 1.1831 0.3338
Residuals 42 0.09912 0.00236
---
Signif. codes: 0 '***' 0.001 '**' 0.01 '*' 0.05 '.' 0.1 ' ' 1

## Two-way ANOVA without interaction

Mod1bis=lm(Api7R ~Accessions+Developmental_Stage, data=donnees)
res=residuals(Mod1bis)
ks.test(res,"pnorm", 0, sd(res))

Exact one-sample Kolmogorov-Smirnov test

data: res
D = 0.11447, p-value = 0.4237
alternative hypothesis: two-sided

plot(Mod1bis,2)


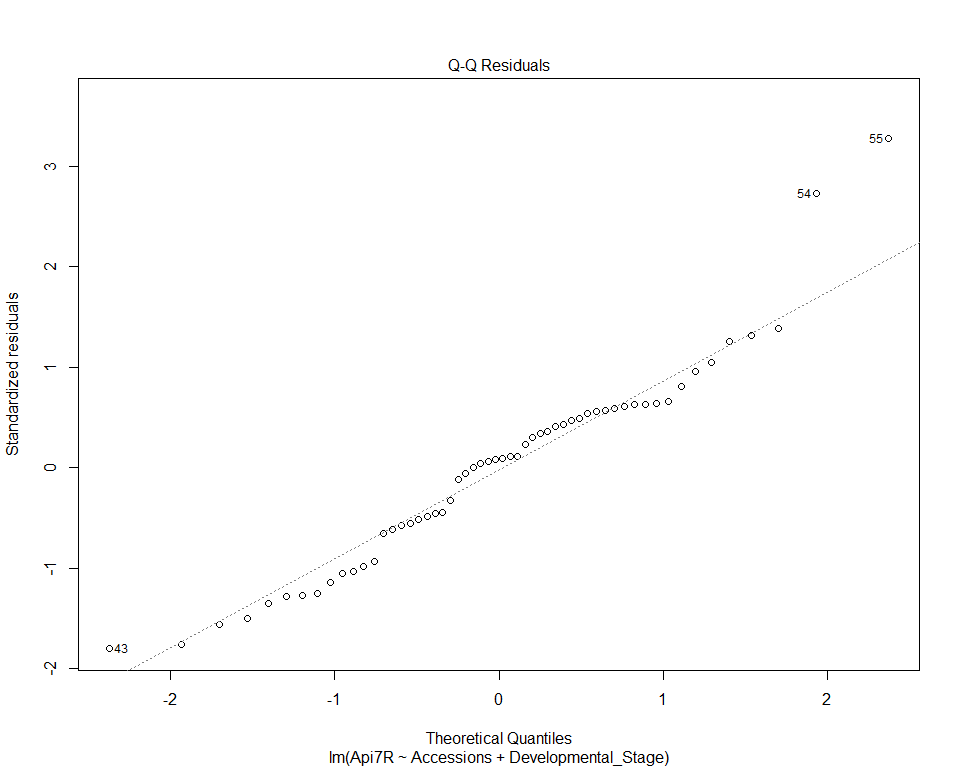


plot(Mod1bis,1)


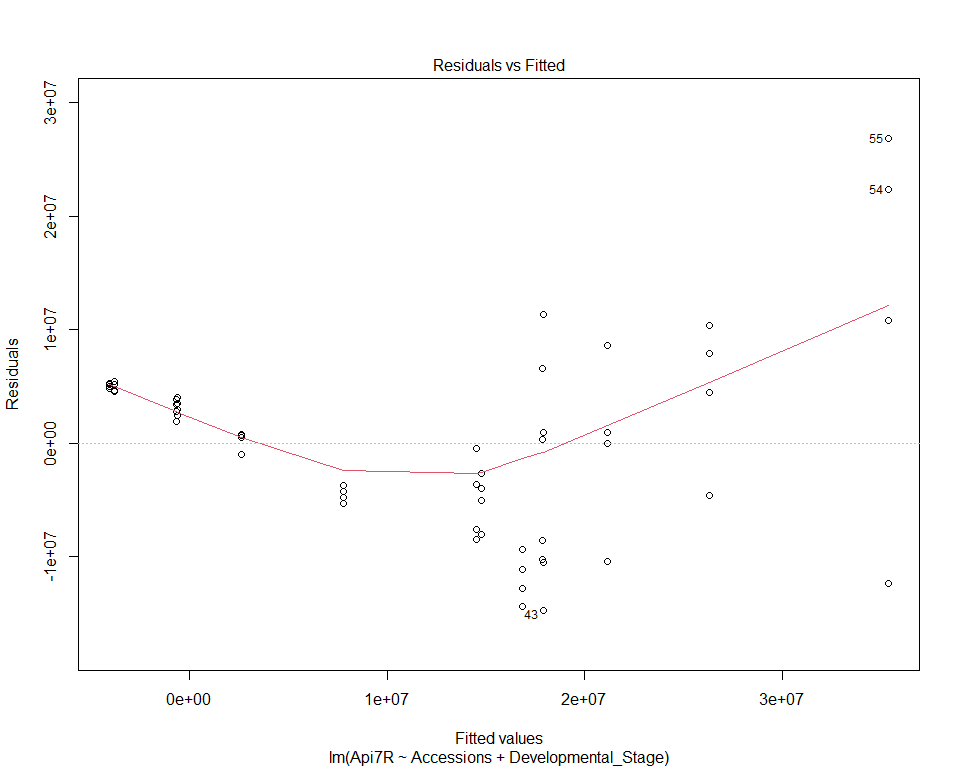


### Box-Cox transformation

summary(p2 <- powerTransform(Mod1bis))
mydata_bc <- transform(donnees, Api7R_bc=bcPower(Api7R,coef(p2)))

Mod1bis_bc <- lm(Api7R_bc~ Accessions+Developmental_Stage, data=mydata_bc)
res=residuals(Mod1bis_bc)
ks.test(res,"pnorm", 0, sd(res))

Exact one-sample Kolmogorov-Smirnov test

data: res
D = 0.14015, p-value = 0.2014
alternative hypothesis: two-sided

plot(Mod1bis_bc,1)


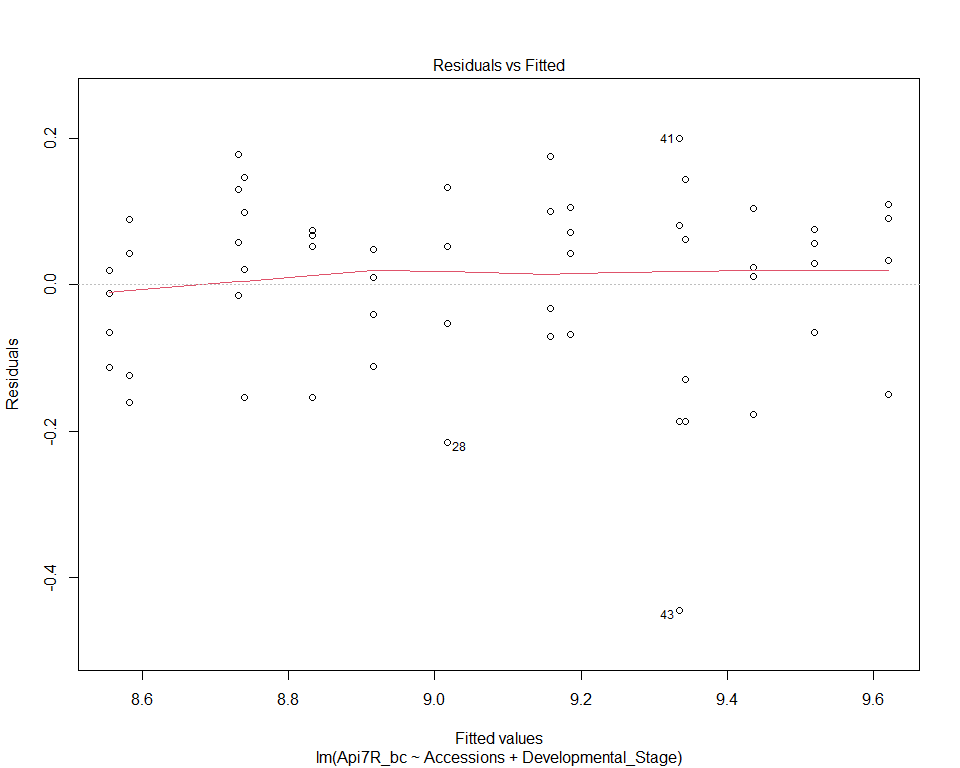


plot(Mod1bis_bc,2)


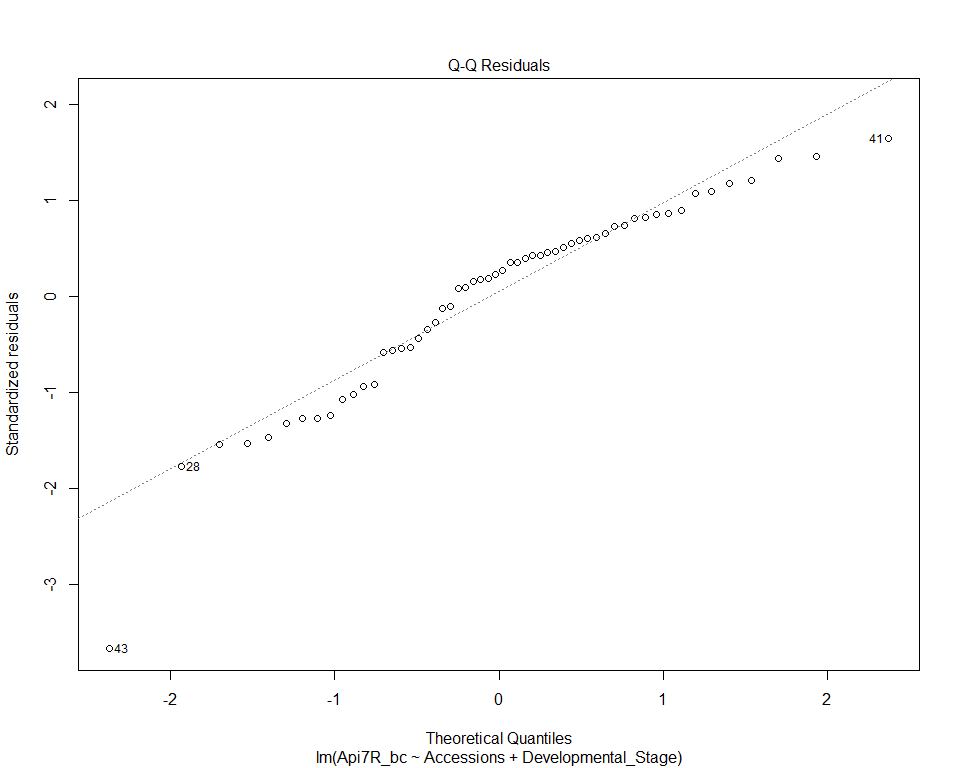


anova(Mod1bis_bc)

Analysis of Variance Table

Response: Api7R_bc
 Df Sum Sq Mean Sq F value Pr(>F)
Accessions 1 5.0751 5.0751 295.151 < 2.2e-16 ***
Developmental_Stage 6 1.3598 0.2266 13.181 9.388e-09 ***
Residuals 48 0.8254 0.0172
---
Signif. codes: 0 '***' 0.001 '**' 0.01 '*' 0.05 '.' 0.1 ' ' 1

### Models comparison

model_complet= aov(Api7R_bc~ Accessions*Developmental_Stage,data=mydata_bc)
model_rduit=aov(Api7R_bc ~Accessions+Developmental_Stage, data=mydata_bc)

anova(model_complet,model_rduit)

Analysis of Variance Table

Model 1: Api7R_bc ~ Accessions * Developmental_Stage
Model 2: Api7R_bc ~ Accessions + Developmental_Stage
 Res.Df RSS Df Sum of Sq F Pr(>F)
1 42 0.72242
2 48 0.82536 -6 -0.10294 0.9975 0.4397

###

### Pairwise comparison

emm_Api7R_1 <- emmeans(Mod1bis_bc, pairwise ~ Developmental_Stage |Accessions)
cld_Acc=cld(emm_Api7R_1, Letter="abcdefghijk")
cld_df <- as.data.frame(cld_Acc)
cld_df_ordered <- cld_df %>%
 arrange(factor(Accessions, levels = levels(donnees$Accessions)),
 factor(Developmental_Stage, levels = levels(donnees$Developmental_Stage)))
cld_df_ordered

Developmental_Stage Accessions emmean SE df lower.CL upper.CL .group
 2 leaves H1 8.582970 0.04956237 48 8.483319 8.682622 a
 3 leaves H1 8.555990 0.04956237 48 8.456338 8.655642 a
 4 leaves H1 8.740268 0.04956237 48 8.640617 8.839920 ab
 5 leaves H1 8.732216 0.04956237 48 8.632565 8.831868 ab
 6 leaves H1 8.832815 0.04956237 48 8.733163 8.932466 bc
 9 leaves H1 8.916744 0.04956237 48 8.817092 9.016396 bc
 12 leaves H1 9.017844 0.04956237 48 8.918192 9.117496 c

2 leaves I2 9.185057 0.04956237 48 9.085405 9.284709 a
 3 leaves I2 9.158076 0.04956237 48 9.058425 9.257728 a
 4 leaves I2 9.342355 0.04956237 48 9.242703 9.442007 ab
 5 leaves I2 9.334303 0.04956237 48 9.234651 9.433955 ab
 6 leaves I2 9.434901 0.04956237 48 9.335249 9.534553 bc
 9 leaves I2 9.518831 0.04956237 48 9.419179 9.618483 bc
 12 leaves I2 9.619931 0.04956237 48 9.520279 9.719583 c

Confidence level used: 0.95
P value adjustment: tukey method for comparing a family of 7 estimates
significance level used: alpha = 0.05
NOTE: If two or more means share the same grouping symbol,
 then we cannot show them to be different.
 But we also did not show them to be the same.

# Lut7R

## Two-way ANOVA with interaction

Mod2=lm(Lut7R~Accessions*Developmental_Stage, data=donnees)

### Postulates verification

res2=residuals(Mod2)
ks.test(res2, "pnorm", 0, sd(res2))

Exact one-sample Kolmogorov-Smirnov test

data: res2
D = 0.23952, p-value = 0.002599
alternative hypothesis: two-sided

plot(Mod2,1)


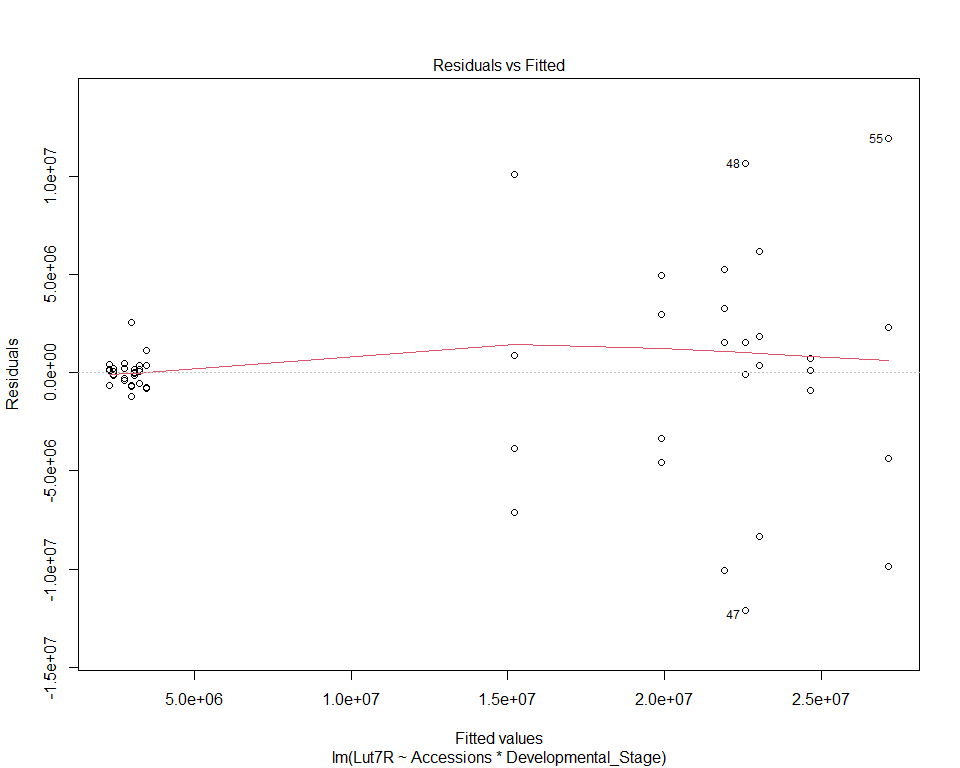


plot(Mod2,2)


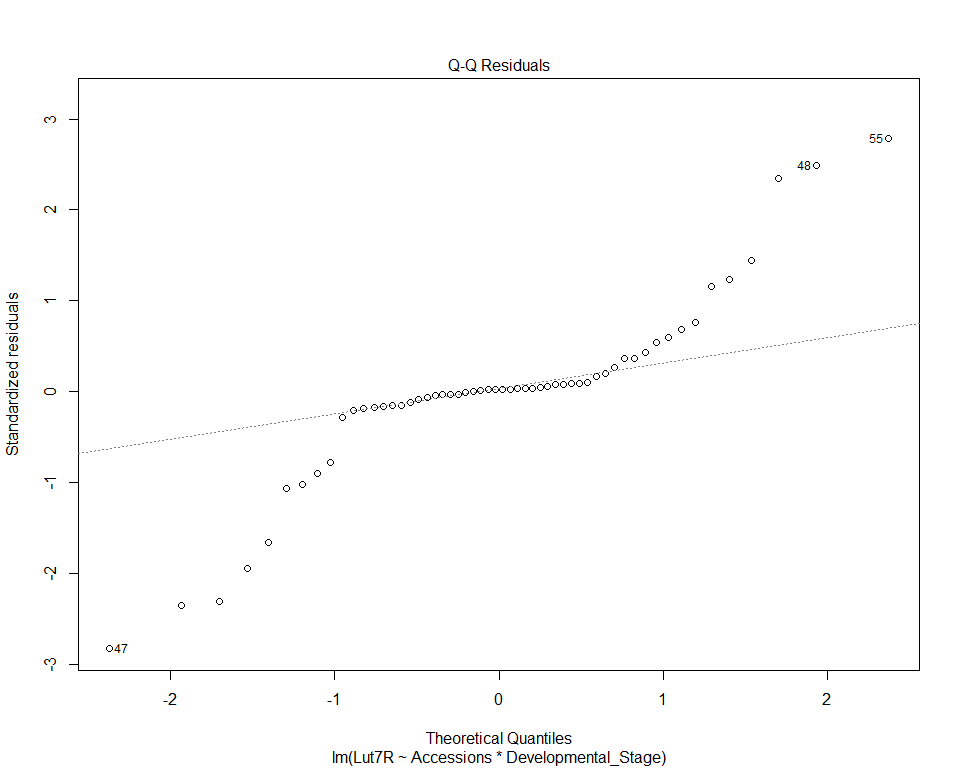


### Box-Cox transformation

summary(p2 <- powerTransform(Mod2))
mydata2_bc <- transform(donnees, Lut7R_bc=bcPower(Lut7R,coef(p2)))

Mod2_bc <- lm(Lut7R_bc~ Accessions*Developmental_Stage,data=mydata2_bc)
ks.test(residuals(Mod2_bc), "pnorm", 0, sd(residuals(Mod2_bc)))

Exact one-sample Kolmogorov-Smirnov test

data: residuals(Mod2_bc)
D = 0.10162, p-value = 0.5744
alternative hypothesis: two-sided

plot(Mod2_bc,1)


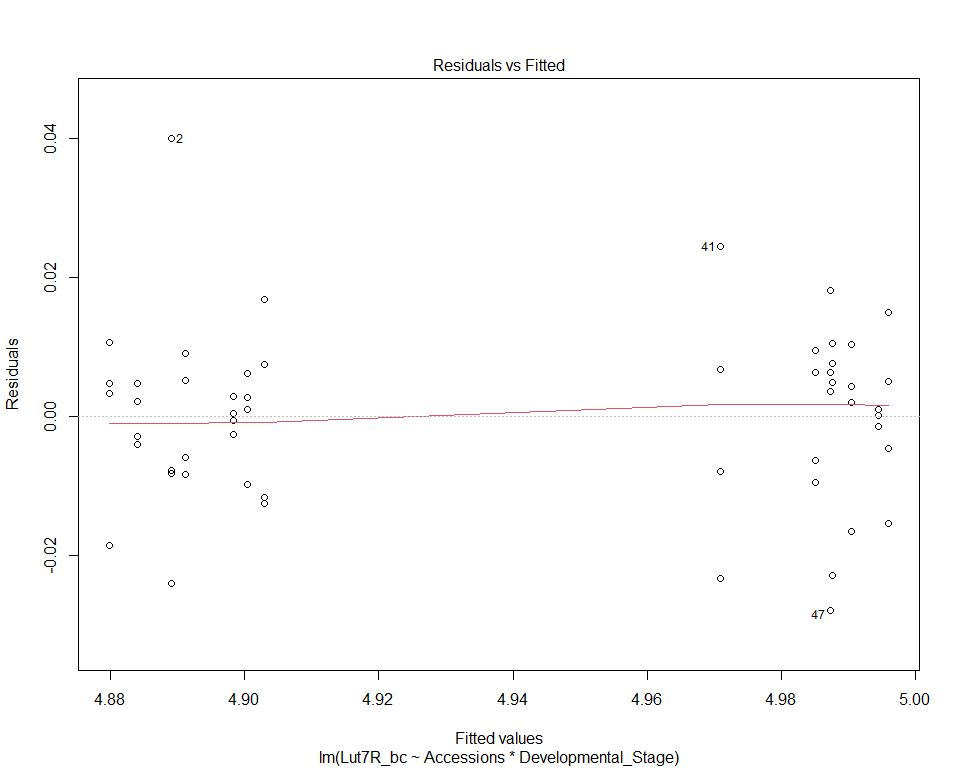


plot(Mod2_bc,2)


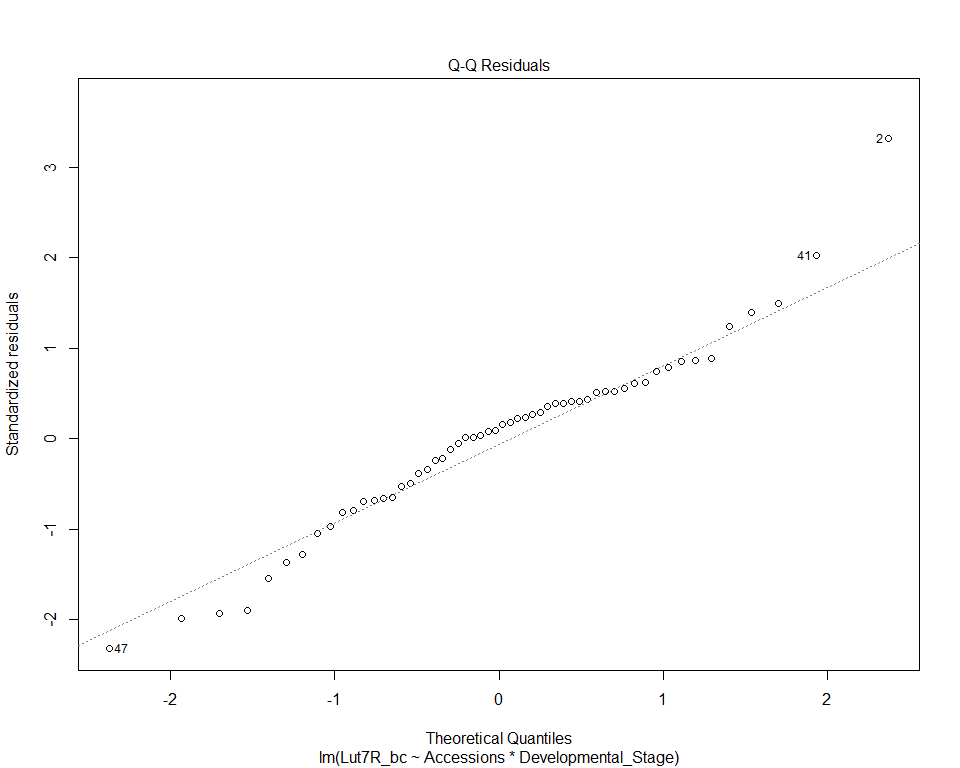


anova(Mod2_bc)

Analysis of Variance Table

Response: Lut7R_bc
 Df Sum Sq Mean Sq F value Pr(>F)
Accessions 1 0.126351 0.126351 652.6962 < 2e-16 ***
Developmental_Stage 6 0.002254 0.000376 1.9407 0.09643 .
Accessions:Developmental_Stage 6 0.001183 0.000197 1.0184 0.42662
Residuals 42 0.008130 0.000194
---
Signif. codes: 0 '***' 0.001 '**' 0.01 '*' 0.05 '.' 0.1 ' ' 1

## Two-way ANOVA without interaction

Mod2bis=lm(Lut7R ~Accessions+Developmental_Stage, data=donnees)

### Postulates verification

res=residuals(Mod2bis)
ks.test(res,"pnorm", 0, sd(res))

Exact one-sample Kolmogorov-Smirnov test

data: res
D = 0.13709, p-value = 0.222
alternative hypothesis: two-sided

plot(Mod2bis,1)


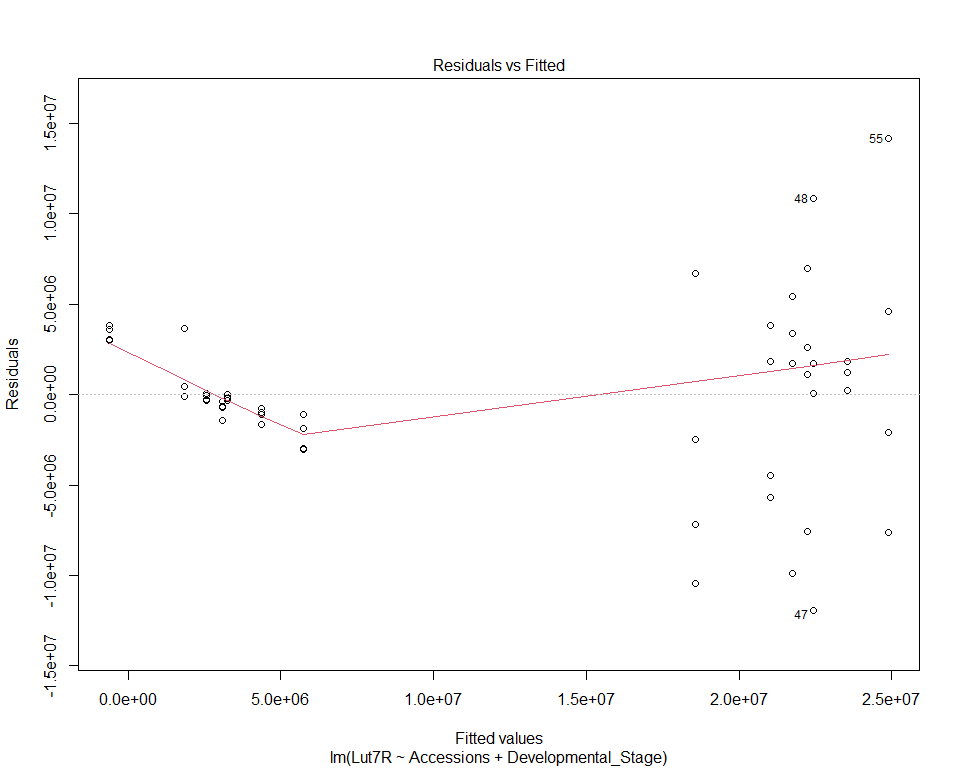


plot(Mod2bis,2)


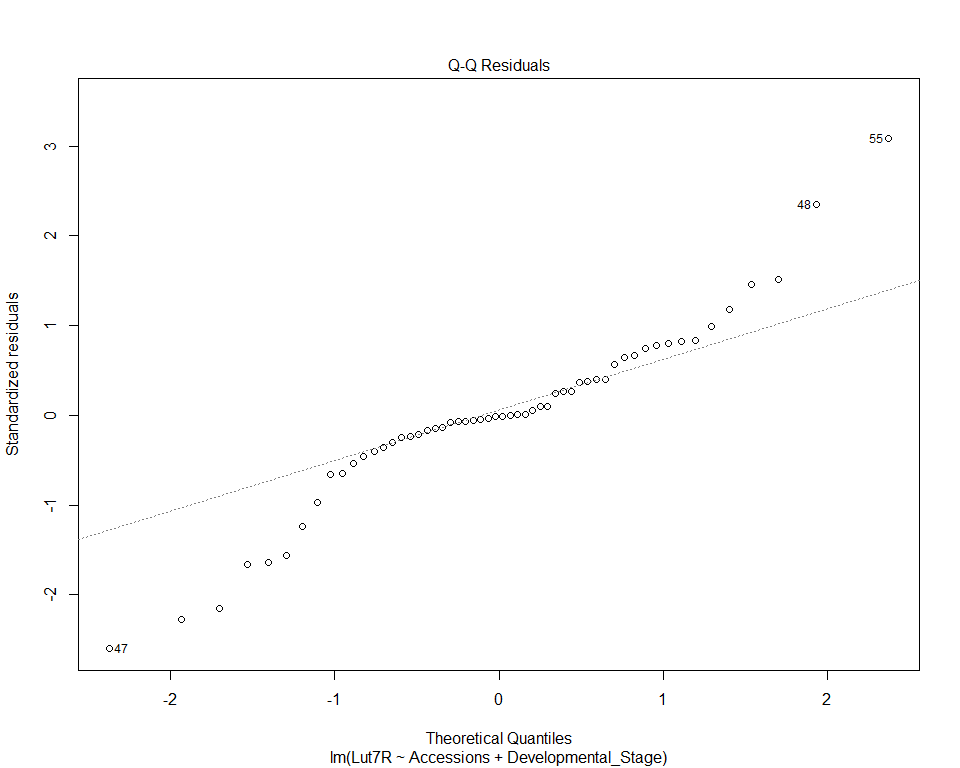


### Box-Cox transformation

summary(p2 <- powerTransform(Mod2bis))
mydata_bc <- transform(donnees, Lut7R_bc=bcPower(Lut7R,coef(p2)))

Mod2bis_bc <- lm(Lut7R_bc~ Accessions+Developmental_Stage, data=mydata_bc)
res=residuals(Mod2bis_bc)
ks.test(res,"pnorm", 0, sd(res))

Exact one-sample Kolmogorov-Smirnov test

data: res
D = 0.080884, p-value = 0.8284
alternative hypothesis: two-sided

plot(Mod2bis_bc,1)


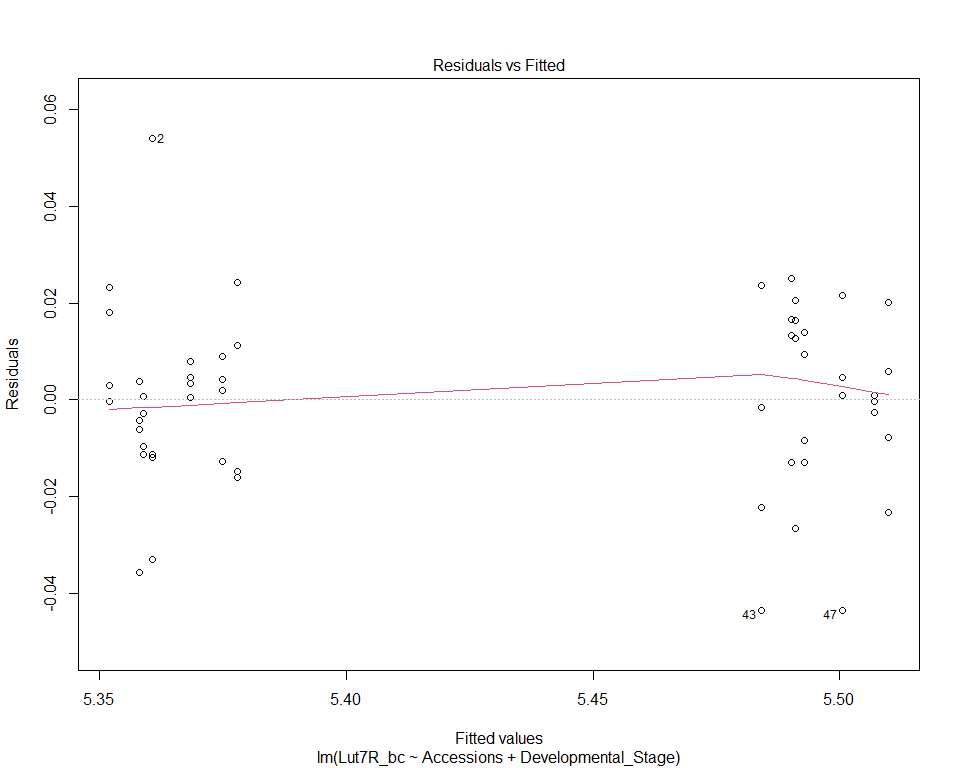


plot(Mod2bis_bc,2)


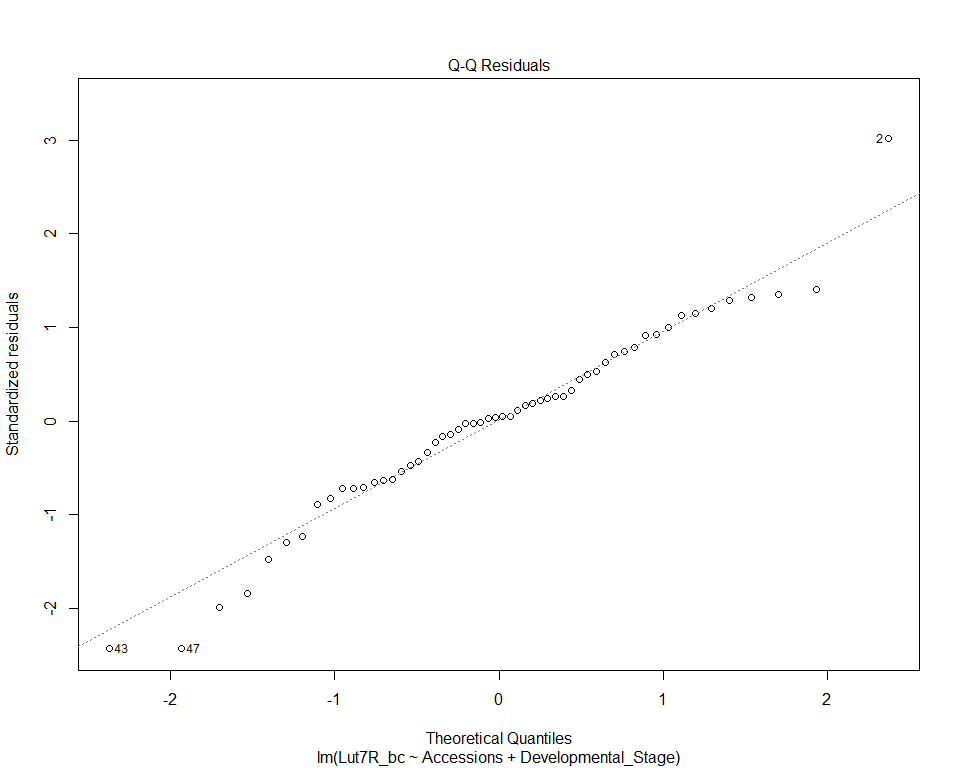


anova(Mod2bis_bc)

Analysis of Variance Table

Response: Lut7R_bc
 Df Sum Sq Mean Sq F value Pr(>F)
Accessions 1 0.243881 0.243881 650.0851 < 2e-16 ***
Developmental_Stage 6 0.004343 0.000724 1.9296 0.09508 .
Residuals 48 0.018007 0.000375
---
Signif. codes: 0 '***' 0.001 '**' 0.01 '*' 0.05 '.' 0.1 ' ' 1

### Models comparison

model_complet= aov(Lut7R_bc ~Accessions*Developmental_Stage, data=mydata_bc)
model_rduit=aov(Lut7R_bc ~Accessions+Developmental_Stage, data=mydata_bc)
anova(model_complet,model_rduit)

Analysis of Variance Table

Model 1: Lut7R_bc ~ Accessions * Developmental_Stage
Model 2: Lut7R_bc ~ Accessions + Developmental_Stage
 Res.Df RSS Df Sum of Sq F Pr(>F)
1 42 0.015744
2 48 0.018007 -6 -0.0022635 1.0064 0.4341

### Pairwise comparison

emm_Lut7R_1 <- emmeans(Mod2bis_bc, pairwise ~ Developmental_Stage |Accessions)
cld_Acc=cld(emm_Lut7R_1, Letter="abcdefghijk")
cld_df <- as.data.frame(cld_Acc)
cld_df_ordered <- cld_df %>%
 arrange(factor(Accessions, levels = levels(donnees$Accessions)),
 factor(Developmental_Stage, levels = levels(donnees$Developmental_Stage)))
cld_df_ordered

Developmental_Stage Accessions emmean SE df lower.CL upper.CL .group
 2 leaves H1 5.360750 0.007320733 48 5.346031 5.375470 a
 3 leaves H1 5.358198 0.007320733 48 5.343478 5.372917 a
 4 leaves H1 5.358952 0.007320733 48 5.344232 5.373671 a
 5 leaves H1 5.352106 0.007320733 48 5.337387 5.366825 a
 6 leaves H1 5.368483 0.007320733 48 5.353764 5.383202 a
 9 leaves H1 5.374995 0.007320733 48 5.360276 5.389715 a
 12 leaves H1 5.377869 0.007320733 48 5.363150 5.392588 a

2 leaves I2 5.492735 0.007320733 48 5.478016 5.507455 a
 3 leaves I2 5.490183 0.007320733 48 5.475463 5.504902 a
 4 leaves I2 5.490937 0.007320733 48 5.476217 5.505656 a
 5 leaves I2 5.484091 0.007320733 48 5.469372 5.498810 a
 6 leaves I2 5.500468 0.007320733 48 5.485749 5.515187 a
 9 leaves I2 5.506980 0.007320733 48 5.492261 5.521700 a
 12 leaves I2 5.509854 0.007320733 48 5.495135 5.524573 a

Confidence level used: 0.95
P value adjustment: tukey method for comparing a family of 7 estimates
significance level used: alpha = 0.05
NOTE: If two or more means share the same grouping symbol,
 then we cannot show them to be different.
 But we also did not show them to be the same.

# Chry7R

## Two-way ANOVA with interaction

Mod3=lm(Chry7R~Accessions*Developmental_Stage, data=donnees)

### Postulates verification

res3=residuals(Mod3)
ks.test(res3, "pnorm", 0, sd(res))

Exact one-sample Kolmogorov-Smirnov test

data: res3
D = 0.51786, p-value = 1.865e-14
alternative hypothesis: two-sided

plot(Mod3,1)


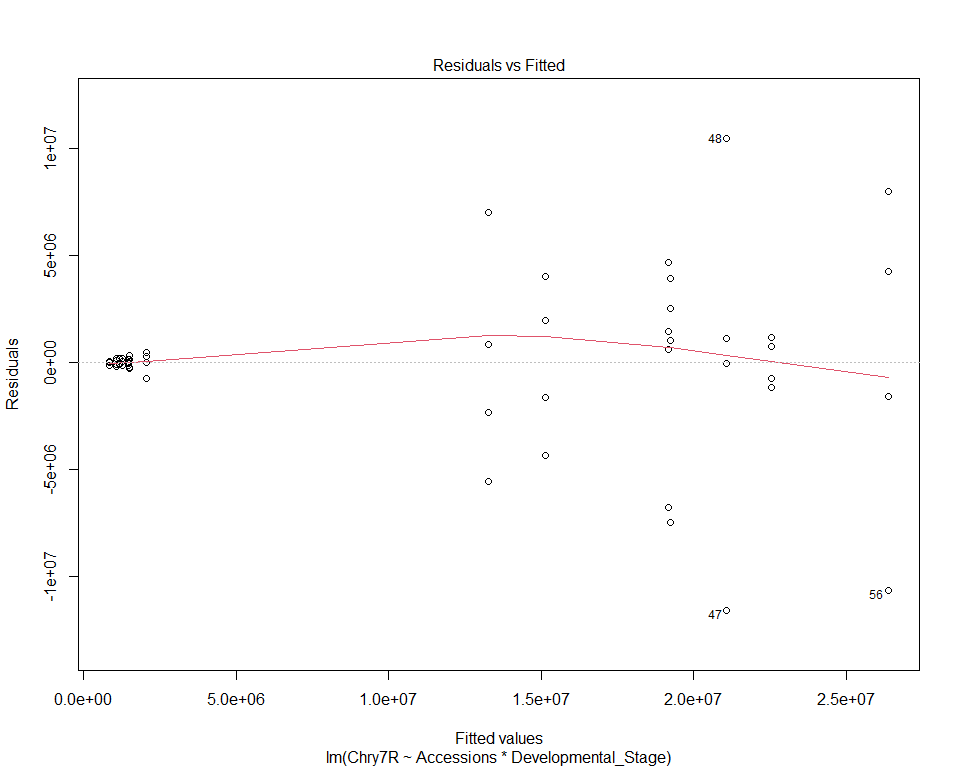


plot(Mod3,2)


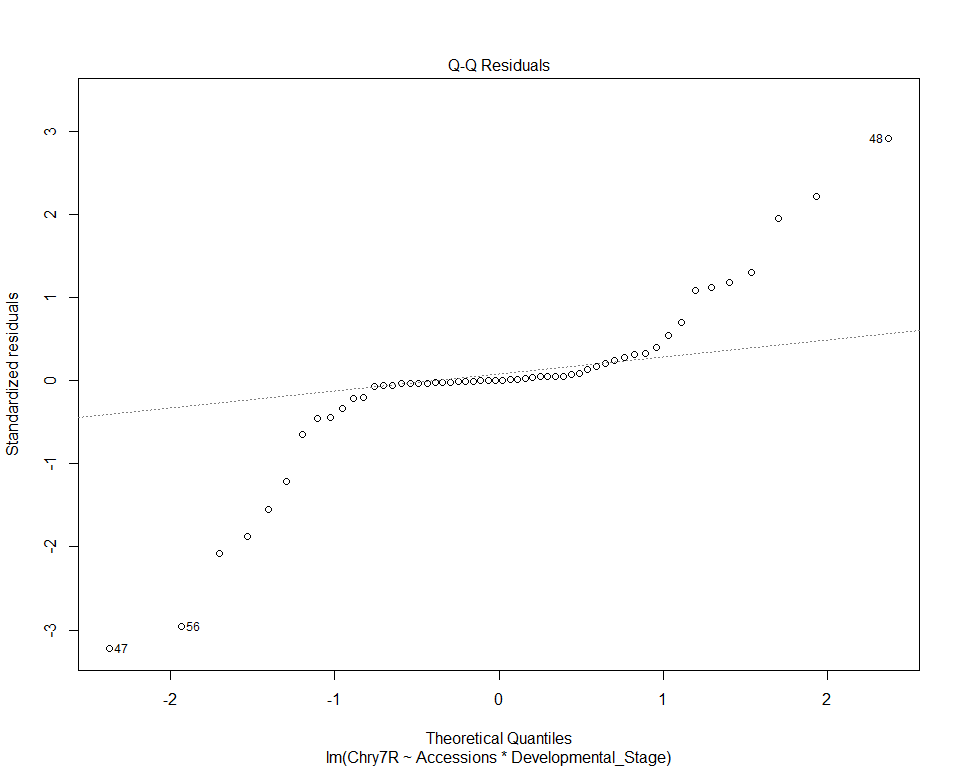


### Box-Cox transformation

summary(p3 <- powerTransform(Mod3))
mydata3_bc <- transform(donnees, Chry7R_bc=bcPower(Chry7R,coef(p3)))

Mod3_bc <- lm(Chry7R_bc~ Accessions*Developmental_Stage,data=mydata3_bc)
ks.test(residuals(Mod3_bc), "pnorm", 0, sd(residuals(Mod3_bc)))

Exact one-sample Kolmogorov-Smirnov test

data: residuals(Mod3_bc)
D = 0.10602, p-value = 0.5206
alternative hypothesis: two-sided

plot(Mod3_bc,1)


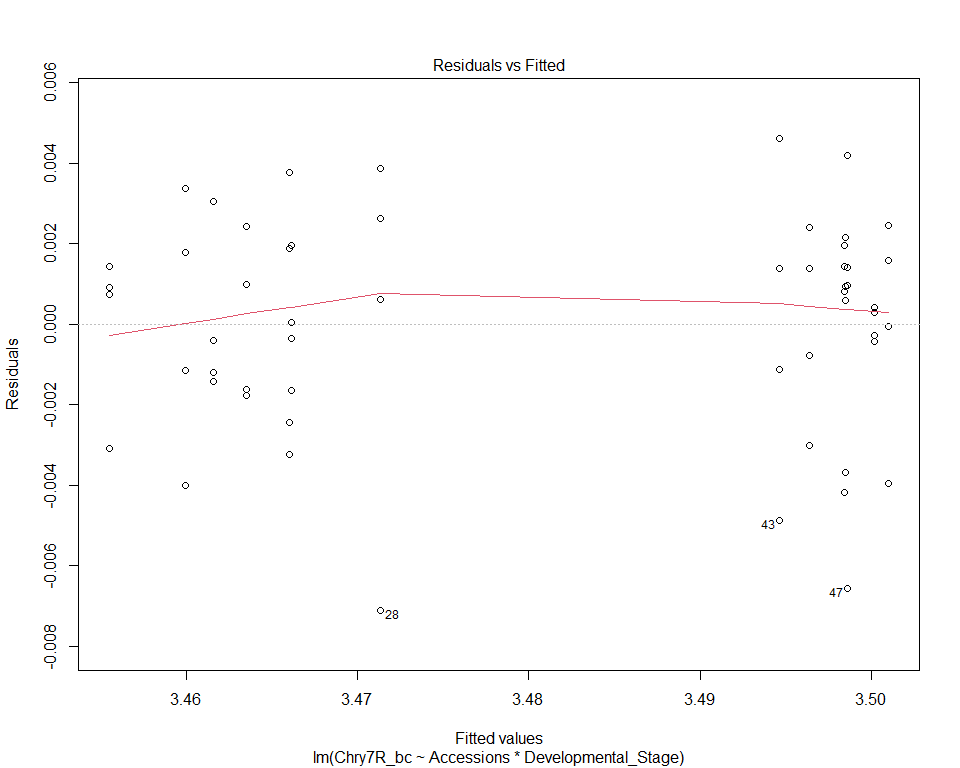


plot(Mod3_bc,2)


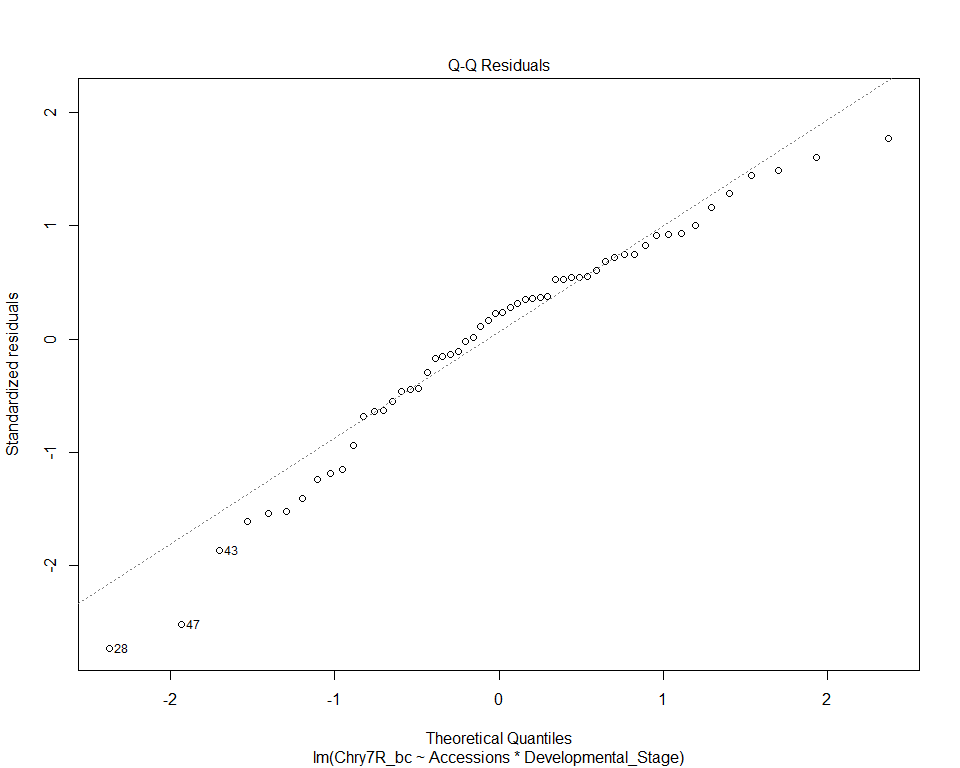


anova(Mod3_bc)

Analysis of Variance Table

Response: Chry7R_bc
 Df Sum Sq Mean Sq F value Pr(>F)
Accessions 1 0.0169644 0.0169644 1870.0087 < 2.2e-16 ***
Developmental_Stage 6 0.0005280 0.0000880 9.7008 1.07e-06 ***
Accessions:Developmental_Stage 6 0.0002007 0.0000334 3.6866 0.004949 **
Residuals 42 0.0003810 0.0000091
---
Signif. codes: 0 '***' 0.001 '**' 0.01 '*' 0.05 '.' 0.1 ' ' 1

### Pairwise comparison

emm_Chry7R_2 <- emmeans(Mod3_bc, pairwise ~ Developmental_Stage | Accessions)
cld2_chry=cld(emm_Chry7R_2, Letter="abcdefghijk")
cld2_df <- as.data.frame(cld2_chry)
cld2_df_ordered <- cld2_df %>%
 arrange(factor(Accessions, levels = levels(donnees$Accessions)),
 factor(Developmental_Stage, levels = levels(donnees$Developmental_Stage)))
cld2_df_ordered

Developmental_Stage Accessions emmean SE df lower.CL upper.CL .group
 2 leaves H1 3.455534 0.001505974 42 3.452495 3.458573 a
 3 leaves H1 3.459941 0.001505974 42 3.456902 3.462980 ab
 4 leaves H1 3.461575 0.001505974 42 3.458536 3.464614 ab
 5 leaves H1 3.463514 0.001505974 42 3.460474 3.466553 b
 6 leaves H1 3.466118 0.001505974 42 3.463079 3.469157 bc
 9 leaves H1 3.466046 0.001505974 42 3.463007 3.469086 bc
 12 leaves H1 3.471330 0.001505974 42 3.468290 3.474369 c

2 leaves I2 3.496395 0.001505974 42 3.493356 3.499434 a
 3 leaves I2 3.498436 0.001505974 42 3.495397 3.501475 a
 4 leaves I2 3.498477 0.001505974 42 3.495438 3.501516 a
 5 leaves I2 3.494654 0.001505974 42 3.491615 3.497693 a
 6 leaves I2 3.498622 0.001505974 42 3.495583 3.501662 a
 9 leaves I2 3.500151 0.001505974 42 3.497112 3.503190 a
 12 leaves I2 3.500993 0.001505974 42 3.497954 3.504033 a

Confidence level used: 0.95
P value adjustment: tukey method for comparing a family of 7 estimates
significance level used: alpha = 0.05
NOTE: If two or more means share the same grouping symbol,
 then we cannot show them to be different.
 But we also did not show them to be the same.
