# Supplementary material for: Flavonoid compounds as a way to identify sources of carrot resistance to Alternaria leaf blight
Source: Mol Breed. 2025 Jun 14;45(6):55. doi: 10.1007/s11032-025-01573-1 (PMC12167411; doi:10.1007/s11032-025-01573-1)
Supplement: Supplementary file 2 — Supplementary Material 2 [file 11032_2025_1573_MOESM2_ESM.docx]

**Three flavonoids biomarkers of Carrot resistance to Alternaria leaf blight: ACCUMULATION PATTERN AT DIFFERENT PHENOLOGICAL STAGES AND CONSISTENCY ACROSS DIVERSE GENETIC BACKGROUNDS**

**Molecular breeding**

Marie Louisa Ramaroson*^1^, Claude Emmanuel Koutouan*^1^, Angelina El Ghaziri^1^, Raymonde Baltenweck^2^, Patricia Claudel^2^, Philippe Hugueney^2^, Sébastien Huet^1^, Anita Suel^1^, Linda Voisine^1^, Mathilde Briard^1^, Jean Jacques Helesbeux^3^, Latifa Hamama^1^, Valérie le Clerc^1^, Emmanuel Geoffriau^1,§^

1 Institut Agro, Université d’Angers, INRAE, IRHS, SFR 4207 QUASAV, Angers, France

2 Université de Strasbourg, INRAE, SVQV UMR-A 1131, F-68000 Colmar, France

3 Université de Strasbourg, INRAE, SVQV UMR-A 1131, F-68000 Colmar, France

§ Correspondence: [emmanuel.geoffriau@institut-agro.fr](mailto:emmanuel.geoffriau@institut-agro.fr); Tel : +33-(0)2 41 22 54 31

* The first two authors contributed equally to the paper

Online Resource 2:

Estimation of the flavonoid contents in each accession relative to the weight of dry carrot leaves at 7 developmental stages in carrot genotypes H1 (susceptible to Alternaria leaf blight) and I2 (resistant). Quantification was based on calibration curves established with the Apigenin-7-O-Glucoside standard. DW: dry weight. Values delineate the mean of four biological replications ± standard deviation.

| Leaf stages | Accessions | Api7R  (µg/g DW) | Lut7R  (µg/g DW) | Chry7R  (µg/g DW) |
| --- | --- | --- | --- | --- |
| 2 Leaves | H1 | 43.88 ± 15.31 | 110.99 ± 63.68 | 31.91 ± 3.27 |
|  | I2 | 296.58 ± 95.07 | 588.93 ± 137.37 | 446.59 ± 102.01 |
| 3 Leaves | H1 | 33.09 ± 3.53 | 73.77 ± 9.99 | 34.47 ± 4.48 |
|  | I2 | 281.82 ± 118.25 | 688.53 ± 194.47 | 579.43 ± 187.28 |
| 4 Leaves | H1 | 74.48 ± 25.67 | 78.32 ± 7.35 | 37.26 ± 3.39 |
|  | I2 | 456.42 ± 229.1 | 687.22 ± 231.63 | 601.44 ± 170.75 |
| 5 Leaves | H1 | 84.32 ± 22.16 | 88.32 ± 11.16 | 40.89 ± 2.76 |
|  | I2 | 411.2 ± 311.89 | 431.18 ± 181.46 | 377.43 ± 126.93 |
| 6 Leaves | H1 | 87.33 ± 26.4 | 94.43 ± 8.96 | 44.88 ± 5.32 |
|  | I2 | 627.9 ± 278.22 | 672.65 ± 302.47 | 625.7 ± 284.26 |
| 9 Leaves | H1 | 108.61 ± 20.38 | 107.66 ± 10.93 | 49.02 ± 5.23 |
|  | I2 | 981.29 ± 198.15 | 786.13 ± 37.67 | 718.43 ± 37.87 |
| 12 Leaves | H1 | 155.86 ± 59.39 | 111.72 ± 22.47 | 66.21 ± 12.46 |
|  | I2 | 1433.77 ± 622.37 | 818.5 ± 335.43 | 791.91 ± 277.46 |
