# Supplementary material for: Flavonoid compounds as a way to identify sources of carrot resistance to Alternaria leaf blight
Source: Mol Breed. 2025 Jun 14;45(6):55. doi: 10.1007/s11032-025-01573-1 (PMC12167411; doi:10.1007/s11032-025-01573-1)

**Three flavonoids biomarkers of Carrot resistance to Alternaria leaf blight: ACCUMULATION PATTERN AT DIFFERENT PHENOLOGICAL STAGES AND CONSISTENCY ACROSS DIVERSE GENETIC BACKGROUNDS**

**Molecular breeding**

Marie Louisa Ramaroson*^1^, Claude Emmanuel Koutouan*^1^, Angelina El Ghaziri^1^, Raymonde Baltenweck^2^, Patricia Claudel^2^, Philippe Hugueney^2^, Sébastien Huet^1^, Anita Suel^1^, Linda Voisine^1^, Mathilde Briard^1^, Jean Jacques Helesbeux^3^, Latifa Hamama^1^, Valérie le Clerc^1^, Emmanuel Geoffriau^1,§^

1 Institut Agro, Université d’Angers, INRAE, IRHS, SFR 4207 QUASAV, Angers, France

2 Université de Strasbourg, INRAE, SVQV UMR-A 1131, F-68000 Colmar, France

3 Université de Strasbourg, INRAE, SVQV UMR-A 1131, F-68000 Colmar, France

§ Correspondence: [emmanuel.geoffriau@institut-agro.fr](mailto:emmanuel.geoffriau@institut-agro.fr); Tel : +33-(0)2 41 22 54 31

* The first two authors contributed equally to the paper

Online Resource 3: Correspondence analysis between disease severity and the three flavone contents in eight accessions

Table of contents

[1 ***Online Resource 3A****:* Api7R 1](#_Toc195730373)

[2 ***Online Resource 3B:*** Lut7R 3](#_Toc195730374)

[3 ***Online Resource 3C:*** Chry7R 4](#_Toc195730375)

## 1 ***Online Resource 3A****:* Api7R

*Factor map and output table of the correspondence analysis (CA) relating four classes of the Api7R content in blue (four quartiles) and 3 levels of disease score in brown, yellow, and green for high, medium and low ALB respectively.*

YDis_3C Api7R <25% Api7R [25-50] Api7R [50-75] Api7R >75%
 Low ALB 4 13 14 17
 medium ALB 5 7 5 7
 High ALB 15 4 5 0

Call:
CA(X = TAB_Api, graph = FALSE)

The chi square of independence between the two variables is equal to 28.83333 (p-value = 6.541775e-05 ).

Eigenvalues
 Dim.1 Dim.2
Variance 0.296 0.005
% of var. 98.425 1.575
Cumulative % of var. 98.425 100.000

Rows
 Iner*1000 Dim.1 ctr cos2 Dim.2 ctr cos2
Low ALB | 81.597 | 0.401 27.238 0.987 | 0.046 22.762 0.013 |
medium ALB | 6.944 | 0.117 1.168 0.497 | -0.118 73.832 0.503 |
High ALB | 211.806 | -0.920 71.594 0.999 | 0.025 3.406 0.001 |

Columns
 Iner*1000 Dim.1 ctr cos2 Dim.2 ctr cos2
Api7R <25% | 197.917 | -0.890 66.932 1.000 | -0.015 1.172 0.000 |
Api7R [25-50] | 9.549 | 0.181 2.764 0.856 | -0.074 29.133 0.144 |
Api7R [50-75] | 6.944 | 0.123 1.280 0.545 | 0.112 66.824 0.455 |
Api7R >75% | 85.938 | 0.586 29.025 0.998 | -0.023 2.872 0.002 |


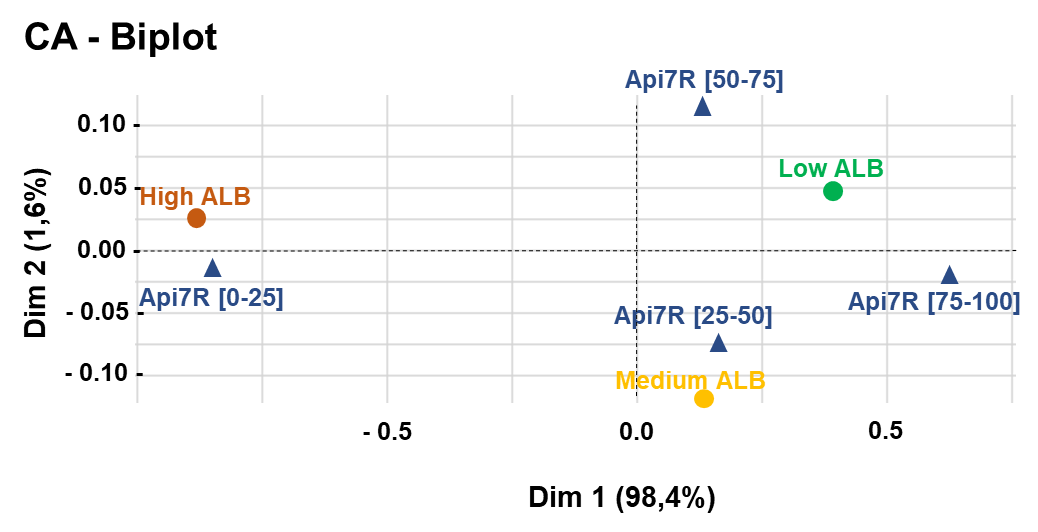


## 2 ***Online Resource 3B:*** Lut7R

*Factor map and output table of the correspondence analysis (CA) relating four classes of the Lut7R content in blue (four quartiles) and 3 levels of disease score in brown, yellow, and green for high, medium and low ALB respectively.*

YDis_3C Lut7R <25% Lut7R [25-50] Lut7R [50-75] Lut7R >75%
 Low ALB 2 10 19 17
 medium ALB 4 8 5 7
 High ALB 18 6 0 0

Call:
CA(X = TAB_Lut, graph = FALSE)

The chi square of independence between the two variables is equal to 52.5 (p-value = 1.479304e-09 ).

Eigenvalues
 Dim.1 Dim.2
Variance 0.529 0.018
% of var. 96.698 3.302
Cumulative % of var. 96.698 100.000

Rows
 Iner*1000 Dim.1 ctr cos2 Dim.2 ctr cos2
Low ALB | 154.514 | 0.549 28.484 0.975 | 0.088 21.516 0.025 |
medium ALB | 17.361 | 0.126 0.748 0.228 | -0.232 74.252 0.772 |
High ALB | 375.000 | -1.223 70.768 0.998 | 0.055 4.232 0.002 |

Columns
 Iner*1000 Dim.1 ctr cos2 Dim.2 ctr cos2
Lut7R <25% | 343.750 | -1.170 64.730 0.996 | 0.076 7.997 0.004 |
Lut7R [25-50] | 10.417 | -0.048 0.111 0.056 | -0.198 54.434 0.944 |
Lut7R [50-75] | 106.771 | 0.634 18.976 0.940 | 0.160 35.570 0.060 |
Lut7R >75% | 85.938 | 0.585 16.183 0.996 | -0.038 1.999 0.004 |


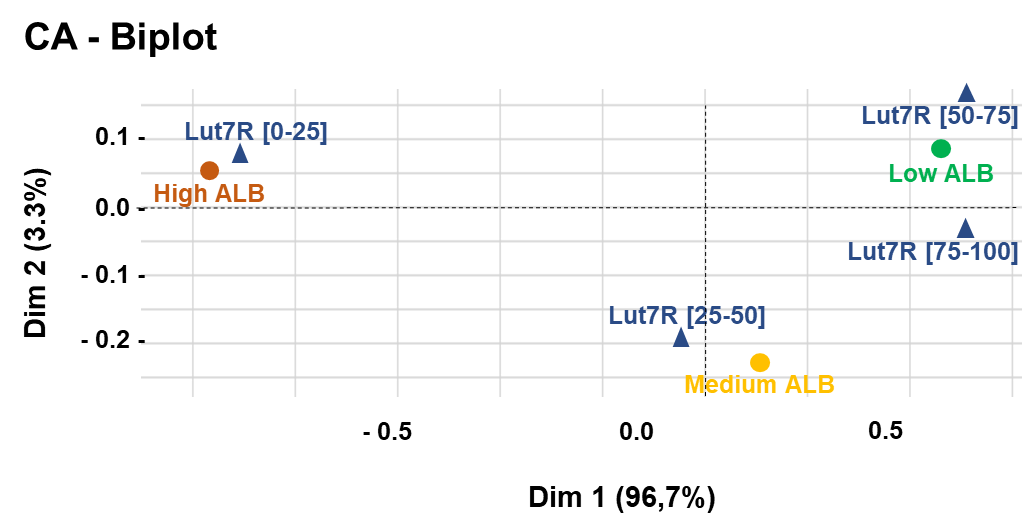


## 3 ***Online Resource 3C:*** Chry7R

*Factor map and output table of the correspondence analysis (CA) relating four classes of the Chry7R content in blue (four quartiles) and 3 levels of disease score in brown, yellow, and green for high, medium and low ALB respectively.*

YDis_3C Chry7R <25% Chry7R [25-50] Chry7R [50-75] Chry7R >75%
 Low ALB 4 8 15 21
 medium ALB 7 5 9 3
 High ALB 13 11 0 0

Call:
CA(X = TAB_Chry, graph = FALSE)

The chi square of independence between the two variables is equal to 41.83333 (p-value = 1.983487e-07 ).

Eigenvalues
 Dim.1 Dim.2
Variance 0.391 0.045
% of var. 89.677 10.323
Cumulative % of var. 89.677 100.000

Rows
 Iner*1000 Dim.1 ctr cos2 Dim.2 ctr cos2
Low ALB | 147.569 | 0.532 36.171 0.958 | -0.112 13.829 0.042 |
medium ALB | 34.722 | -0.067 0.285 0.032 | 0.367 74.715 0.968 |
High ALB | 253.472 | -0.997 63.544 0.980 | -0.144 11.456 0.020 |

Columns
 Iner*1000 Dim.1 ctr cos2 Dim.2 ctr cos2
Chry7R <25% | 142.361 | -0.753 36.271 0.996 | 0.050 1.384 0.004 |
Chry7R [25-50] | 59.028 | -0.469 14.099 0.933 | -0.125 8.741 0.067 |
Chry7R [50-75] | 85.938 | 0.492 15.457 0.703 | 0.320 56.765 0.297 |
Chry7R >75% | 148.438 | 0.731 34.173 0.900 | -0.244 33.110 0.100 |


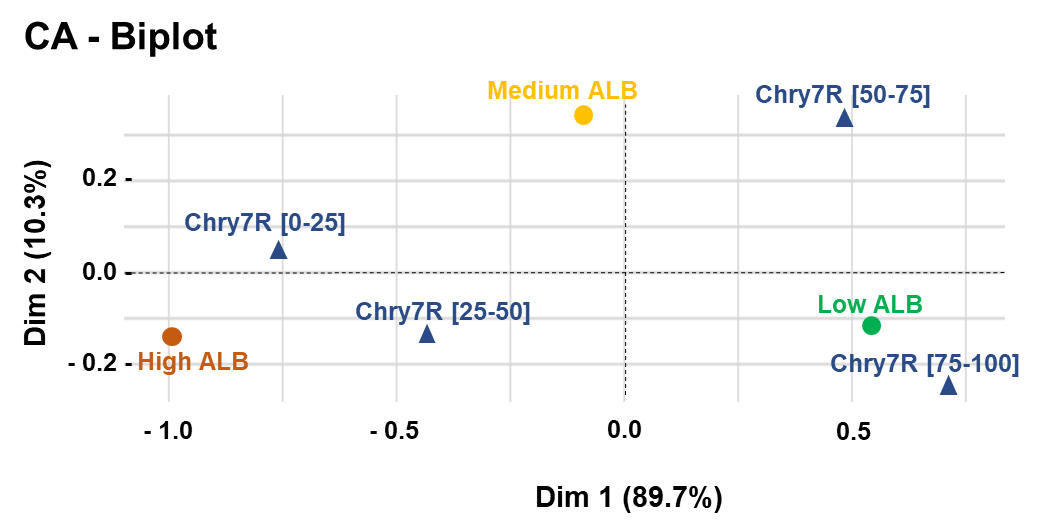

Supplement: Supplementary file 3 — Supplementary Material 3 [file 11032_2025_1573_MOESM3_ESM.docx]
