# Supplementary material for: Flavonoid compounds as a way to identify sources of carrot resistance to Alternaria leaf blight
Source: Mol Breed. 2025 Jun 14;45(6):55. doi: 10.1007/s11032-025-01573-1 (PMC12167411; doi:10.1007/s11032-025-01573-1)
Supplement: Supplementary file 4 — Supplementary Material 4 [file 11032_2025_1573_MOESM4_ESM.docx]

**Three flavonoids biomarkers of Carrot resistance to Alternaria leaf blight: ACCUMULATION PATTERN AT DIFFERENT PHENOLOGICAL STAGES AND CONSISTENCY ACROSS DIVERSE GENETIC BACKGROUNDS**

**Molecular breeding**

Marie Louisa Ramaroson*^1^, Claude Emmanuel Koutouan*^1^, Angelina El Ghaziri^1^, Raymonde Baltenweck^2^, Patricia Claudel^2^, Philippe Hugueney^2^, Sébastien Huet^1^, Anita Suel^1^, Linda Voisine^1^, Mathilde Briard^1^, Jean Jacques Helesbeux^3^, Latifa Hamama^1^, Valérie le Clerc^1^, Emmanuel Geoffriau^1,§^

1 Institut Agro, Université d’Angers, INRAE, IRHS, SFR 4207 QUASAV, Angers, France

2 Université de Strasbourg, INRAE, SVQV UMR-A 1131, F-68000 Colmar, France

3 Université de Strasbourg, INRAE, SVQV UMR-A 1131, F-68000 Colmar, France

§ Correspondence: [emmanuel.geoffriau@institut-agro.fr](mailto:emmanuel.geoffriau@institut-agro.fr); Tel : +33-(0)2 41 22 54 31

* The first two authors contributed equally to the paper

Online Resource 4: Disease data analysis (Trial 2)

Table of contents

Trial2_DS <- read_excel("données ALB et metabo.xlsx",sheet = "données ALB")

The following is a summary of the dataset of trial 2.

summary(Trial2_DS, maxsum=8)

Accessions repetition Score
 PRESTO :4 1:7 Min. :3.000
 H1 :3 2:8 1st Qu.:4.000
 A92 :4 3:8 Median :5.000
 B18 :4 4:8 Mean :5.452
 BOLERO :4 3rd Qu.:6.000
 BRILLYANCE:4 Max. :9.000
 VALOR :4
 I2 :4

## Linear mixed model

model=lmer(Score~Accessions+(1|repetition), data=Trial2_DS)

### Postulates verification

res=residuals(model)
shapiro.test(res)

Shapiro-Wilk normality test

data: res
W = 0.96432, p-value = 0.3777

plot(model)


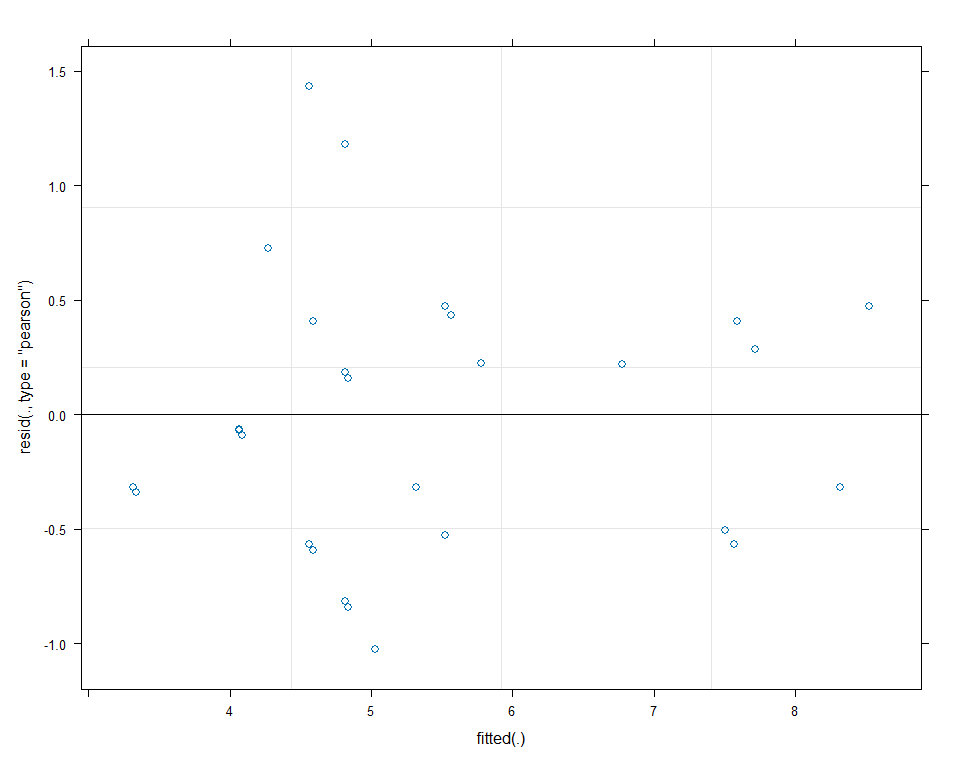


### Effects of Accessions and repetitions

Anova(model, test="F")

Analysis of Deviance Table (Type II Wald F tests with Kenward-Roger df)

Response: Score
 F Df Df.res Pr(>F)
Accessions 15.339 7 20.033 8.676e-07 ***
---
Signif. codes: 0 '***' 0.001 '**' 0.01 '*' 0.05 '.' 0.1 ' ' 1

ranova(model)

ANOVA-like table for random-effects: Single term deletions

Model:
Score ~ Accessions + (1 | repetition)
 npar logLik AIC LRT Df Pr(>Chisq)
<none> 10 -32.274 84.548
(1 | repetition) 9 -35.165 88.329 5.7809 1 0.0162 *
---
Signif. codes: 0 '***' 0.001 '**' 0.01 '*' 0.05 '.' 0.1 ' ' 1

### Pairwise comparisons

em_acc=emmeans(model, pairwise ~ Accessions, adjust = "tukey")
cld_results <- cld(em_acc, Letters = letters, adjust = "tukey")

Note: adjust = "tukey" was changed to "sidak"
because "tukey" is only appropriate for one set of pairwise comparisons

cld_results

Accessions emmean SE df lower.CL upper.CL .group
 BRILLYANCE 3.75 0.441 11.7 2.29 5.21 a
 BOLERO 4.50 0.441 11.7 3.04 5.96 a
 A92 5.00 0.441 11.7 3.54 6.46 a
 I2 5.00 0.441 11.7 3.54 6.46 a
 VALOR 5.25 0.441 11.7 3.79 6.71 a
 B18 5.25 0.441 11.7 3.79 6.71 a
 H1 7.19 0.491 14.9 5.63 8.75 b
 PRESTO 8.00 0.441 11.7 6.54 9.46 b

Degrees-of-freedom method: kenward-roger
Confidence level used: 0.95
Conf-level adjustment: sidak method for 8 estimates
P value adjustment: tukey method for comparing a family of 8 estimates
significance level used: alpha = 0.05
NOTE: If two or more means share the same grouping symbol,
 then we cannot show them to be different.
 But we also did not show them to be the same.
