# Supplementary material for: Flavonoid compounds as a way to identify sources of carrot resistance to Alternaria leaf blight
Source: Mol Breed. 2025 Jun 14;45(6):55. doi: 10.1007/s11032-025-01573-1 (PMC12167411; doi:10.1007/s11032-025-01573-1)
Supplement: Supplementary file 5 — Supplementary Material 5 [file 11032_2025_1573_MOESM5_ESM.docx]

**Three flavonoids biomarkers of Carrot resistance to Alternaria leaf blight: ACCUMULATION PATTERN AT DIFFERENT PHENOLOGICAL STAGES AND CONSISTENCY ACROSS DIVERSE GENETIC BACKGROUNDS**

**Molecular breeding**

Marie Louisa Ramaroson*^1^, Claude Emmanuel Koutouan*^1^, Angelina El Ghaziri^1^, Raymonde Baltenweck^2^, Patricia Claudel^2^, Philippe Hugueney^2^, Sébastien Huet^1^, Anita Suel^1^, Linda Voisine^1^, Mathilde Briard^1^, Jean Jacques Helesbeux^3^, Latifa Hamama^1^, Valérie le Clerc^1^, Emmanuel Geoffriau^1,§^

1 Institut Agro, Université d’Angers, INRAE, IRHS, SFR 4207 QUASAV, Angers, France

2 Université de Strasbourg, INRAE, SVQV UMR-A 1131, F-68000 Colmar, France

3 Université de Strasbourg, INRAE, SVQV UMR-A 1131, F-68000 Colmar, France

§ Correspondence: [emmanuel.geoffriau@institut-agro.fr](mailto:emmanuel.geoffriau@institut-agro.fr); Tel : +33-(0)2 41 22 54 31

* The first two authors contributed equally to the paper

Online Resource 5: metabolic data from Trial 2 analyses

Table of contents

- Api7R page 2
- Lut 7R page 9
- Chry7Rpage 15

One repetition of accession by developmental stage in each repetition (block). We start with an additive model with the three factors (Accession, Developmental Stage, and Block). When the Block factor is not significant, we study the interaction. A step-by-step analysis of these data is shown.

# Api7R

## Api7R Model 1

modelApi1=lm(Api7R~Accessions+Developmental_stage+ repetition, data= sup6)
res=residuals(modelApi1)
ks.test(res,"pnorm", 0, sd(res))

Exact one-sample Kolmogorov-Smirnov test

data: res
D = 0.13887, p-value = 0.0446
alternative hypothesis: two-sided

plot(modelApi1,1)


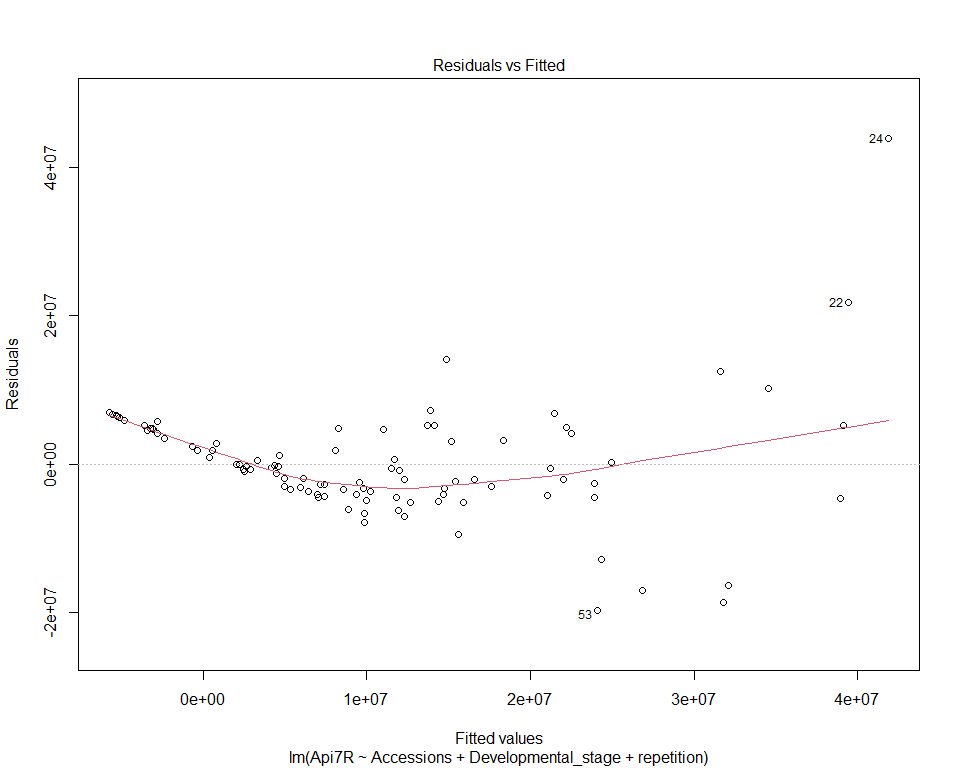


plot(modelApi1,2)


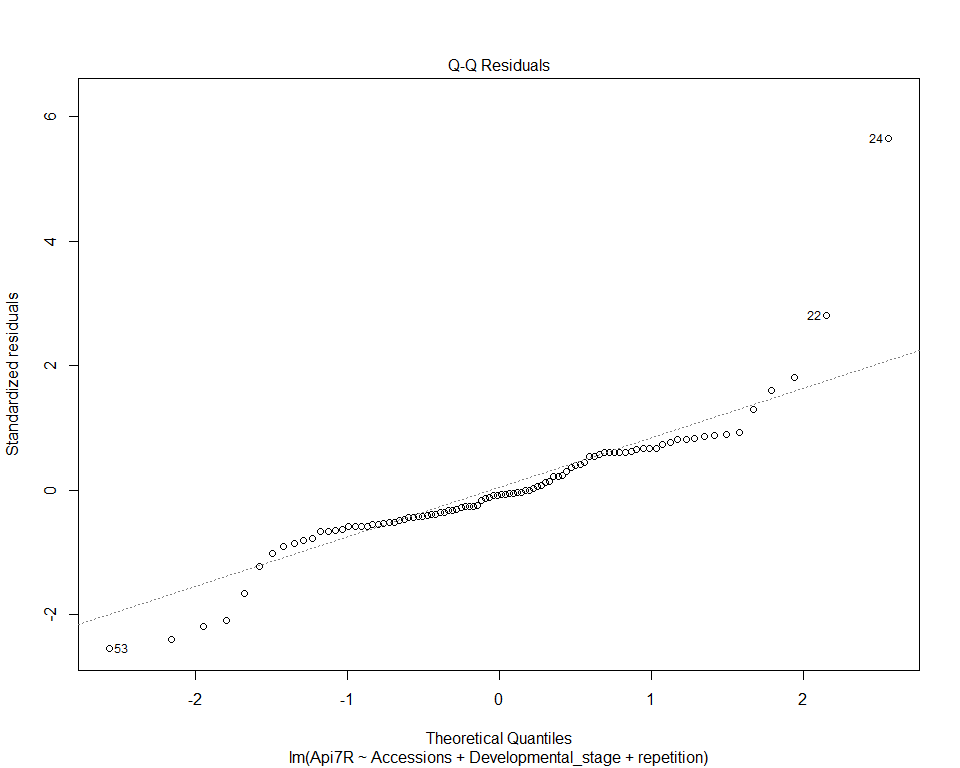


### Box-Cox transformation

summary(p2 <- powerTransform(modelApi1))
sup6_bc <- transform(sup6, Api7R1_bc=bcPower(Api7R,coef(p2)))

modelApi1_bc <- lm(Api7R1_bc~ Accessions+Developmental_stage+ repetition, data=sup6_bc)
ks.test(residuals(modelApi1_bc), "pnorm", 0, sd(residuals(modelApi1_bc)))

Exact one-sample Kolmogorov-Smirnov test
data: residuals(modelApi1_bc)
D = 0.064093, p-value = 0.8012
alternative hypothesis: two-sided

plot(modelApi1_bc,1)


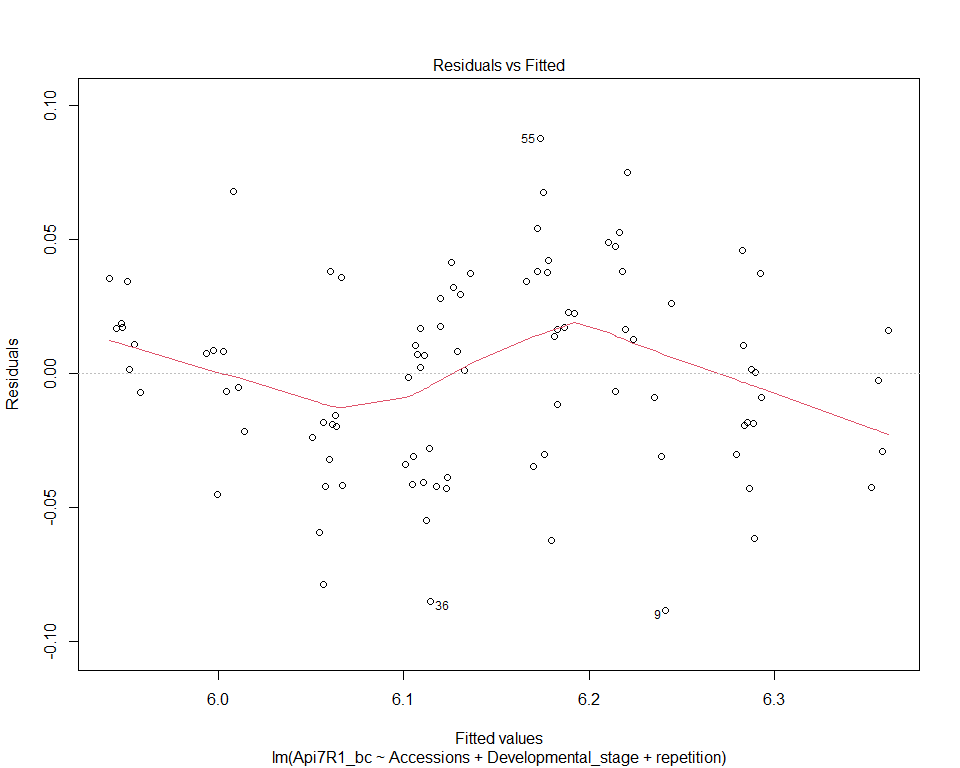


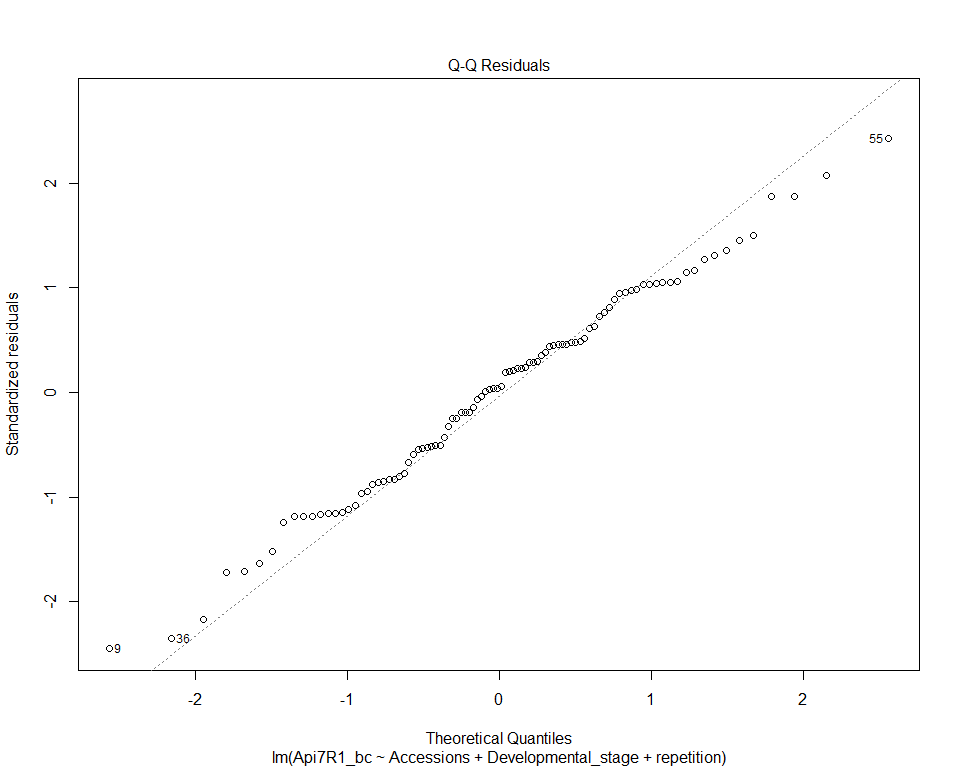
plot(modelApi1_bc,2)

anova(modelApi1)

Analysis of Variance Table

Response: Api7R
 Df Sum Sq Mean Sq F value Pr(>F)
Accessions 7 8.2984e+15 1.1855e+15 16.9163 1.075e-13 ***
Developmental_stage 2 3.6293e+15 1.8147e+15 25.8944 1.824e-09 ***
repetition 3 1.3449e+14 4.4829e+13 0.6397 0.5916
Residuals 83 5.8166e+15 7.0080e+13
---
Signif. codes: 0 '***' 0.001 '**' 0.01 '*' 0.05 '.' 0.1 ' ' 1

## Api7R model 2

modelApi=lm(Api7R~Accessions*Developmental_stage, data= sup6)
res=residuals(modelApi)

### Postulates verification

ks.test(res, "pnorm", 0, sd(res))

Exact one-sample Kolmogorov-Smirnov test

data: res
D = 0.23501, p-value = 3.795e-05
alternative hypothesis: two-sided

plot(modelApi,1)


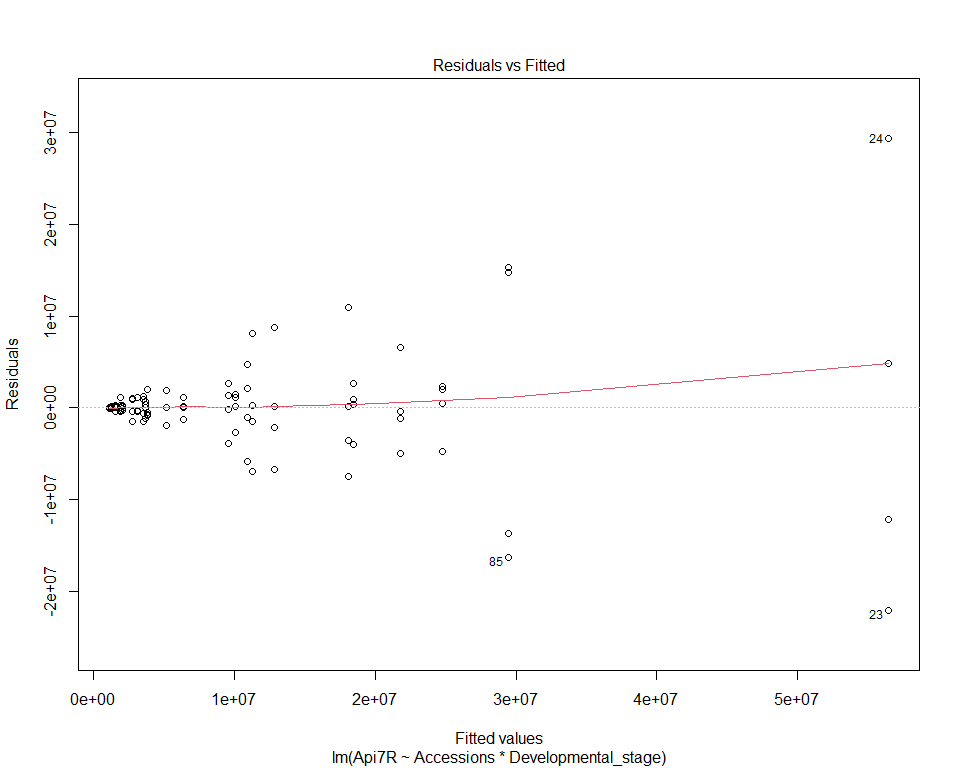


plot(modelApi,2)


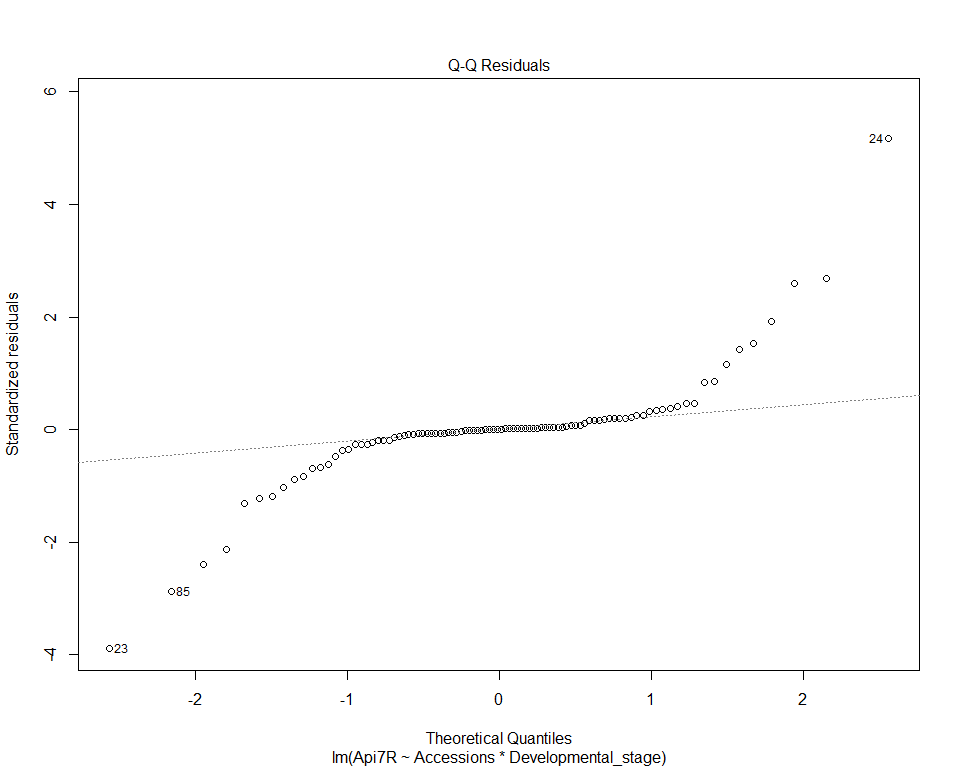


### Box-cox transformation

summary(p2 <- powerTransform(modelApi))
sup6_bc <- transform(sup6, Api7R_bc=bcPower(Api7R,coef(p2)))

modelApi_bc <- lm(Api7R_bc~ Accessions*Developmental_stage, data=sup6_bc)
ks.test(residuals(modelApi_bc), "pnorm", 0, sd(residuals(modelApi_bc)))

Exact one-sample Kolmogorov-Smirnov test
data: residuals(modelApi_bc)
D = 0.090131, p-value = 0.3932
alternative hypothesis: two-sided

plot(modelApi_bc,1)


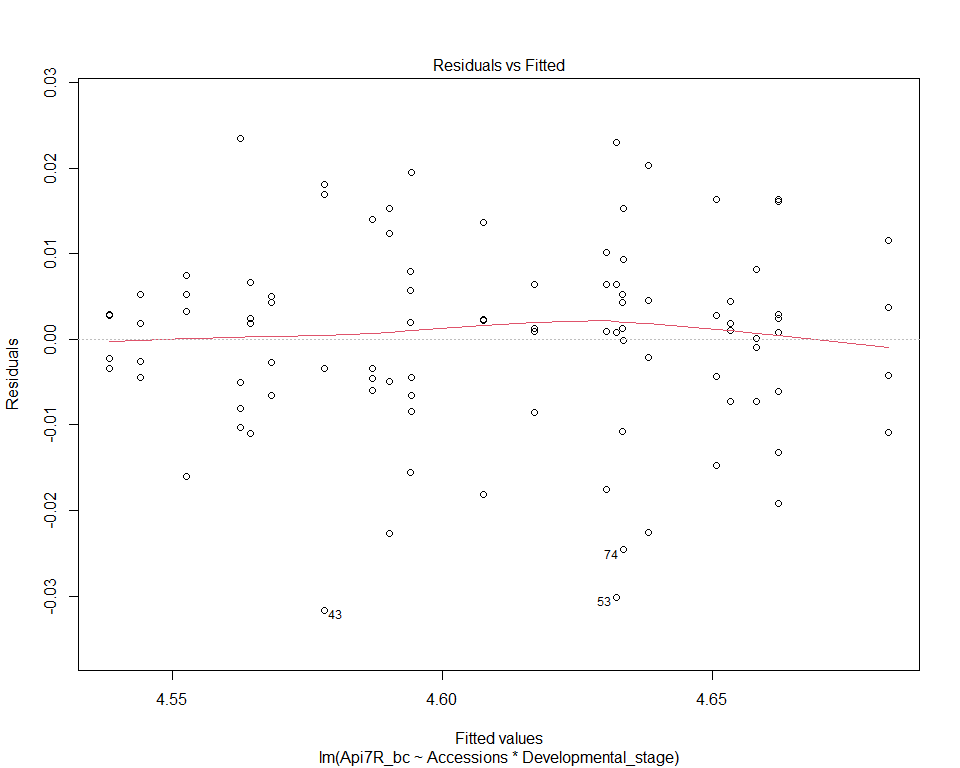


plot(modelApi_bc,2)


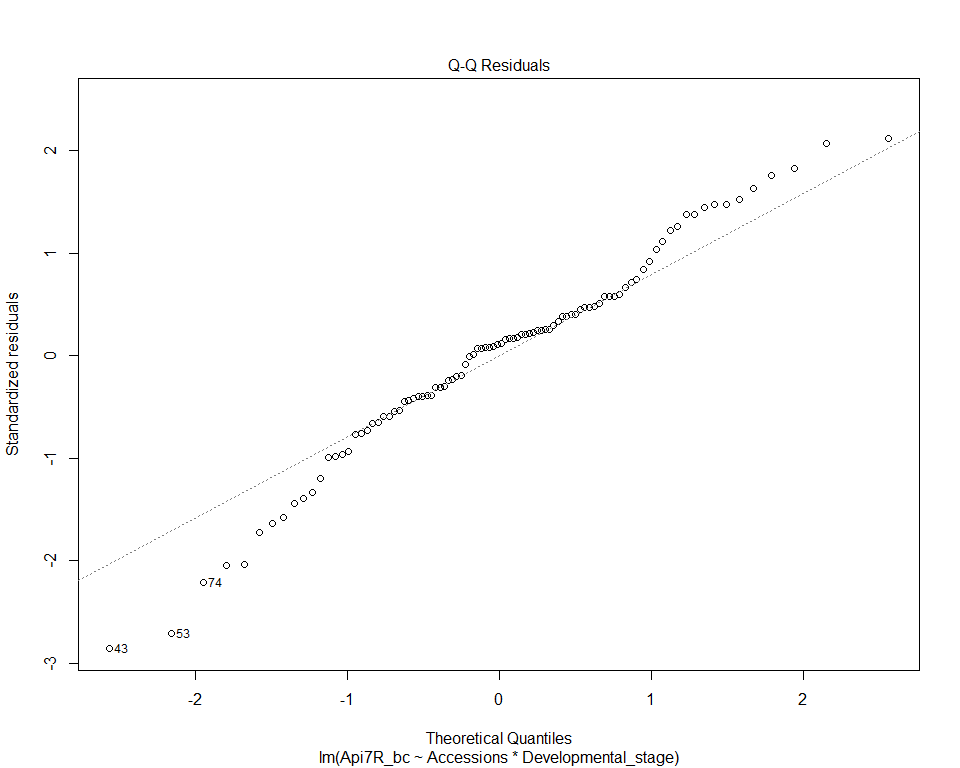


### ANOVA

anova(modelApi_bc)

Analysis of Variance Table

Response: Api7R_bc
 Df Sum Sq Mean Sq F value Pr(>F)
Accessions 7 0.080917 0.011560 70.3163 < 2.2e-16 ***
Developmental_stage 2 0.075378 0.037689 229.2610 < 2.2e-16 ***
Accessions:Developmental_stage 14 0.006479 0.000463 2.8151 0.002124 **
Residuals 72 0.011836 0.000164
---
Signif. codes: 0 '***' 0.001 '**' 0.01 '*' 0.05 '.' 0.1 ' ' 1

##

## Pairwise comparison

em_acc=emmeans(modelApi_bc, ~ Accessions|Developmental_stage, adjust = "tukey")
cld_results <- cld(em_acc, Letters = letters, adjust = "tukey")
cld_results

Developmental_stage = 2 Leaves:
 Accessions emmean SE df lower.CL upper.CL .group
 PRESTO 4.538 0.006411 72 4.520 4.556 a
 H1 4.544 0.006411 72 4.526 4.562 a
 VALOR 4.553 0.006411 72 4.535 4.571 ab
 BRILLYANCE 4.563 0.006411 72 4.545 4.581 abc
 BOLERO 4.578 0.006411 72 4.560 4.596 bc
 A92 4.587 0.006411 72 4.569 4.605 c
 B18 4.590 0.006411 72 4.572 4.608 c
 I2 4.632 0.006411 72 4.614 4.650 d

Developmental_stage = 6 Leaves:
 Accessions emmean SE df lower.CL upper.CL .group
 H1 4.564 0.006411 72 4.546 4.582 a
 PRESTO 4.568 0.006411 72 4.550 4.586 ab
 VALOR 4.594 0.006411 72 4.576 4.612 b
 BRILLYANCE 4.594 0.006411 72 4.576 4.612 b
 BOLERO 4.633 0.006411 72 4.615 4.651 c
 B18 4.651 0.006411 72 4.633 4.669 cd
 A92 4.653 0.006411 72 4.635 4.671 cd
 I2 4.662 0.006411 72 4.644 4.680 d

Developmental_stage = 10 Leaves:
 Accessions emmean SE df lower.CL upper.CL .group
 H1 4.607 0.006411 72 4.589 4.625 a
 PRESTO 4.617 0.006411 72 4.599 4.635 ab
 VALOR 4.630 0.006411 72 4.612 4.648 abc
 BRILLYANCE 4.633 0.006411 72 4.615 4.651 abc
 BOLERO 4.638 0.006411 72 4.620 4.656 bcd
 A92 4.658 0.006411 72 4.640 4.676 cde
 B18 4.662 0.006411 72 4.644 4.680 de
 I2 4.683 0.006411 72 4.665 4.701 e

Confidence level used: 0.95
Conf-level adjustment: sidak method for 8 estimates
P value adjustment: tukey method for comparing a family of 8 estimates
significance level used: alpha = 0.05
NOTE: If two or more means share the same grouping symbol,
 then we cannot show them to be different.
 But we also did not show them to be the same.

em_dvl=emmeans(modelApi_bc, ~ Developmental_stage|Accessions, adjust = "tukey")
cld_results2 <- cld(em_dvl, Letters = letters, adjust = "tukey")
cld_df <- as.data.frame(cld_results2)
cld_df_ordered <- cld_df %>%
 arrange(factor(Accessions, levels = levels(sup6$Accessions)),
 factor(Developmental_stage, levels = levels(sup6$Developmental_stage)))
cld_df_ordered

Accessions = PRESTO:
 Developmental_stage emmean SE df lower.CL upper.CL .group
 2 Leaves 4.538334 0.006410812 72 4.522662 4.554005 a
 6 Leaves 4.568204 0.006410812 72 4.552532 4.583875 b
 10 Leaves 4.616960 0.006410812 72 4.601288 4.632631 c

Accessions = H1:
 Developmental_stage emmean SE df lower.CL upper.CL .group
 2 Leaves 4.544032 0.006410812 72 4.528360 4.559704 a
 6 Leaves 4.564325 0.006410812 72 4.548654 4.579997 a
 10 Leaves 4.607452 0.006410812 72 4.591781 4.623124 b

Accessions = A92:
 Developmental_stage emmean SE df lower.CL upper.CL .group
 2 Leaves 4.586898 0.006410812 72 4.571226 4.602569 a
 6 Leaves 4.653305 0.006410812 72 4.637634 4.668977 b
 10 Leaves 4.658046 0.006410812 72 4.642375 4.673718 b

Accessions = B18:
 Developmental_stage emmean SE df lower.CL upper.CL .group
 2 Leaves 4.590030 0.006410812 72 4.574359 4.605702 a
 6 Leaves 4.650585 0.006410812 72 4.634913 4.666256 b
 10 Leaves 4.662155 0.006410812 72 4.646484 4.677827 b

Accessions = BOLERO:
 Developmental_stage emmean SE df lower.CL upper.CL .group
 2 Leaves 4.577990 0.006410812 72 4.562318 4.593662 a
 6 Leaves 4.633352 0.006410812 72 4.617680 4.649023 b
 10 Leaves 4.638152 0.006410812 72 4.622481 4.653824 b

Accessions = BRILLYANCE:
 Developmental_stage emmean SE df lower.CL upper.CL .group
 2 Leaves 4.562572 0.006410812 72 4.546900 4.578243 a
 6 Leaves 4.594162 0.006410812 72 4.578490 4.609833 b
 10 Leaves 4.633198 0.006410812 72 4.617527 4.648870 c

Accessions = VALOR:
 Developmental_stage emmean SE df lower.CL upper.CL .group
 2 Leaves 4.552548 0.006410812 72 4.536877 4.568220 a
 6 Leaves 4.593921 0.006410812 72 4.578249 4.609592 b
 10 Leaves 4.630336 0.006410812 72 4.614664 4.646007 c

Accessions = I2:
 Developmental_stage emmean SE df lower.CL upper.CL .group
 2 Leaves 4.632181 0.006410812 72 4.616510 4.647853 a
 6 Leaves 4.662080 0.006410812 72 4.646409 4.677752 b
 10 Leaves 4.682511 0.006410812 72 4.666840 4.698183 b

Confidence level used: 0.95
Conf-level adjustment: sidak method for 3 estimates
P value adjustment: tukey method for comparing a family of 3 estimates
significance level used: alpha = 0.05
NOTE: If two or more means share the same grouping symbol,
 then we cannot show them to be different.
 But we also did not show them to be the same.

# Lut7R

## Lut7R Model 1

modelLut1=lm(Lut7R~Accessions+Developmental_stage+ repetition, data= sup6)
res=residuals(modelLut1)
ks.test(res,"pnorm", 0, sd(res))

Exact one-sample Kolmogorov-Smirnov test

data: res
D = 0.12374, p-value = 0.09702
alternative hypothesis: two-sided

plot(modelLut1,1)


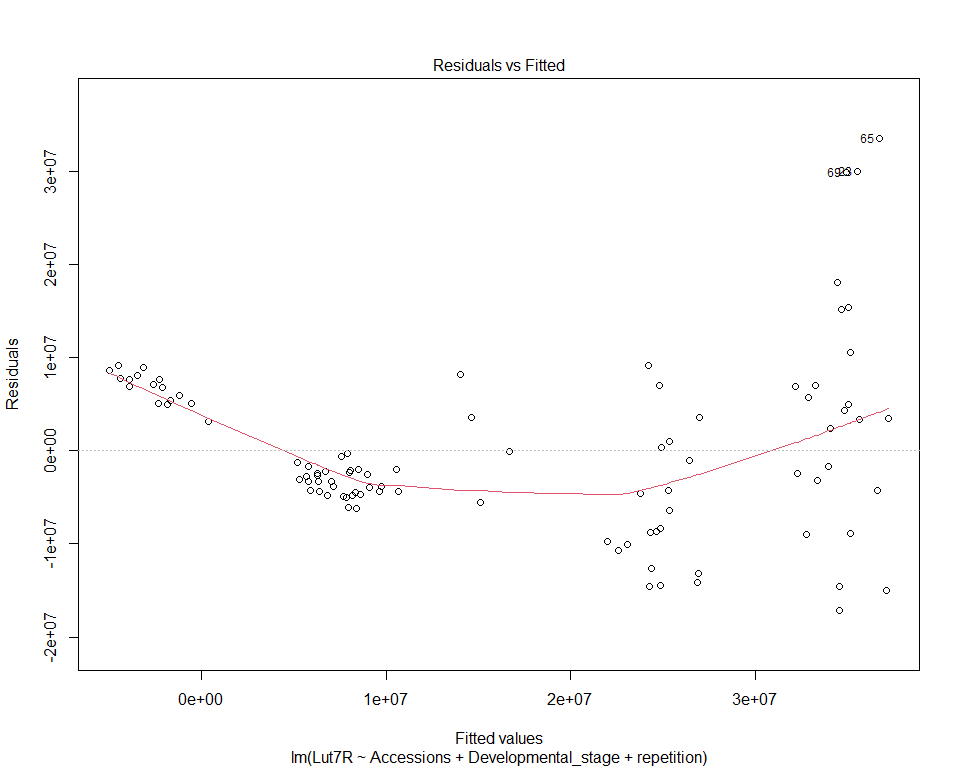


plot(modelLut1,2)


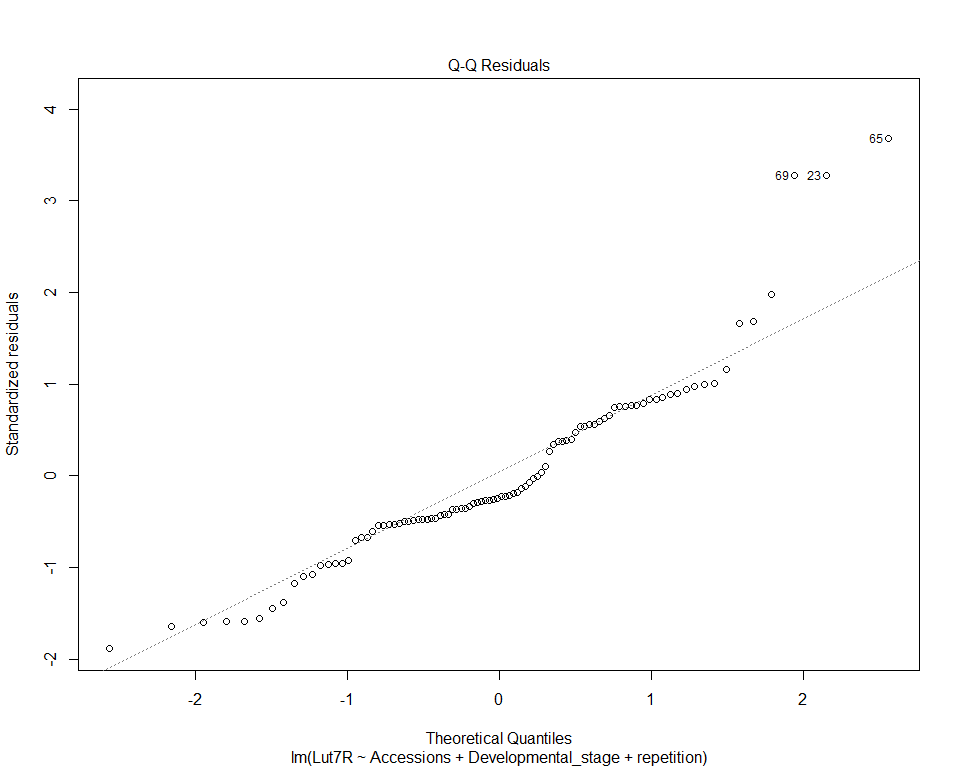


anova(modelLut1)

Analysis of Variance Table

Response: Lut7R
 Df Sum Sq Mean Sq F value Pr(>F)
Accessions 7 1.5526e+16 2.2180e+15 23.1059 < 2.2e-16 ***
Developmental_stage 2 2.2271e+15 1.1135e+15 11.6002 3.61e-05 ***
repetition 3 9.2288e+13 3.0763e+13 0.3205 0.8105
Residuals 83 7.9675e+15 9.5993e+13
---
Signif. codes: 0 '***' 0.001 '**' 0.01 '*' 0.05 '.' 0.1 ' ' 1

## Lut7R model 2

modelLut=lm(Lut7R~Accessions*Developmental_stage, data= sup6)
res=residuals(modelLut)

### Postulates verification

ks.test(res, "pnorm", 0, sd(res))

Exact one-sample Kolmogorov-Smirnov test

data: res
D = 0.23286, p-value = 4.632e-05
alternative hypothesis: two-sided

plot(modelLut,1)


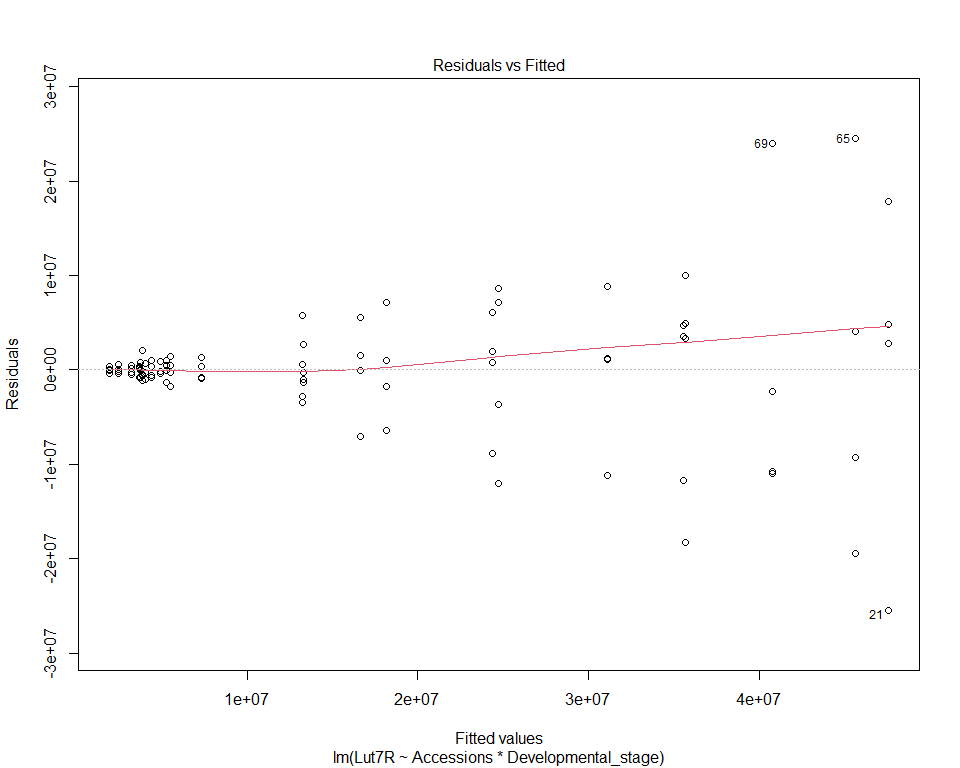


plot(modelLut,2)


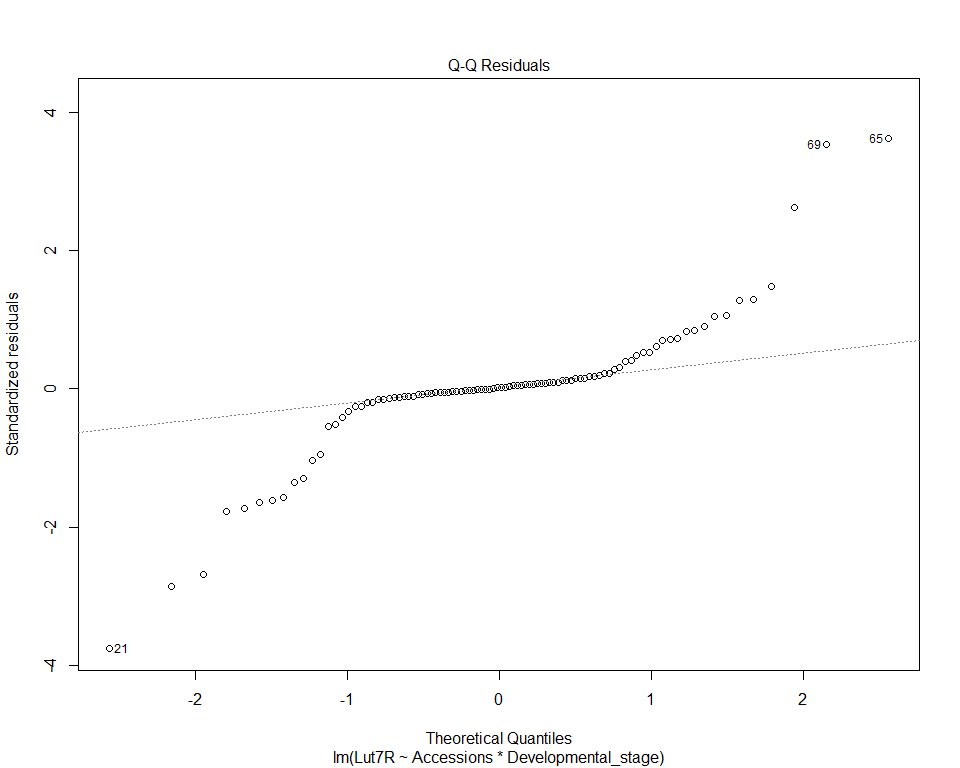


###

### Box-cox transformation

summary(p2 <- powerTransform(modelLut))
sup6_bc2 <- transform(sup6, Lut7R_bc=bcPower(Lut7R,coef(p2)))

modelLut_bc <- lm(Lut7R_bc~ Accessions*Developmental_stage, data=sup6_bc2)
ks.test(residuals(modelLut_bc), "pnorm", 0, sd(residuals(modelLut_bc)))

Exact one-sample Kolmogorov-Smirnov test

data: residuals(modelLut_bc)
D = 0.10201, p-value = 0.2529
alternative hypothesis: two-sided

plot(modelLut_bc,1)


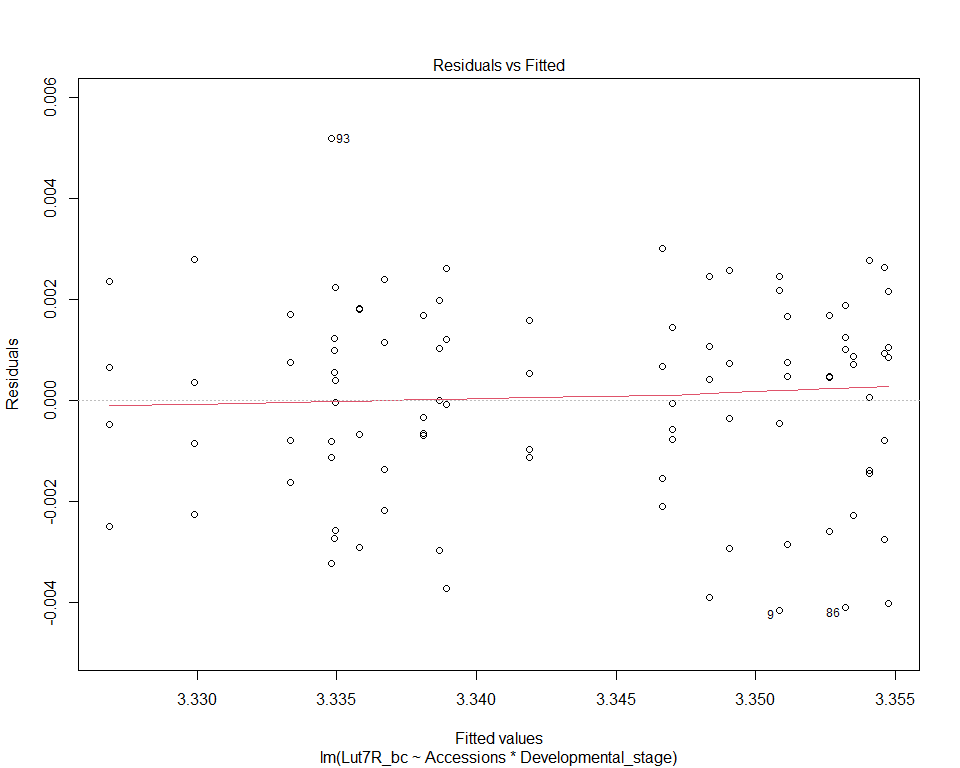


plot(modelLut_bc,2)


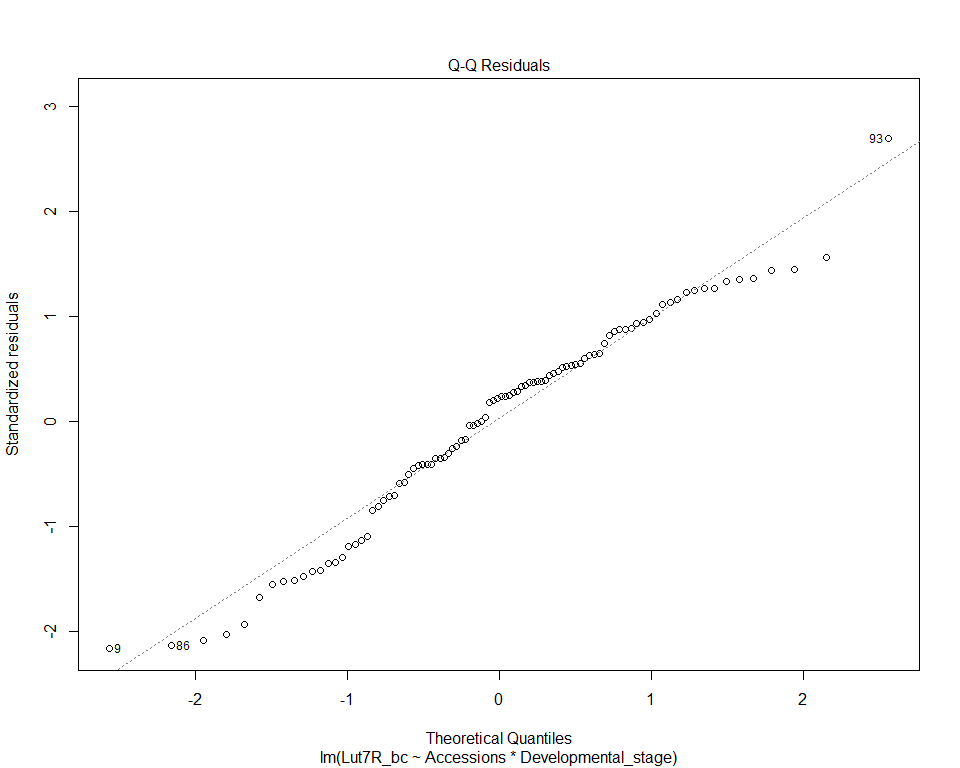


###

### ANOVA

anova(modelLut_bc)

Analysis of Variance Table

Response: Lut7R_bc
 Df Sum Sq Mean Sq F value Pr(>F)
Accessions 7 0.0065076 0.00092966 187.8329 < 2.2e-16 ***
Developmental_stage 2 0.0001990 0.00009949 20.1007 1.159e-07 ***
Accessions:Developmental_stage 14 0.0004740 0.00003386 6.8412 1.224e-08 ***
Residuals 72 0.0003564 0.00000495
---
Signif. codes: 0 '***' 0.001 '**' 0.01 '*' 0.05 '.' 0.1 ' ' 1

## Pairwise comparison

em_acc=emmeans(modelLut_bc, ~ Accessions|Developmental_stage, adjust = "tukey")
cld_results <- cld(em_acc, Letters = letters, adjust = "tukey")
cld_results

Developmental_stage = 2 Leaves:
 Accessions emmean SE df lower.CL upper.CL .group
 PRESTO 3.333 0.001112 72 3.330 3.336 a
 H1 3.336 0.001112 72 3.333 3.339 a
 BRILLYANCE 3.337 0.001112 72 3.334 3.340 a
 VALOR 3.338 0.001112 72 3.335 3.341 a
 I2 3.347 0.001112 72 3.344 3.350 b
 A92 3.347 0.001112 72 3.344 3.350 b
 BOLERO 3.348 0.001112 72 3.345 3.351 b
 B18 3.349 0.001112 72 3.346 3.352 b

Developmental_stage = 6 Leaves:
 Accessions emmean SE df lower.CL upper.CL .group
 PRESTO 3.327 0.001112 72 3.324 3.330 a
 H1 3.330 0.001112 72 3.327 3.333 ab
 VALOR 3.335 0.001112 72 3.332 3.338 bc
 BRILLYANCE 3.339 0.001112 72 3.336 3.342 c
 BOLERO 3.351 0.001112 72 3.348 3.354 d
 I2 3.353 0.001112 72 3.350 3.356 d
 A92 3.354 0.001112 72 3.351 3.357 d
 B18 3.355 0.001112 72 3.351 3.358 d

Developmental_stage = 10 Leaves:
 Accessions emmean SE df lower.CL upper.CL .group
 PRESTO 3.335 0.001112 72 3.332 3.338 a
 H1 3.335 0.001112 72 3.332 3.338 a
 VALOR 3.339 0.001112 72 3.336 3.342 ab
 BRILLYANCE 3.342 0.001112 72 3.339 3.345 b
 BOLERO 3.351 0.001112 72 3.348 3.354 c
 B18 3.353 0.001112 72 3.350 3.356 c
 A92 3.354 0.001112 72 3.350 3.357 c
 I2 3.355 0.001112 72 3.352 3.358 c

Confidence level used: 0.95
Conf-level adjustment: sidak method for 8 estimates
P value adjustment: tukey method for comparing a family of 8 estimates
significance level used: alpha = 0.05
NOTE: If two or more means share the same grouping symbol,
 then we cannot show them to be different.
 But we also did not show them to be the same.

em_dvl=emmeans(modelLut_bc, ~ Developmental_stage|Accessions, adjust = "tukey")
cld_results2 <- cld(em_dvl, Letters = letters, adjust = "tukey")
cld_df <- as.data.frame(cld_results2)
cld_df_ordered <- cld_df %>%
 arrange(factor(Accessions, levels = levels(sup6$Accessions)),
 factor(Developmental_stage, levels = levels(sup6$Developmental_stage)))
cld_df_ordered

Accessions = PRESTO:
 Developmental_stage emmean SE df lower.CL upper.CL .group
 2 Leaves 3.333337 0.001112363 72 3.330618 3.336057 b
 6 Leaves 3.326875 0.001112363 72 3.324156 3.329595 a
 10 Leaves 3.334905 0.001112363 72 3.332185 3.337624 b

Accessions = H1:
 Developmental_stage emmean SE df lower.CL upper.CL .group
 2 Leaves 3.335806 0.001112363 72 3.333087 3.338525 b
 6 Leaves 3.329906 0.001112363 72 3.327187 3.332626 a
 10 Leaves 3.334964 0.001112363 72 3.332245 3.337684 b

Accessions = A92:
 Developmental_stage emmean SE df lower.CL upper.CL .group
 2 Leaves 3.346999 0.001112363 72 3.344280 3.349718 a
 6 Leaves 3.354074 0.001112363 72 3.351354 3.356793 b
 10 Leaves 3.353504 0.001112363 72 3.350785 3.356223 b

Accessions = B18:
 Developmental_stage emmean SE df lower.CL upper.CL .group
 2 Leaves 3.349039 0.001112363 72 3.346320 3.351758 a
 6 Leaves 3.354601 0.001112363 72 3.351881 3.357320 b
 10 Leaves 3.352650 0.001112363 72 3.349931 3.355369 ab

Accessions = BOLERO:
 Developmental_stage emmean SE df lower.CL upper.CL .group
 2 Leaves 3.348328 0.001112363 72 3.345608 3.351047 a
 6 Leaves 3.351115 0.001112363 72 3.348396 3.353834 a
 10 Leaves 3.350848 0.001112363 72 3.348129 3.353568 a

Accessions = BRILLYANCE:
 Developmental_stage emmean SE df lower.CL upper.CL .group
 2 Leaves 3.336695 0.001112363 72 3.333975 3.339414 a
 6 Leaves 3.338679 0.001112363 72 3.335960 3.341398 ab
 10 Leaves 3.341914 0.001112363 72 3.339194 3.344633 b

Accessions = VALOR:
 Developmental_stage emmean SE df lower.CL upper.CL .group
 2 Leaves 3.338095 0.001112363 72 3.335376 3.340814 ab
 6 Leaves 3.334810 0.001112363 72 3.332091 3.337529 a
 10 Leaves 3.338932 0.001112363 72 3.336213 3.341651 b

Accessions = I2:
 Developmental_stage emmean SE df lower.CL upper.CL .group
 2 Leaves 3.346671 0.001112363 72 3.343952 3.349390 a
 6 Leaves 3.353191 0.001112363 72 3.350472 3.355911 b
 10 Leaves 3.354750 0.001112363 72 3.352030 3.357469 b

Confidence level used: 0.95
Conf-level adjustment: sidak method for 3 estimates
P value adjustment: tukey method for comparing a family of 3 estimates
significance level used: alpha = 0.05
NOTE: If two or more means share the same grouping symbol,
 then we cannot show them to be different.
 But we also did not show them to be the same.

# Chry7R

## Chry7R Model 1

modelChry1=lm(Chry7R~Accessions+Developmental_stage+ repetition, data= sup6)
res=residuals(modelChry1)
ks.test(res,"pnorm", 0, sd(res))

Exact one-sample Kolmogorov-Smirnov test

data: res
D = 0.1605, p-value = 0.01255
alternative hypothesis: two-sided

plot(modelChry1,1)


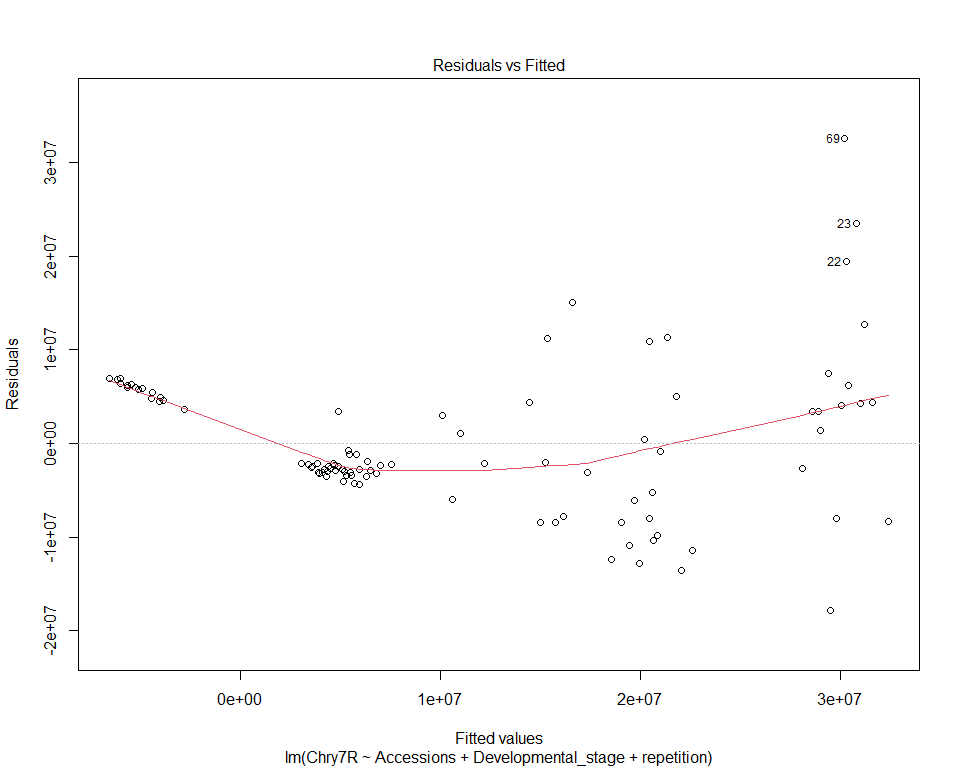


plot(modelChry1,2)


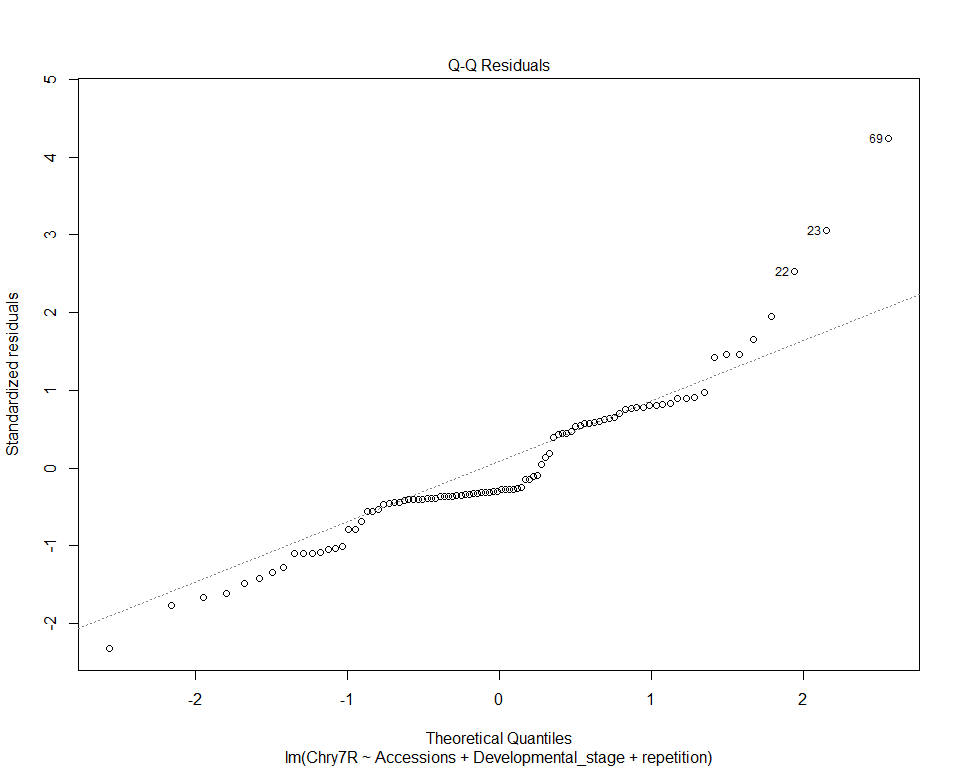


summary(p2 <- powerTransform(modelChry1))
sup6_bc <- transform(sup6, ApiChry1_bc=bcPower(Chry7R,coef(p2)))

modelChry1_bc <- lm(ApiChry1_bc~ Accessions+Developmental_stage+ repetition, data=sup6_bc)
ks.test(residuals(modelChry1_bc), "pnorm", 0, sd(residuals(modelChry1_bc)))

Exact one-sample Kolmogorov-Smirnov test

data: residuals(modelChry1_bc)
D = 0.0594, p-value = 0.867
alternative hypothesis: two-sided

plot(modelChry1_bc,1)


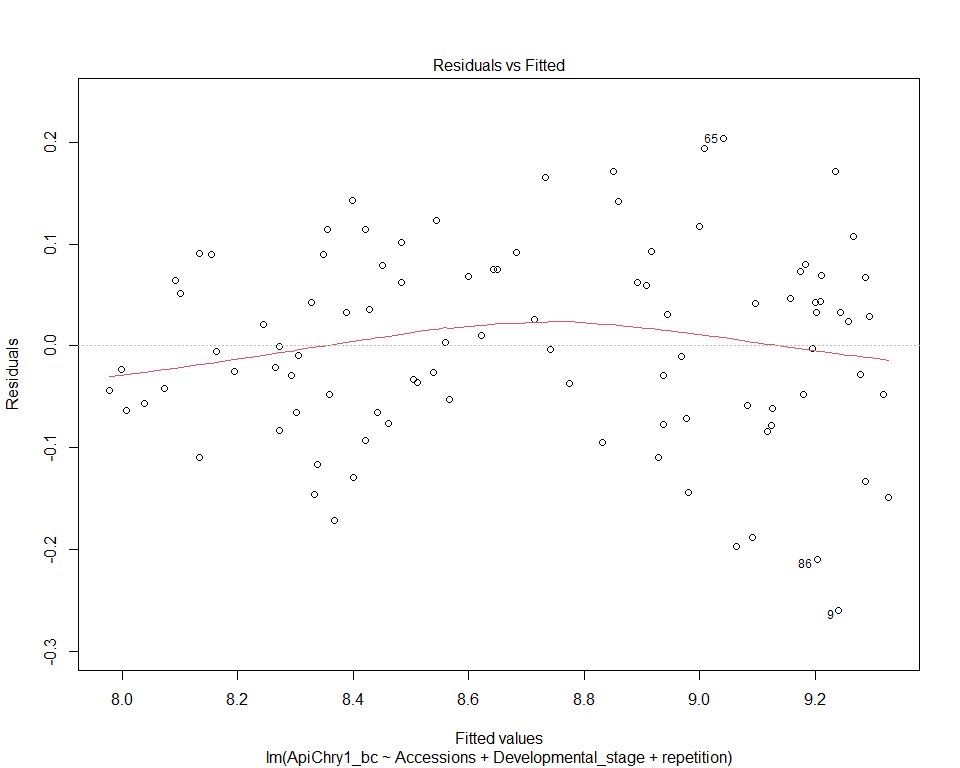


plot(modelChry1_bc,2)


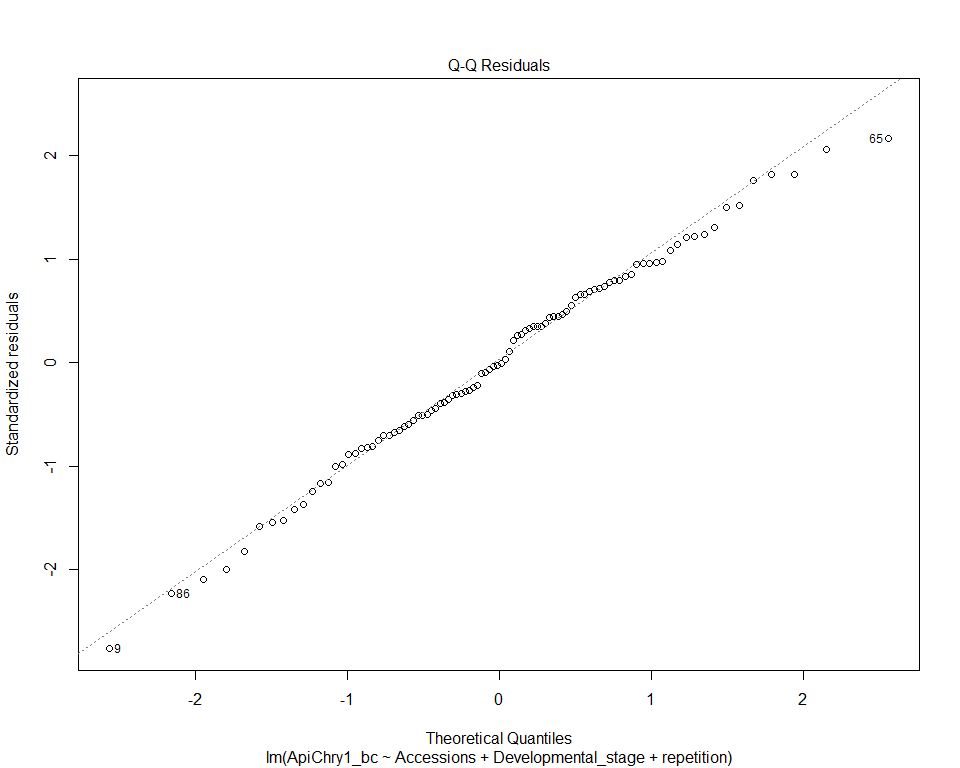


## Chry7R model 2

modelChry=lm(Chry7R~Accessions*Developmental_stage, data= sup6)
res=residuals(modelChry)

### Postulates verification

ks.test(res, "pnorm", 0, sd(res))

Exact one-sample Kolmogorov-Smirnov test
data: res
D = 0.23536, p-value = 3.673e-05
alternative hypothesis: two-sided

plot(modelChry,1)


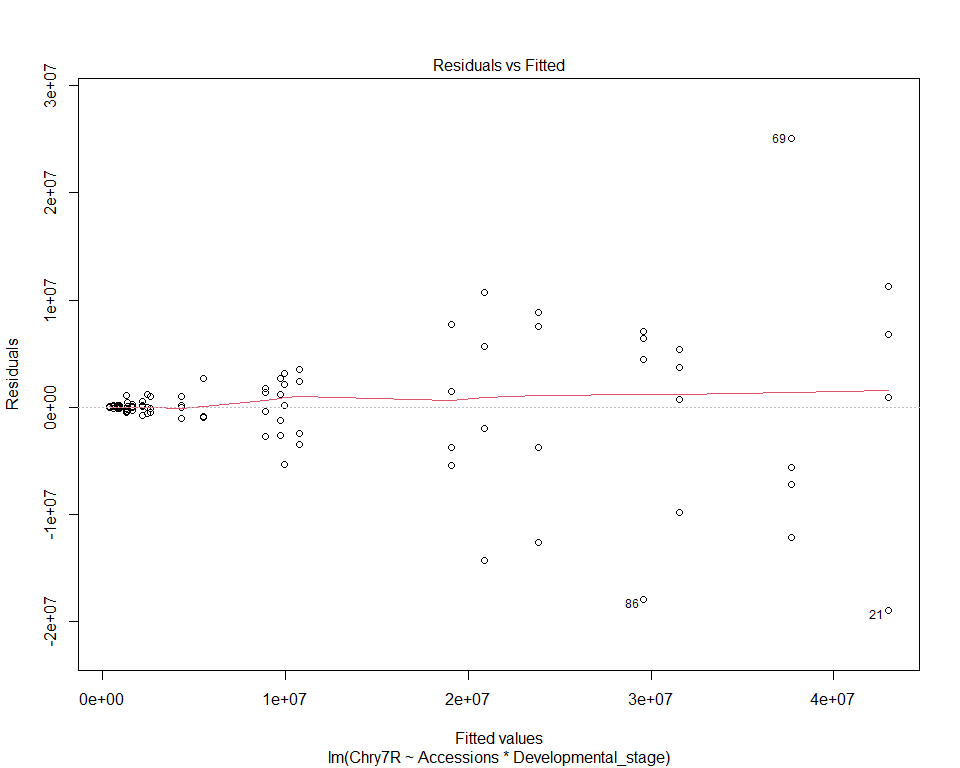


plot(modelChry,2)


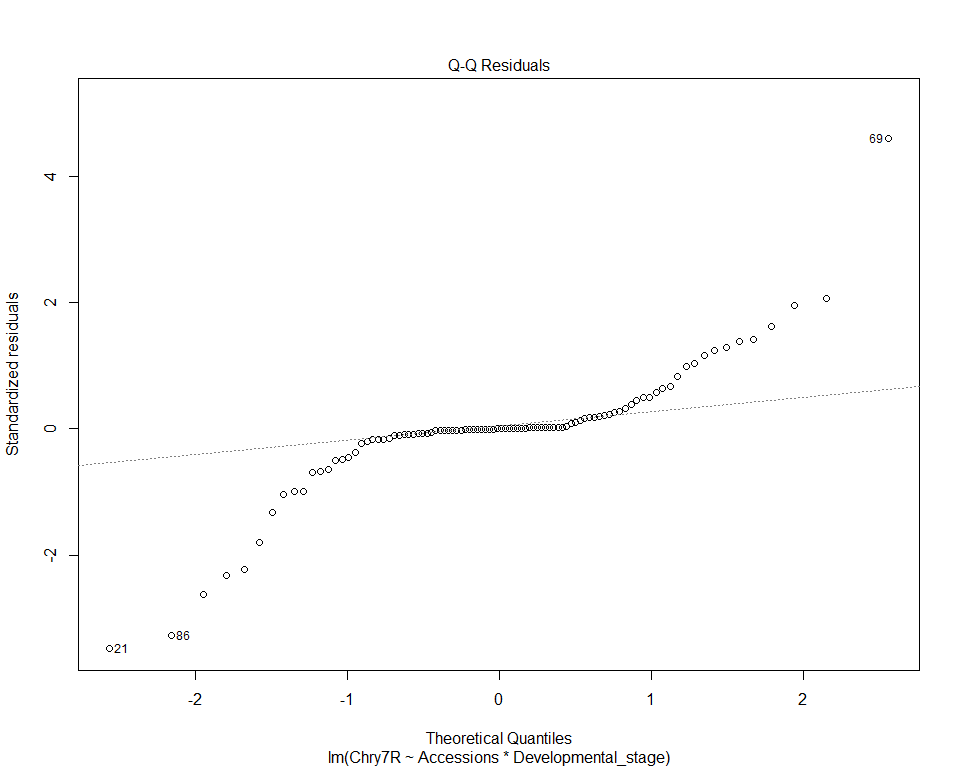


### Box-cox transformation

summary(p2 <- powerTransform(modelChry))
sup6_bc3 <- transform(sup6, Chry7R_bc=bcPower(Chry7R,coef(p2)))

modelChry_bc <- lm(Chry7R_bc~ Accessions*Developmental_stage, data=sup6_bc3)
ks.test(residuals(modelChry_bc), "pnorm", 0, sd(residuals(modelChry_bc)))

Exact one-sample Kolmogorov-Smirnov test
data: residuals(modelChry_bc)
D = 0.068844, p-value = 0.7267
alternative hypothesis: two-sided

plot(modelChry_bc,1)


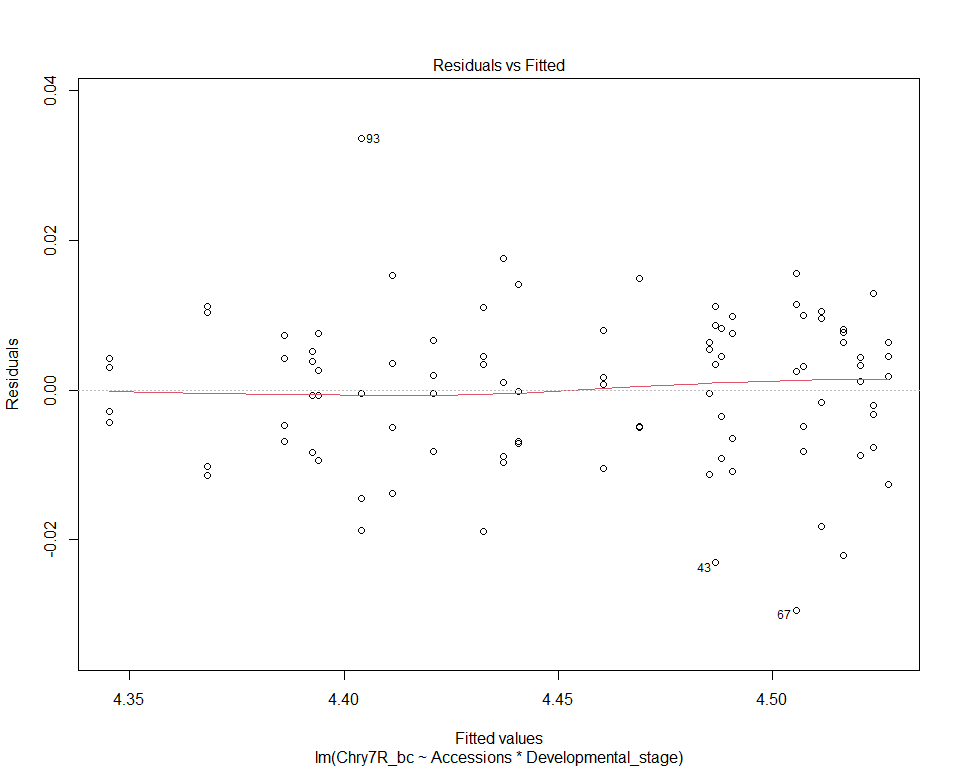


plot(modelChry_bc,2)


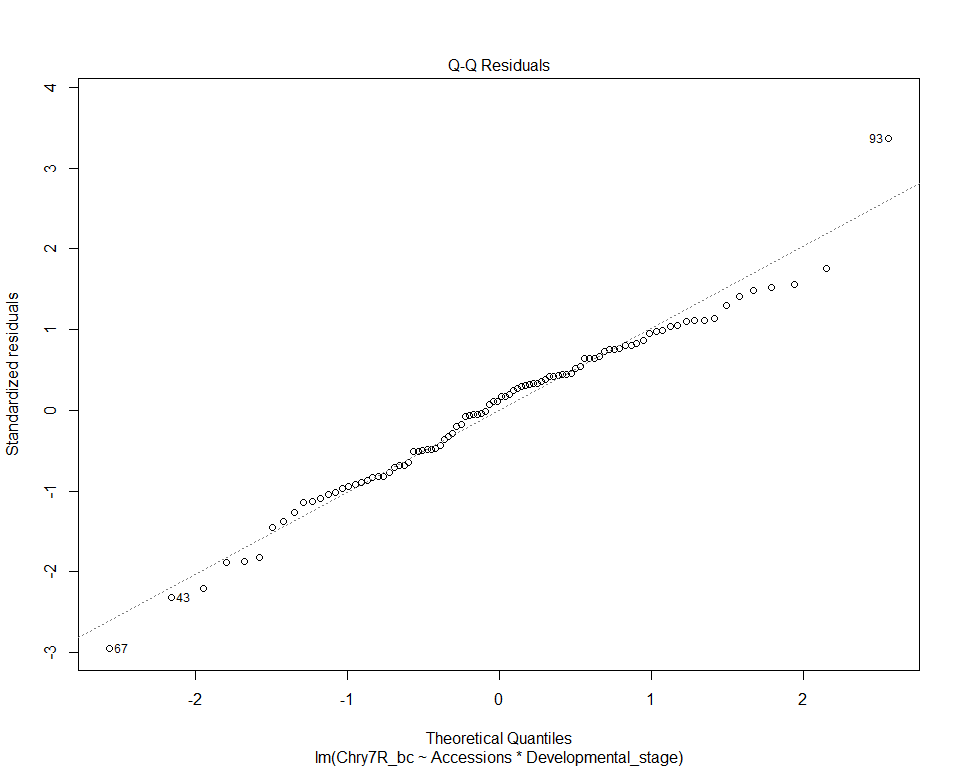


###

### ANOVA

anova(modelChry_bc)

Analysis of Variance Table

Response: Chry7R_bc
 Df Sum Sq Mean Sq F value Pr(>F)
Accessions 7 0.232732 0.033247 249.2429 < 2.2e-16 ***
Developmental_stage 2 0.038970 0.019485 146.0729 < 2.2e-16 ***
Accessions:Developmental_stage 14 0.007212 0.000515 3.8619 7.183e-05 ***
Residuals 72 0.009604 0.000133
---
Signif. codes: 0 '***' 0.001 '**' 0.01 '*' 0.05 '.' 0.1 ' ' 1

## Pairwise comparison

em_acc=emmeans(modelChry_bc, ~ Accessions|Developmental_stage, adjust = "tukey")
cld_results <- cld(em_acc, Letters = letters, adjust = "tukey")
cld_results

Developmental_stage = 2 Leaves:
 Accessions emmean SE df lower.CL upper.CL .group
 PRESTO 4.345 0.005775 72 4.329 4.362 a
 VALOR 4.368 0.005775 72 4.352 4.384 ab
 H1 4.386 0.005775 72 4.370 4.402 b
 BRILLYANCE 4.393 0.005775 72 4.376 4.409 b
 B18 4.469 0.005775 72 4.453 4.485 c
 A92 4.485 0.005775 72 4.469 4.502 c
 BOLERO 4.487 0.005775 72 4.471 4.503 c
 I2 4.488 0.005775 72 4.472 4.504 c

Developmental_stage = 6 Leaves:
 Accessions emmean SE df lower.CL upper.CL .group
 PRESTO 4.394 0.005775 72 4.378 4.410 a
 VALOR 4.404 0.005775 72 4.388 4.420 a
 H1 4.411 0.005775 72 4.395 4.428 a
 BRILLYANCE 4.441 0.005775 72 4.425 4.457 b
 B18 4.506 0.005775 72 4.489 4.522 c
 BOLERO 4.507 0.005775 72 4.491 4.524 c
 I2 4.517 0.005775 72 4.501 4.533 c
 A92 4.524 0.005775 72 4.507 4.540 c

Developmental_stage = 10 Leaves:
 Accessions emmean SE df lower.CL upper.CL .group
 PRESTO 4.421 0.005775 72 4.405 4.437 a
 VALOR 4.433 0.005775 72 4.416 4.449 a
 H1 4.437 0.005775 72 4.421 4.454 ab
 BRILLYANCE 4.461 0.005775 72 4.444 4.477 b
 B18 4.491 0.005775 72 4.474 4.507 c
 BOLERO 4.512 0.005775 72 4.495 4.528 cd
 A92 4.521 0.005775 72 4.504 4.537 d
 I2 4.527 0.005775 72 4.511 4.543 d
Confidence level used: 0.95
Conf-level adjustment: sidak method for 8 estimates
P value adjustment: tukey method for comparing a family of 8 estimates
significance level used: alpha = 0.05
NOTE: If two or more means share the same grouping symbol,
 then we cannot show them to be different.
 But we also did not show them to be the same.

em_dvl=emmeans(modelChry_bc, ~ Developmental_stage|Accessions, adjust = "tukey")
cld_results2 <- cld(em_dvl, Letters = letters, adjust = "tukey")
cld_df <- as.data.frame(cld_results2)
cld_df_ordered <- cld_df %>%
 arrange(factor(Accessions, levels = levels(sup6$Accessions)),
 factor(Developmental_stage, levels = levels(sup6$Developmental_stage)))
cld_df_ordered

Accessions = PRESTO:
 Developmental_stage emmean SE df lower.CL upper.CL .group
 2 Leaves 4.345301 0.005774806 72 4.331184 4.359417 a
 6 Leaves 4.394011 0.005774806 72 4.379894 4.408128 b
 10 Leaves 4.420947 0.005774806 72 4.406830 4.435063 c

Accessions = H1:
 Developmental_stage emmean SE df lower.CL upper.CL .group
 2 Leaves 4.385975 0.005774806 72 4.371858 4.400092 a
 6 Leaves 4.411408 0.005774806 72 4.397291 4.425524 b
 10 Leaves 4.437278 0.005774806 72 4.423161 4.451395 c

Accessions = A92:
 Developmental_stage emmean SE df lower.CL upper.CL .group
 2 Leaves 4.485379 0.005774806 72 4.471263 4.499496 a
 6 Leaves 4.523660 0.005774806 72 4.509543 4.537777 b
 10 Leaves 4.520643 0.005774806 72 4.506526 4.534760 b

Accessions = B18:
 Developmental_stage emmean SE df lower.CL upper.CL .group
 2 Leaves 4.468981 0.005774806 72 4.454864 4.483098 a
 6 Leaves 4.505679 0.005774806 72 4.491562 4.519796 b
 10 Leaves 4.490682 0.005774806 72 4.476565 4.504799 b

Accessions = BOLERO:
 Developmental_stage emmean SE df lower.CL upper.CL .group
 2 Leaves 4.486853 0.005774806 72 4.472736 4.500970 a
 6 Leaves 4.507337 0.005774806 72 4.493220 4.521453 b
 10 Leaves 4.511576 0.005774806 72 4.497459 4.525693 b

Accessions = BRILLYANCE:
 Developmental_stage emmean SE df lower.CL upper.CL .group
 2 Leaves 4.392540 0.005774806 72 4.378423 4.406657 a
 6 Leaves 4.440731 0.005774806 72 4.426614 4.454848 b
 10 Leaves 4.460595 0.005774806 72 4.446478 4.474712 c

Accessions = VALOR:
 Developmental_stage emmean SE df lower.CL upper.CL .group
 2 Leaves 4.368067 0.005774806 72 4.353951 4.382184 a
 6 Leaves 4.404177 0.005774806 72 4.390060 4.418294 b
 10 Leaves 4.432577 0.005774806 72 4.418460 4.446693 c

Accessions = I2:
 Developmental_stage emmean SE df lower.CL upper.CL .group
 2 Leaves 4.488219 0.005774806 72 4.474102 4.502336 a
 6 Leaves 4.516723 0.005774806 72 4.502606 4.530840 b
 10 Leaves 4.527168 0.005774806 72 4.513051 4.541285 b

Confidence level used: 0.95
Conf-level adjustment: sidak method for 3 estimates
P value adjustment: tukey method for comparing a family of 3 estimates
significance level used: alpha = 0.05
NOTE: If two or more means share the same grouping symbol,
 then we cannot show them to be different.
 But we also did not show them to be the same.
