# Supplementary material for: Flavonoid compounds as a way to identify sources of carrot resistance to Alternaria leaf blight
Source: Mol Breed. 2025 Jun 14;45(6):55. doi: 10.1007/s11032-025-01573-1 (PMC12167411; doi:10.1007/s11032-025-01573-1)

**THREE FLAVONOIDS BIOMARKERS OF CARROT RESISTANCE TO ALTERNARIA LEAF BLIGHT: ACCUMULATION PATTERN AT DIFFERENT PHENOLOGICAL STAGES AND CONSISTENCY ACROSS DIVERSE GENETIC BACKGROUNDS**

**MOLECULAR BREEDING**

Marie Louisa Ramaroson*^1^, Claude Emmanuel Koutouan*^1^, Angelina El Ghaziri^1^, Raymonde Baltenweck^2^, Patricia Claudel^2^, Philippe Hugueney^2^, Sébastien Huet^1^, Anita Suel^1^, Linda Voisine^1^, Mathilde Briard^1^, Jean Jacques Helesbeux^3^, Latifa Hamama^1^, Valérie le Clerc^1^, Emmanuel Geoffriau^1,§^

1 Institut Agro, Université d’Angers, INRAE, IRHS, SFR 4207 QUASAV, Angers, France

2 Université de Strasbourg, INRAE, SVQV UMR-A 1131, F-68000 Colmar, France

3 Université de Strasbourg, INRAE, SVQV UMR-A 1131, F-68000 Colmar, France

§ Correspondence: emmanuel.geoffriau@institut-agro.fr; Tel : +33-(0)2 41 22 54 31

* The first two authors contributed equally to the paper

Online Resource 6: Selection process of the accessions the most highly-accumulating Api 7R, Lut 7R and Chry 7R and disease severity analysis for Trial 4

Table of contents

[Accessions selection 1](#_Toc191882062)

[Api7R 1](#_Toc191882063)

[Lut7R 2](#_Toc191882064)

[Chry7R 2](#_Toc191882065)

[Intersection of the three resulting accessions selection 3](#_Toc191882066)

[Analysis of disease severity 3](#_Toc191882067)

[Linear model with repetition 3](#_Toc191882068)

[Linear model without repetition 4](#_Toc191882069)

#

# Accessions selection

## Api7R

Extraction of accessions with Api content higher than 85% of maximum

ApplyQuintiles <- function(x) {
 cut(x, breaks = c(quantile(dfApiM$MeanApi,
 probs = c(0, 0.75, 0.8, 0.85, 1))),
 labels = c("Api7R content <75%",
 "Api7R content [75-80%]", "Api7R content [80-85%]",
 "Api7R content >85%"), include.lowest = TRUE)
}

dfApiM$Api7R_classe <- sapply(dfApiM$MeanApi, ApplyQuintiles)
table(dfApiM$Api7R_classe)

Api7R content <75% Api7R content [75-80%] Api7R content [80-85%] Api7R content >85%
 73 4 5 15

AvecApiQuantile=dfApiM[dfApiM$Api7R_classe=="Api7R content >85%",1]

## Lut7R

Extraction of accessions with Lut content higher than 85% of maximum

ApplyQuintiles <- function(x) {
 cut(x, breaks=c(quantile(dfLutM$MeanLut,
 probs = c(0, 0.75, 0.8, 0.85, 1))),
 labels=c("Lut7R content <75%", "Lut7R content [75-80]",
 "Lut7R content [80-85]", "Lut7R content >85%"), include.lowest=TRUE)
}


dfLutM$Lut7R_classe <- sapply(dfLutM$MeanLut, ApplyQuintiles)
table(dfLutM$Lut7R_classe)

Lut7R content <75% Lut7R content [75-80] Lut7R content [80-85] Lut7R content >85%
 73 4 5 15

AvecLutQuantile=dfLutM[dfLutM$Lut7R_classe=="Lut7R content >85%",1]

## Chry7R

Extraction of accessions with Chry content higher than 85% of maximum

ApplyQuintiles <- function(x) {
 cut(x, breaks=c(quantile(dfChryM$MeanChry,
 probs = c(0, 0.75, 0.8, 0.85, 1))),
 labels=c("Chry7R content <75%", "Chry7R content [75-80]",
 "Chry7R content [80-85]", "Chry7R content >85%"), include.lowest=TRUE)
}


dfChryM$Chry7R_classe <- sapply(dfChryM$MeanChry, ApplyQuintiles)
table(dfChryM$Chry7R_classe)

Chry7R content <75% Chry7R content [75-80] Chry7R content [80-85] Chry7R content >85%
 73 4 5 15

AvecChryQuantile=dfChryM[dfChryM$Chry7R_classe=="Chry7R content >85%",1]

## Intersection of the three resulting accessions selection

With85 <- names(which(table(c(
 as.character(AvecApiQuantile[[1]]),
 as.character(AvecLutQuantile[[1]]),
 as.character(AvecChryQuantile[[1]])
)) == 3))

With85

[1] "AKT 36" "Kink 57" "Lob 62" "Lob 66" "Lob 69" "Luc 86" "Reg 21" "Yam 99"

#

# Analysis of disease severity

## Linear model with repetition

library(lme4)
library(lmerTest)
mod1=lmer(notation2~accessions+(1|repetition), data=meta2)
shapiro.test(residuals(mod1))

Shapiro-Wilk normality test

data: residuals(mod1)
W = 0.98604, p-value = 0.8945

ranova(mod1)

ANOVA-like table for random-effects: Single term deletions

Model:
notation2 ~ accessions + (1 | repetition)
 npar logLik AIC LRT Df Pr(>Chisq)
<none> 12 -42.469 108.94
(1 | repetition) 11 -42.648 107.30 0.35843 1 0.5494

##

## Linear model without repetition

mod=lm(notation2~accessions, data=meta2)
shapiro.test(residuals(mod))

Shapiro-Wilk normality test

data: residuals(mod)
W = 0.97202, p-value = 0.4159

bartlett.test(residuals(mod), g=meta2$accessions)

Bartlett test of homogeneity of variances

data: residuals(mod) and meta2$accessions
Bartlett's K-squared = 5.8161, df = 9, p-value = 0.7582

anova(mod)

Analysis of Variance Table

Response: notation2
 Df Sum Sq Mean Sq F value Pr(>F)
accessions 9 20.1 2.23333 3.5263 0.004371 **
Residuals 30 19.0 0.63333
---
Signif. codes: 0 '***' 0.001 '**' 0.01 '*' 0.05 '.' 0.1 ' ' 1

library(emmeans)
emm <- emmeans(mod, ~ accessions)
pairs(emm, adjust = "tukey")

contrast estimate SE df t.ratio p.value
 AKT 36 - BOLERO -0.25 0.563 30 -0.444 1.0000
 AKT 36 - Kink 57 -1.00 0.563 30 -1.777 0.7436
 AKT 36 - Lob 62 -1.00 0.563 30 -1.777 0.7436
 AKT 36 - Lob 66 -0.25 0.563 30 -0.444 1.0000
 AKT 36 - Lob 69 -0.50 0.563 30 -0.889 0.9957
 AKT 36 - Luc 86 -0.75 0.563 30 -1.333 0.9376
 AKT 36 - Presto -2.50 0.563 30 -4.443 0.0038
 AKT 36 - Reg 21 0.00 0.563 30 0.000 1.0000
 AKT 36 - Yam 99 -0.25 0.563 30 -0.444 1.0000
 BOLERO - Kink 57 -0.75 0.563 30 -1.333 0.9376
 BOLERO - Lob 62 -0.75 0.563 30 -1.333 0.9376
 BOLERO - Lob 66 0.00 0.563 30 0.000 1.0000
 BOLERO - Lob 69 -0.25 0.563 30 -0.444 1.0000
 BOLERO - Luc 86 -0.50 0.563 30 -0.889 0.9957
 BOLERO - Presto -2.25 0.563 30 -3.998 0.0120
 BOLERO - Reg 21 0.25 0.563 30 0.444 1.0000
 BOLERO - Yam 99 0.00 0.563 30 0.000 1.0000
 Kink 57 - Lob 62 0.00 0.563 30 0.000 1.0000
 Kink 57 - Lob 66 0.75 0.563 30 1.333 0.9376
 Kink 57 - Lob 69 0.50 0.563 30 0.889 0.9957
 Kink 57 - Luc 86 0.25 0.563 30 0.444 1.0000
 Kink 57 - Presto -1.50 0.563 30 -2.666 0.2326
 Kink 57 - Reg 21 1.00 0.563 30 1.777 0.7436
 Kink 57 - Yam 99 0.75 0.563 30 1.333 0.9376
 Lob 62 - Lob 66 0.75 0.563 30 1.333 0.9376
 Lob 62 - Lob 69 0.50 0.563 30 0.889 0.9957
 Lob 62 - Luc 86 0.25 0.563 30 0.444 1.0000
 Lob 62 - Presto -1.50 0.563 30 -2.666 0.2326
 Lob 62 - Reg 21 1.00 0.563 30 1.777 0.7436
 Lob 62 - Yam 99 0.75 0.563 30 1.333 0.9376
 Lob 66 - Lob 69 -0.25 0.563 30 -0.444 1.0000
 Lob 66 - Luc 86 -0.50 0.563 30 -0.889 0.9957
 Lob 66 - Presto -2.25 0.563 30 -3.998 0.0120
 Lob 66 - Reg 21 0.25 0.563 30 0.444 1.0000
 Lob 66 - Yam 99 0.00 0.563 30 0.000 1.0000
 Lob 69 - Luc 86 -0.25 0.563 30 -0.444 1.0000
 Lob 69 - Presto -2.00 0.563 30 -3.554 0.0358
 Lob 69 - Reg 21 0.50 0.563 30 0.889 0.9957
 Lob 69 - Yam 99 0.25 0.563 30 0.444 1.0000
 Luc 86 - Presto -1.75 0.563 30 -3.110 0.0974
 Luc 86 - Reg 21 0.75 0.563 30 1.333 0.9376
 Luc 86 - Yam 99 0.50 0.563 30 0.889 0.9957
 Presto - Reg 21 2.50 0.563 30 4.443 0.0038
 Presto - Yam 99 2.25 0.563 30 3.998 0.0120
 Reg 21 - Yam 99 -0.25 0.563 30 -0.444 1.0000

P value adjustment: tukey method for comparing a family of 10 estimates

library(multcomp)
res=cld(emm, adjust = "tukey", Letters = letters)
res

accessions emmean SE df lower.CL upper.CL .group
 Reg 21 5.50 0.398 30 4.30 6.70 a
 AKT 36 5.50 0.398 30 4.30 6.70 a
 Yam 99 5.75 0.398 30 4.55 6.95 a
 BOLERO 5.75 0.398 30 4.55 6.95 a
 Lob 66 5.75 0.398 30 4.55 6.95 a
 Lob 69 6.00 0.398 30 4.80 7.20 a
 Luc 86 6.25 0.398 30 5.05 7.45 ab
 Lob 62 6.50 0.398 30 5.30 7.70 ab
 Kink 57 6.50 0.398 30 5.30 7.70 ab
 Presto 8.00 0.398 30 6.80 9.20 b

Confidence level used: 0.95
Conf-level adjustment: sidak method for 10 estimates
P value adjustment: tukey method for comparing a family of 10 estimates
significance level used: alpha = 0.05
NOTE: If two or more means share the same grouping symbol,
 then we cannot show them to be different.
 But we also did not show them to be the same.


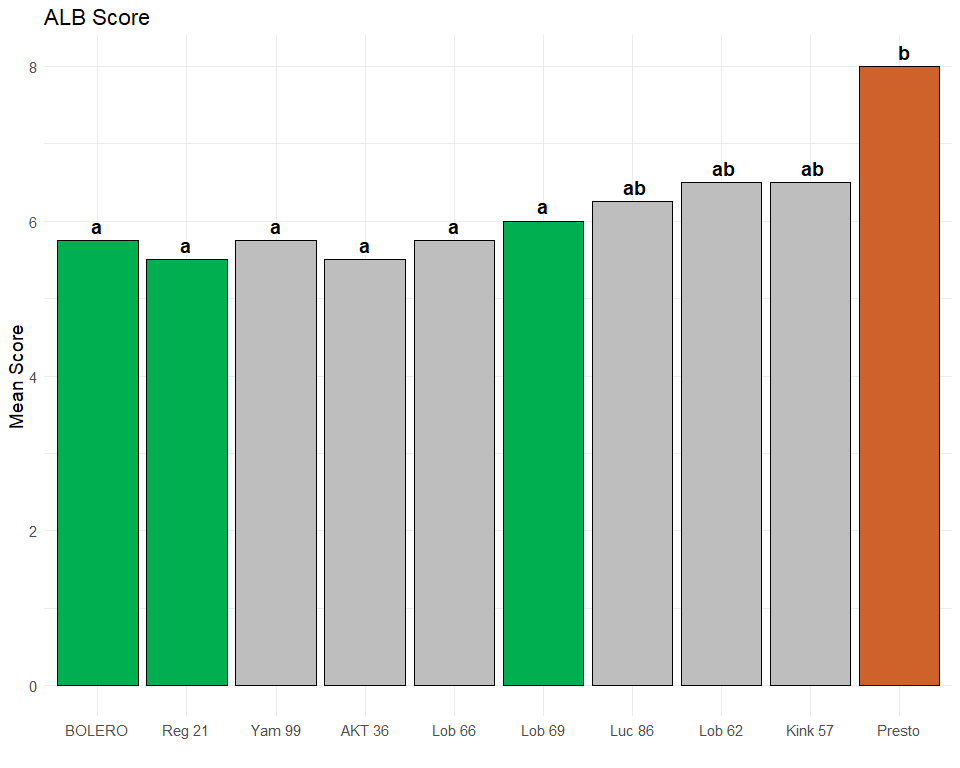

Supplement: Supplementary file 6 — Supplementary Material 6 [file 11032_2025_1573_MOESM6_ESM.docx]
