# Supplementary material for: Flavonoid compounds as a way to identify sources of carrot resistance to Alternaria leaf blight
Source: Mol Breed. 2025 Jun 14;45(6):55. doi: 10.1007/s11032-025-01573-1 (PMC12167411; doi:10.1007/s11032-025-01573-1)
Supplement: Supplementary file 7 — Supplementary Material 7 [file 11032_2025_1573_MOESM7_ESM.docx]

**Three flavonoids biomarkers of Carrot resistance to Alternaria leaf blight: ACCUMULATION PATTERN AT DIFFERENT PHENOLOGICAL STAGES AND CONSISTENCY ACROSS DIVERSE GENETIC BACKGROUNDS**

**Molecular breeding**

Marie Louisa Ramaroson*^1^, Claude Emmanuel Koutouan*^1^, Angelina El Ghaziri^1^, Raymonde Baltenweck^2^, Patricia Claudel^2^, Philippe Hugueney^2^, Sébastien Huet^1^, Anita Suel^1^, Linda Voisine^1^, Mathilde Briard^1^, Jean Jacques Helesbeux^3^, Latifa Hamama^1^, Valérie le Clerc^1^, Emmanuel Geoffriau^1,§^

1 Institut Agro, Université d’Angers, INRAE, IRHS, SFR 4207 QUASAV, Angers, France

2 Université de Strasbourg, INRAE, SVQV UMR-A 1131, F-68000 Colmar, France

3 Université de Strasbourg, INRAE, SVQV UMR-A 1131, F-68000 Colmar, France

§ Correspondence: [emmanuel.geoffriau@institut-agro.fr](mailto:emmanuel.geoffriau@institut-agro.fr); Tel : +33-(0)2 41 22 54 31

* The first two authors contributed equally to the paper

Online Resource 7: Correlations between molecules

Correlation between Api7R, Lut7R and Chry7R contents for all data of Trial 1, Trial 2, Trial 3 and Trial 4. The larger and the darker the blue circle, the higher the correlation between metabolite contents. The Pearson correlation coefficients are denoted by 'r,' and the calculated p-values associated with the null hypothesis (that the correlation is zero) are denoted by 'p.'


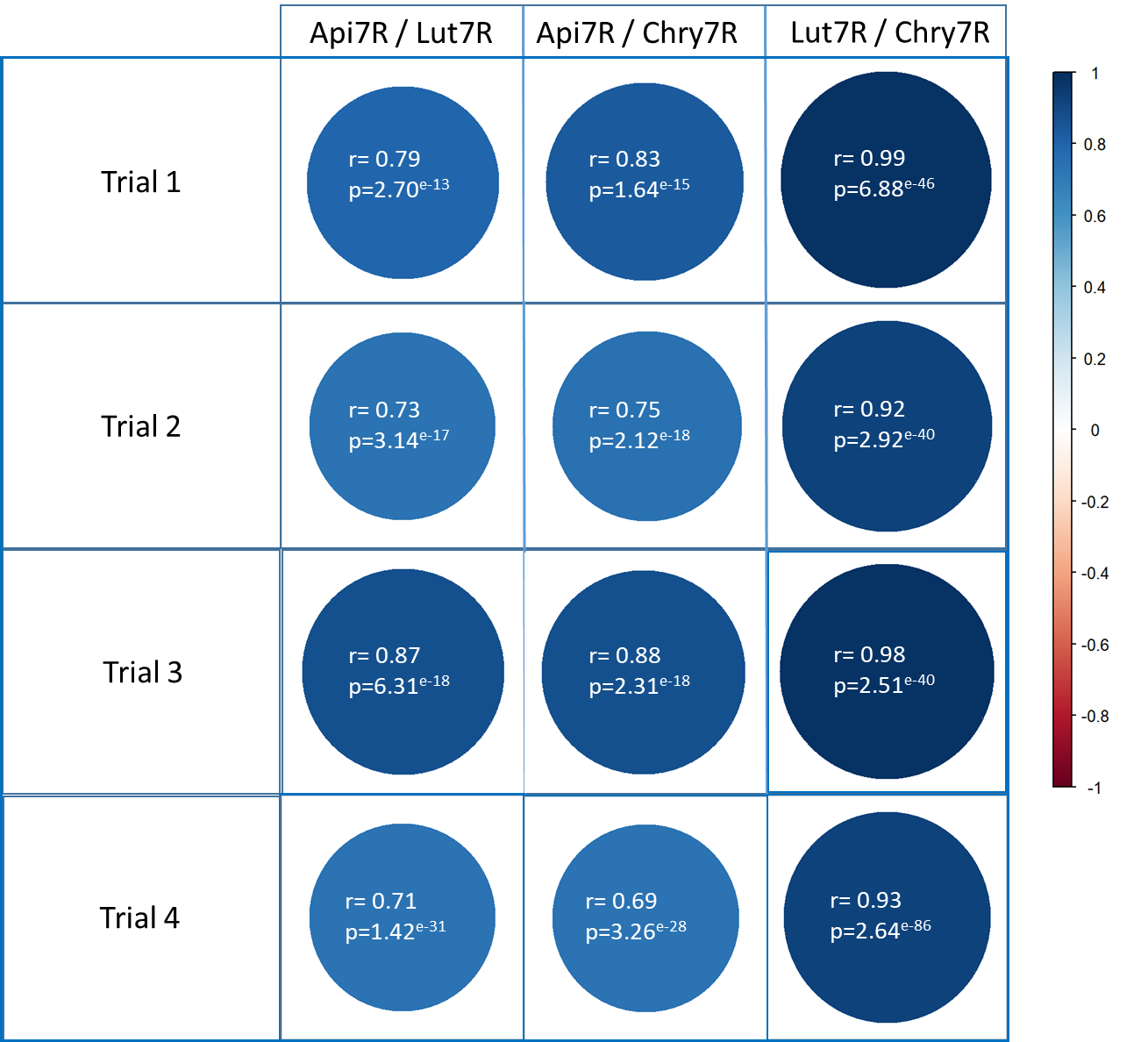
*
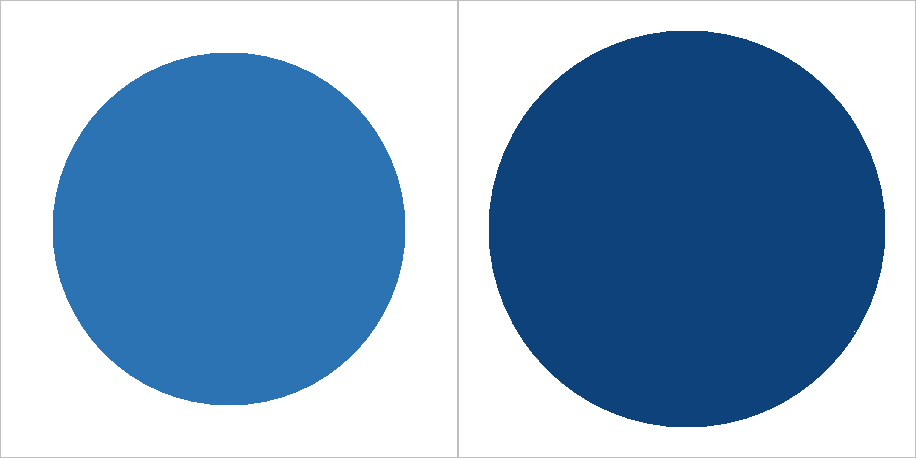

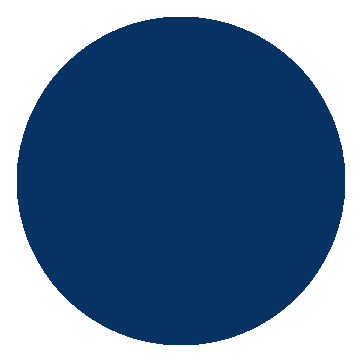

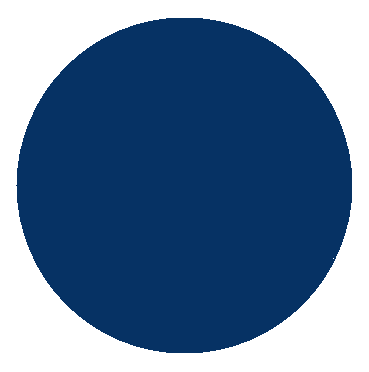

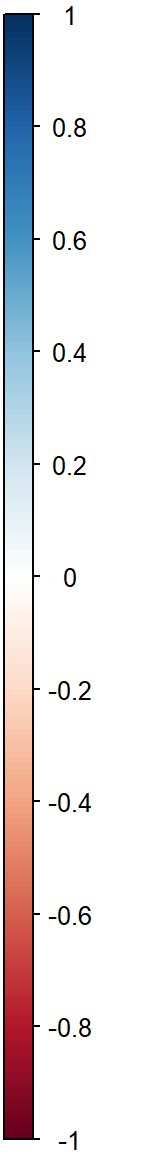

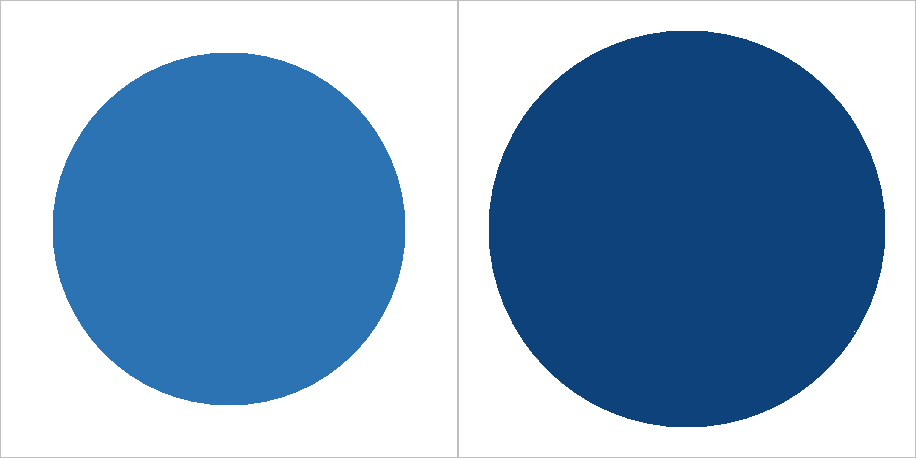
*

Api7R / Lut7R

Api7R / Chry7R

Lut7R / Chry7R

r= 0.79

p=2.70^e-13^

r= 0.83

p=1.64^e-15^

r= 0.99

p=6.88^e-46^
